# The potential use of glycosyl-transferase inhibitors for targeted reduction of *S. mutans* biofilms in dental materials

**DOI:** 10.1038/s41598-023-39125-2

**Published:** 2023-07-23

**Authors:** Polliana Mendes Candia Scaffa, Alexander Kendall, Marcelo Yudi Icimoto, Ana Paula Piovezan Fugolin, Matthew G. Logan, Andre G. DeVito-Moraes, Steven H. Lewis, Hua Zhang, Hui Wu, Carmem S. Pfeifer

**Affiliations:** 1 Division of Biomaterial and Biomedical Sciences, Department of Oral Rehabilitation and Biosciences, Oregon Health & Science University, OHSU, 2730 S Moody Ave., Portland, OR 97201 USA; 2 Department of Biophysics, Federal University of Sao Paulo, UNIFESP-EPM, R. Sena Madureira, 1500, Sao Paulo, SP 04021-001 Brazil

**Keywords:** Oral diseases, Microbiology, Chemistry, Materials science

## Abstract

*Streptococcus mutans* is the primary oral caries-forming bacteria, adept at producing “sticky” biofilms via the synthesis of insoluble extracellular polysaccharides (EPS), catalyzed by glucosyltransferases (GTFs). To circumvent the use of broad-spectrum antibiotics to combat these bacteria, this study sought to modify existing EPS-targeting small molecules with the ultimate goal of producing anti-biofilm polymer surfaces specifically targeting *S. mutans*. To achieve this, a known GTF inhibitor (G43) was modified with methoxy or tetraethyleneglycol substitutions in different positions (nine derivatives, tested at 50-µM) to pinpoint potential sites for future methacrylate functionalization, and then assessed against single-species *S. mutans* biofilms. As expected, the compounds did not diminish the bacterial viability. In general, the compounds with methoxy substitution were not effective in reducing EPS formation, whereas the tetraethyleneglycol substitution (G43-C3-TEG) led to a decrease in the concentration of insoluble EPS, although the effect is less pronounced than for the parent G43. This aligns with the reduced GTF-C activity observed at different concentrations of G43-C3-TEG, as well as the consequent decrease in EPS formation, and notable structural changes. In summary, this study determined that G43-C3-TEG is non-bactericidal and can selectively reduce the biofilm formation, by decreasing the production of EPS. This molecule will serve to functionalize surfaces of materials to be tested in future research.

## Introduction

Oral microbial infections leading to disease are prevalent in the general population. The most commonly observed oral pathologies are caries and periodontal disease, which can lead to serious systemic complications, such as endocarditis^1,2^. In the US, despite prevention campaigns emphasizing the importance of consistent, good-quality oral care, approximately 95% of the population aged 19–85 is affected by caries, whether newly formed or recurrent around restorations. In fact, dental composite restorations, despite being ubiquitous, only last about 10 years on average, with secondary decay and fracture leading the causes for replacement^3^. Past improvements in composites have focused on mechanical properties, mainly wear, fatigue and stress reduction. In adhesives, developments in self-etching techniques have also focused on mechanical aspects, such as bond strength^4^. Recently, more emphasis has been placed on how these materials interact with the oral environment, including bacteria and host-derived factors^5,6^. Oral bacteria establish a resilient biofilm, firmly attached to the surface, and protected from clearance by saliva and penetration by antimicrobial agents^7^. Biofilm formation and development is a result of early colonization of the protein-coated oral surfaces and coalescence of multi-species bacteria via the production of extracellular polymeric matrix (EPS), composed mostly of soluble and insoluble polysaccharides^8^. Effective antibacterial approaches must be multi-factorial, preferably combining antifouling, bactericide, and/or EPS-disrupting strategies (kill, disrupt and detach).

Current antibacterial approaches are not ideal because they rely on broad spectrum antibiotics. These are delivered via toothpaste/mouthwashes (chlorhexidine, triclosan) with only limited effect due to the transient nature of their contact with the bacteria, or can be tethered to the surface of restorative materials, such as polymerizable quaternary ammonium methacrylates^9^, and more recently, polymerizable imidazolium methacrylate compounds^10^. The main drawback is that those compounds indiscriminately affect pathogenic and commensal organisms via contact-kill mechanisms. That way, when the biofilm eventually gets re-established, bacteria such as *S. mutans* that induce dysbiosis are as likely to colonize oral surfaces as normal commensal bacteria such as *S. gordonii and S. sanguinis*^11,12^.

Ideally, antimicrobial therapy should follow a targeted approach, capable of selectively modulating oral ecology on the material surface to favor commensal species and deplete dysbiotic ones. In recent years, EPS-inhibiting strategies have been developed via the inactivation of glycosyltransferase (GTF), the cell surface and secreted proteins in *S. mutans* responsible for the polymerization of glucose into soluble and insoluble glucans^13^. These enzyme homologs are found in *S. sanguinis* (early colonizer, commensal), but are unique to cariogenic *S. mutans*, which makes them a viable target. Many small molecule compounds with high efficiency specifically against *S. mutans* GTFs have been designed, with nitro compounds showing the best selectivity and efficiency at inhibiting *S. mutans* biofilm formation when tested in multi-species biofilms^13^. So far, these small molecules have been tested in solution, which implies any delivery mechanism would require incorporation in oral rinses, for example, which have the significant disadvantage of depending on patient compliance. The incorporation of GTF-inhibiting monomers in the organic matrix of dental composites has the potential to significantly reduce dysbiotic bacterial colonization and maturation of the dental biofilm into calcified dental plaque, which is an important consideration for restoration loss. Instead, commensal biofilm formation is encouraged. These molecules can conceivably be made polymerizable through a variety of functional groups, including the methacrylates that are used in essentially all of the currently available commercial dental composites and adhesives. This is relevant because upon bacterial exposure, the material will impede the coalescence of biofilm on its surface. Since the anti-biofilm agent would be tethered to the surface, this eliminates common concerns with other delivery methods, such as mouthwashes: in the material proposed here, rather than a transient contact with bacteria, the GTF-targeting moiety is always available on the surface, and is independent of patient compliance or access of the anti-biofilm into the depths of interfacial gaps. These gaps have been demonstrated in composite restorations, and their contribution to new lesions have been investigated in a secondary caries model^14^.

Previous In silico screening of 500,000 drug-like small molecule compounds targeting defined G43 as a lead compound that drastically reduced bacterial virulence in the rat model of dental caries and demonstrated low micromolar affinity for GTF-B and more potent nanomolar affinity for GTF-C, and selectively inhibited *S. mutans* biofilms in single-species and dual-species biofilm. Docking analysis of this lead compound identified critical interactions of the ortho primary amide group of the compound with key active site residues of GTF-C. This observation was supported by the data obtained from an in vitro biofilm formation assay that used an analog lacking this functional group. However, the ability of the modified variation of G43 to covalently attach to materials and sustain its virulence against *S. mutans* remains to be determined^15^.

In this study, the overall objective was to design material surfaces capable of resisting biofilm attachment and maturation, by targeting enzymes involved in the synthesis of extracellular polymeric substance (EPS) with specificity to cariogenic bacteria. Specifically, G43 was modified with the addition of methoxy (OMe)/tetraethyleneglycol (TEG) substitutions in different positions to identify potential sites for functionalization, where the biological effect would not be affected. The small molecules were tested in solution for antibacterial effects, as well as for their GTF inhibitory potential. The test hypothesis is that the derivatization of the parent G43 molecule will affect its antibacterial and enzyme inhibitory potential, in a structure-dependent manner.

## Materials and methods

### Small-molecule compounds

**Figure 1:**
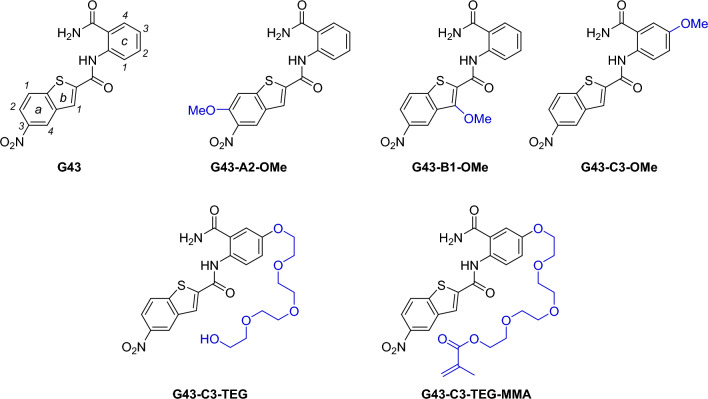
Structure of all compounds tested in this study.

G43 derivatives were synthesized, purified and characterized according to the procedures presented in detail in the supplemental information. Molecules were characterized with ^1^H- and ^13^C-NMR, and mid-IR. Structures related to the linker exploration are shown in Fig. 1. Additional derivatives without methoxy or tetraethylene glycol groups were synthesized for model validation purposes and did not undergo a full suite of testing. These compounds and related results can be found in the Supplemental Information.

The previously described G43 was used as the parent molecule based on previous results reported in the literature on its specificity against *S. mutans* biofilm formation^15^. This molecule was modified with the addition of methoxy (OMe)/tetraethyleneglycol (TEG) substitutions in different positions. The rationale for the derivatization of this parent molecule was the determination of the best synthetic region to attach extenders and eventually a polymerizing functionality to allow the incorporation of the anti-GTF activity to the surface of restorative materials. Stock solutions were prepared in dimethyl sulfoxide (DMSO) at 10 mM and the compounds were then tested at a concentration of 50 µM for their effects on bacterial viability and EPS production. This concentration is higher than previously reported^15^, mainly because a drop in effectiveness was expected with the substitutions proposed here. Acarbose was used as a control, given that this molecule was used to resolve the crystallographic structure of GTF-C, and therefore, interacts with the active site of the enzyme in computational models^16–18^. However, its effects as a true biofilm inhibitor are underexplored.

### Luciferase assay

*S. mutans* strain IdhRenGSm, derived from UA159 strain (here referred as wild type) was used in this study. The construction of *ldh* luciferase was described in a previous study^19^. Briefly, biofilm was grown by adding 1:500 dilution of the inoculum to Todd-Hewitt broth (THB) supplemented with 1% sucrose containing 50 μM of tested compounds in a 5% CO_2_ incubator at 37 °C, for 24 h, without agitation. The corresponding volume of DMSO was added as a control. Wells without bacteria received THB only and served as blanks. After the incubation time, 200 μL of fresh media was added per well and incubated at 37 °C for 1 h, under agitation. The bioluminescence activity was measured immediately after adding 2 μL of coelenterazine-h ethanol solution (Sigma Aldrich, St. Louis, MO, USA) to each well in a Spectramax iD3 Microplate reader (Molecular Devices LLC., San Jose, CA, USA). Data are presented in relative light units (RLU).

### Biofilm formation and inhibition assay

The inhibitory effect of tested compounds on bacterial biofilm formation was examined using *S. mutans* UA 159 and ∆GTF-B, ∆GTF-C and ∆GTF-BCD mutant strains, the construction for which has been previously reported^15^. Briefly, overnight cultures were sub-cultured into fresh THB, grown to an optical density at 600 nm (OD600) of 0.6 and 1:1000 diluted. The biofilm was grown in THB containing 1% sucrose and 50 μM of tested compounds. For control, the corresponding volume of DMSO was added. Groups consisting of only TEG and TEG with G43 were used as additional controls for the G43-C3-TEG group. Wells without bacteria received THB only and served as blanks. The inoculum was added in a 96-well microtiter plate and grown at 5% CO_2_ at 37 °C under agitation. Biofilm was grown for 24 h, the wells were gently washed three times with milli-q water, dried in an inverted position and stained with 0.1% crystal violet for 15 min. Images were taken and the crystal violet was solubilized in 200 μL of 30% acetic acid. The optical density at 562 nm was determined using a microplate reader.

### Determination of water-insoluble polysaccharides

Water-insoluble extracellular polysaccharides were quantified using the phenol–sulfuric method^20^. Briefly, overnight cultures of *S. mutans* UA159 and ∆GTF-BCD mutant strains (obtained following established methods^21^) were inoculated (1:250 diluted) in 24-well plate in 5% CO_2_ for 48 h at 37 °C in THB containing 50 μM of tested compounds or DMSO. Wells without bacteria received THB only and served as blanks. After incubation, the biofilm was washed two times with milli-q water to remove planktonic and non-adherent cells. Cells were resuspended in 1 mL of PBS solution by scraping the well bottom and by vigorous pipetting^22^. The suspension was transferred to a sterile 2-mL centrifuge tube and sonicated for 60s. An aliquot of 400 µL of sonicated biofilm suspension was vortexed and centrifuged at 10,000×*g* for 10 min at 4 °C. To the pellet, 400 µL of 1 M NaOH was added, tubes were vortexed, agitated for 15 min and centrifuged at 10,000×*g* for 10 min at 4 °C. The supernatant was transferred to another tube and 1.2 ml of cold 95% ethanol was added to each tube. The tubes containing cold ethanol were kept for 30 min at − 8 °C, centrifuged and the pellets were washed with cold 70% ethanol. The precipitated polysaccharides precipitated were resuspended in 200 µL of 1 M NaOH and total carbohydrate was estimated using glucose as standard^23^. To the sample, 200 µL of 5% phenol solution was added, followed by 1 mL of concentrated sulfuric acid added rapidly. The tubes were allowed to stand for 10 min, then vortexed and kept for 20 min in a water bath at 30°C. The absorbance was measured at 490 nm in a microplate reader.

### Polysaccharide linkage assay

The extracellular polymeric substances (EPS) was extracted from the biofilms, as described above, and then subjected to selective digestion to determine the connectivity within the polysaccharide molecules. Glucans produced by *S. mutans* through the Gtf enzymes are dextrose-based homopolymers with varying connectivity and branching that give rise to the material properties of the biofilms. For example, soluble glucan polymers are rich in α-1,6-dextran units, typically with α-1,6-dextran backbone polymer with α-1,3-mutan side-chains. Insoluble glucan polymers are rich in α-1,3-mutan linked sugars, typically α-1,3-mutan backbones with side-chains of α-1,6-dextrans. Using a well-known method^24^, optimized in our laboratory in the context of *Streptococcus mutans* EPS, we analyzed the relative abundance of 1,6- and 1,3-rich polysaccharides, extracted from biofilms treated with each of the G43 variants shown in Fig. 1. Briefly, the connectivity of dextrose homopolymers is determined after chemical digestion using GC–MS analysis, from which the ratio of the linkage types can be determined in the EPS within a biofilm. The glucan is first methylated, followed by depolymerization in trifluoroacetic acid and reduction with sodium borohydride. The resulting compound is then acetylated, to render methylated alditol acetates, which are then quantified using GC–MS. A thorough description of the analytical method will be published separately.

### CLSM analysis of biofilm

*S. mutans* biofilms were formed in a FluoroDish Cell Culture Dish by adding 1:500 dilution of the inoculum to THB media supplemented with 1% sucrose and containing 50 μM of lead compounds or DMSO in 5% CO_2_ incubator at 37 °C, for 24 h, static. The biofilm architecture and thickness exposed to G43 and G43-C3-TEG compounds were observed using a Carl Zeiss confocal laser scanning microscope (CLSM, Zeiss Axio Observer). One micromolar dextran-conjugated Cascade Blue (401/422 nm; Molecular Probes, Invitrogen) was added (1 µM) to overnight culture to label the EPS (overall). After incubation, the biofilms were gently washed with phosphate-buffered saline (PBS) three times to remove any nonadherent cells and then stained with 1 μM SYTO 9 green (Molecular Probes, Invitrogen) for 20 min. Biofilms were kept in PBS and examined by CLSM under × 20 magnification. Z sections were used to record the biofilm thickness and three random positions were analyzed in each image^22,25^. COMSTAT software was used to analyze image stacks and to calculate the biomass and covering percentage of EPS and bacterial cells^26,27^.

### Gel zymography

GTFs were extracted from liquid culture using dialysis in phenylmethylsulfonyl fluoride-containing PBS buffer, following previously published methods^28,29^. Supernatants of liquid culture of *S. mutans* were placed in 12–14,000 MW cut-off membranes (Spectrapor™, Repligen, Waltham, MA, USA) and proteins were extracted after overnight dialysis, then quantified using Bradford assay. Extracted and recombinant GTF-C and GTF-B (obtained with established methods^30^) were subjected to electrophoresis under non-reducing conditions in MOPS buffer. After electrophoresis, gels were washed twice with 2.5% Triton-X 100 (30 min each) under agitation, and incubated in 0.2 M sodium phosphate buffer (pH 6.8) containing 0.2% dextran, 5% sucrose and 50 µM of the different inhibitors at 37 °C for 48 h. Gels were then washed twice with DI water, and stained with Coomassie Blue R-250 0.1%, washed with 50% methanol until enough contrast was observed against the background by visual inspection. The gels were then imaged in transmission (ChemiDoc Imaging System, Bio-Rad Laboratories, Hercules, CA), and kept in 1% acetic acid.

### Enzyme kinetics

The inhibitory effect of G43-C3-TEG compound on GTFs activity was monitored using two complementary methods, since these enzymes play a role both in the hydrolysis of sucrose and in the polymerization of glucans^31^. To evaluate hydrolytic activity, and obtain *K*_*m*_ (Michaelis constant) and *K*_*cat*_ (catalytic constant), the chromogenic pNPG was used as substrate, as determined in a 96-well plate reader. Briefly, 200 µL of 50 mM phosphate buffer, pH 7.4, containing 0–25 mM pNPG, was supplemented with a concentration of 100 nM recombinant enzyme with or without inhibitor, preincubated at 30 °C for 30 min, and product generation was measured continuously for 60 min and read at optical density of 405 nm by using a microplate reader. *K*_*cat*_ were obtained at *V*_*max*_ (maximum enzyme velocity) and the rate of reaction when the enzyme is saturated with and apparent *K*_*m*_ as a measure of how easily the substrate associates with the enzyme. In turn, the glucan polymerizing activity for GTF-C was determined using Dynamic Light Scattering (DLS, DynaPro® Plate Reader, Wyatt) and sucrose as substrate. Changes in product size were collected considering the hydrodynamic radius (Rh) distribution intensity from 50 to 600 nm particles. GTF-C activity was measured in a 20 mM phosphate buffer, pH 6.5, at different sucrose (10, 15, 20, 50, and 100 mM) and compound concentrations at 30 °C, for 15 min in a 384-well plate. Data were analyzed by Dynamics V7 software. Kinetic parameters and the apparent inhibitory constant (*K*_*iapp*_) value was calculated by nonlinear fitting of experimental data derived from IC_50_^32^ using Grafit 5.0 software (Eritacus Software) and GraphPadPrism software (GraphPad Software, Inc., San Diego).

### Statistical analysis

All experiments performed included 6 biological replicates (except the CLMS analysis and kinetics, performed in triplicate). Data were tested for normality (Anderson–Darling) and homoscedasticity (Bartlett and Levene), and then analyzed with one-way ANOVA and Tukey’s test for multiple comparisons for all experimental tests, using Graph Pad Instat (GraphPad Software). The significance level was set at α = 0.05.

## Results

**Figure 2:**
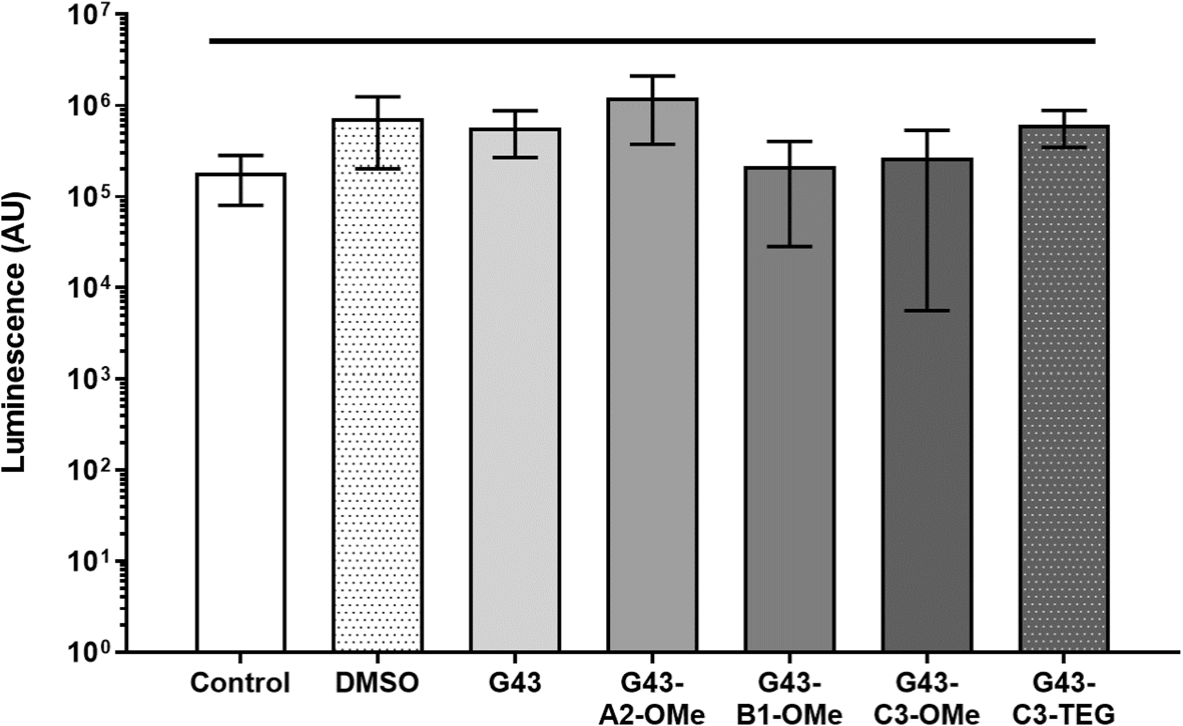
Luminescence obtained from renilla reporter S. mutans biofilms treated with the different G43 derivatives, compared with no treatment (media only control), DMSO, and known inhibitors All values were statistically similar (p = 0.0701).

Exposure of *S. mutans* luciferase biofilms to the G43-derivative compounds at 50 μM did not reduce the bacterial viability in relation to the control, regardless of the structure (Fig. 2). All groups were statistically similar (p = 0.0701).

**Figure 3:**
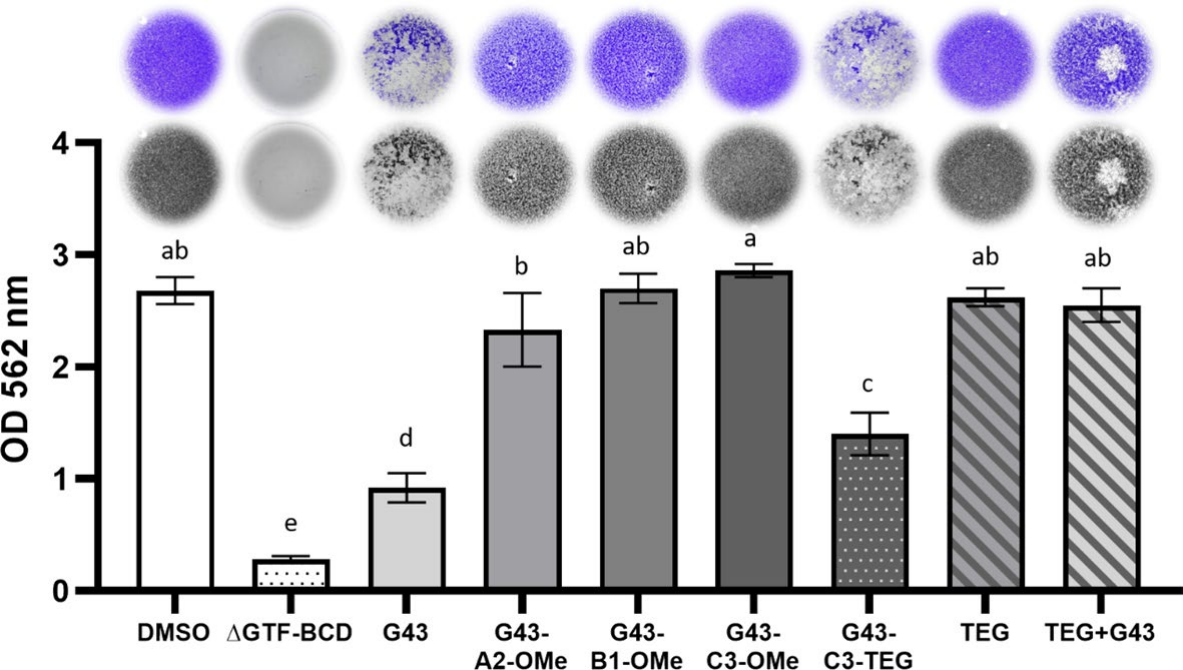
Crystal violet biomass assay with wild-type S. mutans biofilms treated with the different G43 derivatives, compared with DMSO (positive control). The GTF-BCD triple knockout strain (ΔGTF-BCD) served as the negative control. Values followed by the same letter are statistically similar (α = 0.05).

The crystal violet results (Fig. 3) show that, compared with the parent G43 molecule, biofilm inhibitory activity was lost for compounds modified with methoxy substitution, while the tetraethyleneglycol substitution (G43-C3-TEG) led to reduced biofilm biomass. The G43 molecule showed the greatest biomass reduction compared with the control (DMSO), followed by G43-C3-TEG and the G43-A2-OMe (p < 0.0001). The ∆GTF-BCD mutant showed the lowest biofilm formation, as expected. To decouple the potential surfactant effect of the TEG extension, this molecule was added to the culture medium either by itself or combined with the parent G43. The results demonstrate that while the G43-C3-TEG molecule somewhat recapitulated the inhibitory effect seen with the parent G43, TEG alone or combined with the parent G43 did not show biofilm inhibitory activity.

**Figure 4:**
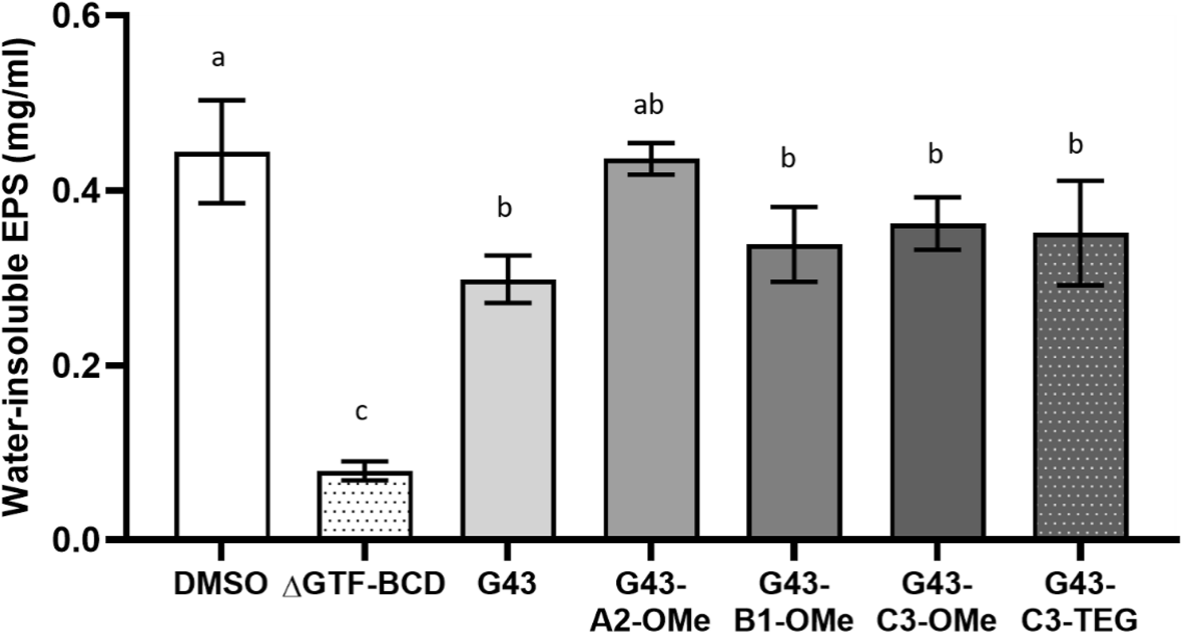
Water-insoluble polysaccharide extracted from wild-type S. mutans biofilms treated with the different G43 derivatives, compared with DMSO (positive control). The GTF-BCD triple knockout strain (ΔGTF-BCD) served as the negative control. Values followed by the same letter are statistically similar (α = 0.05).

Lead compounds were tested to quantify their impacts on water-insoluble EPS produced by *S. mutans* biofilm using phenol–sulfuric method (Fig. 4). The concentration of insoluble EPS was lower in the biofilm exposed to tested compounds than that found in the control group, except for G43-A2-OMe (p < 0.0001). This more closely demonstrates the effect of the different derivatives on EPS formation and confirm the crystal violet findings.

**Figure 5:**
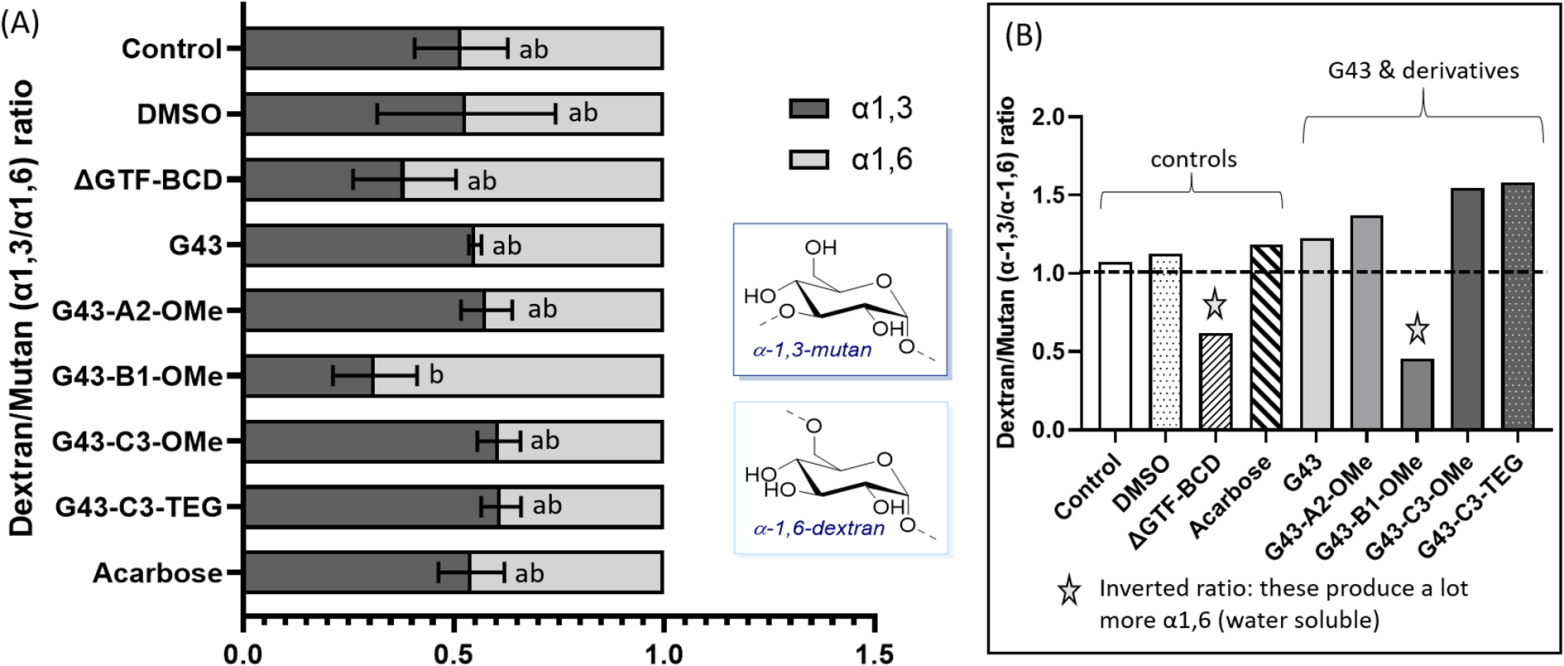
(**A**) Polysaccharide connectivity (1.3 vs. 1.6) derived from EPS extracted from wild-type *S. mutans* biofilms treated with the different G43 derivatives, compared with culture medium only (with and without 1% DMSO—positive control). The GTF-BCD triple knockout strain (ΔGTF-BCD) served as the negative control. Values followed by the same letter indicate statistically similar concentration of α1,3 linkages (α = 0.05). (**B**) The insert on the right shows that the ratio of 1,3 vs. 1,6 is close to 1 for the controls, and higher for all G43 derivatives, except for G43-B1-OMe, which produced a lot greater ratio of the much more soluble 1,6 glucan, similar to the triple knockout.

To further characterize different glucans, water soluble and insoluble glucans produced by S. mutans, we performed GC–MS analysis of glucans. Figure 5 describes the results for the linkage of the glucans produced in the presence of the different inhibitors, showing the ratio of α1,3 (insoluble) to α1,6 (soluble) glucans produced in these biofilms. While the two positive controls (culture medium with and without DMSO) produced equal amounts of α1,3 and α1,6 (plot on the left), for a ratio of about 1 (plot on the right), the G43 derivatives led to greater relative concentrations of the insoluble glucan (p = 0.0291), except for G43-B1-OMe, which produced relatively higher ratio of the much more soluble α1,6 glucan, similar to the triple knockout (it is important to note that the amount of glucans produced by G43-B1-OMe were much greater than the triple knockout, as shown in (Fig. 4).

**Figure 6:**
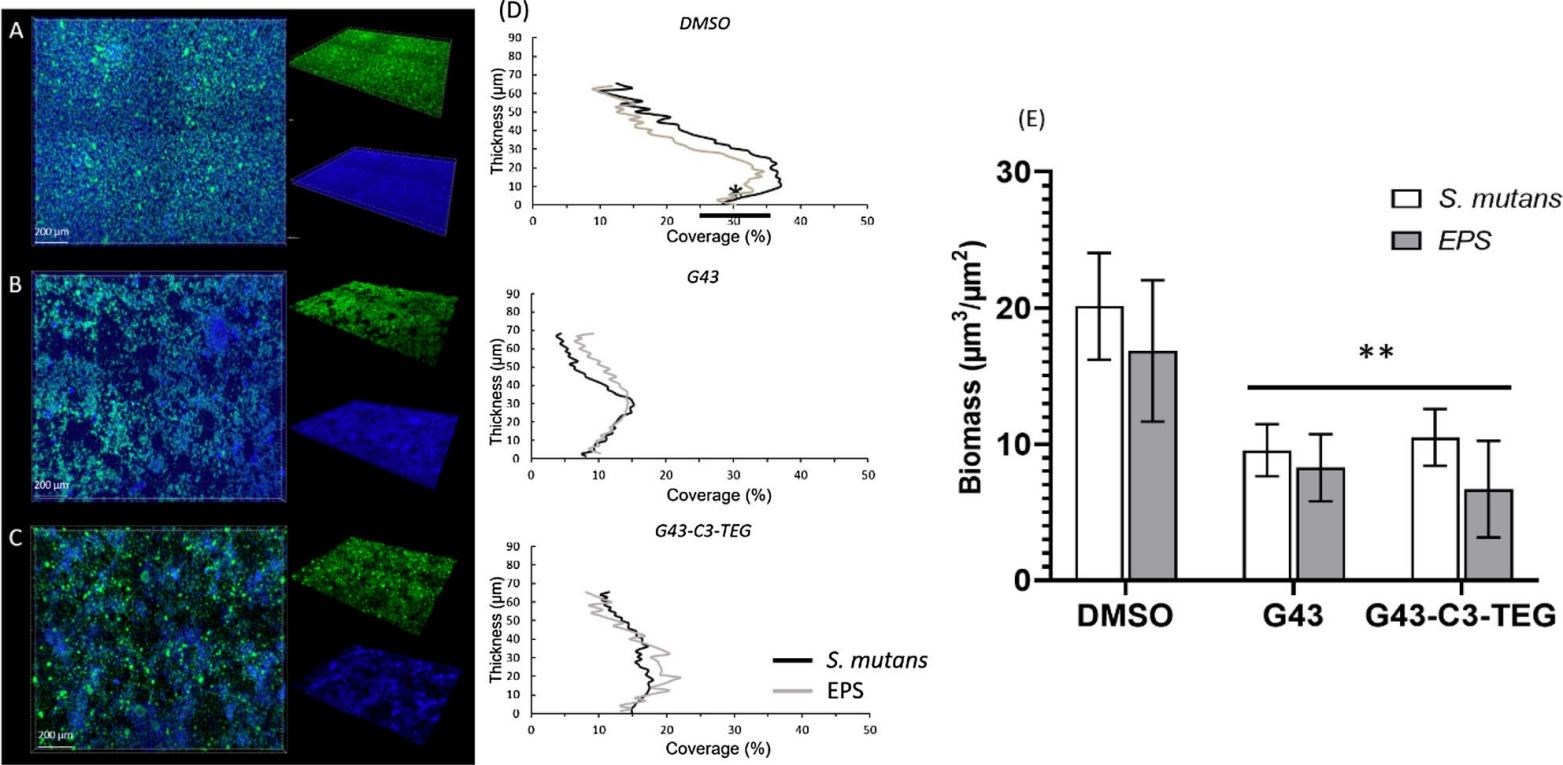
(**A–C**) Confocal light microscopy images of wild-type S. mutans biofilms treated with the parent G43 or the G43-C3-TEG derivative, compared with DMSO (positive control). (**D**) The graphs to the right of the respective representative image show the amount and thickness of EPS or bacteria coverage. (**E**) The bar graph on the insert shows the averages/standard deviation for the data derived from the line graphs (n = 5).

To further evaluate the underlying mechanisms of the antibiofilm activity of G43 and G43-C3-TEG compounds, the impact of these small molecules was investigated on biofilm architecture (Fig. 6). CLSM shows representative three-dimensional images of EPS (blue) and bacteria (green) in *S. mutans* biofilms. The images show disruption of the ability of *S. mutans* to synthesize the EPS matrix, with visible structural changes with minimal accumulation of polysaccharides on the FluoroDish surface. The distribution of bacteria and EPS were quantified and data demonstrated that the biofilm thickness and surface coverage were reduced with G43 and G43-C3-TEG (p = 0.0083 and p = 0.0165, respectively).

**Figure 7:**
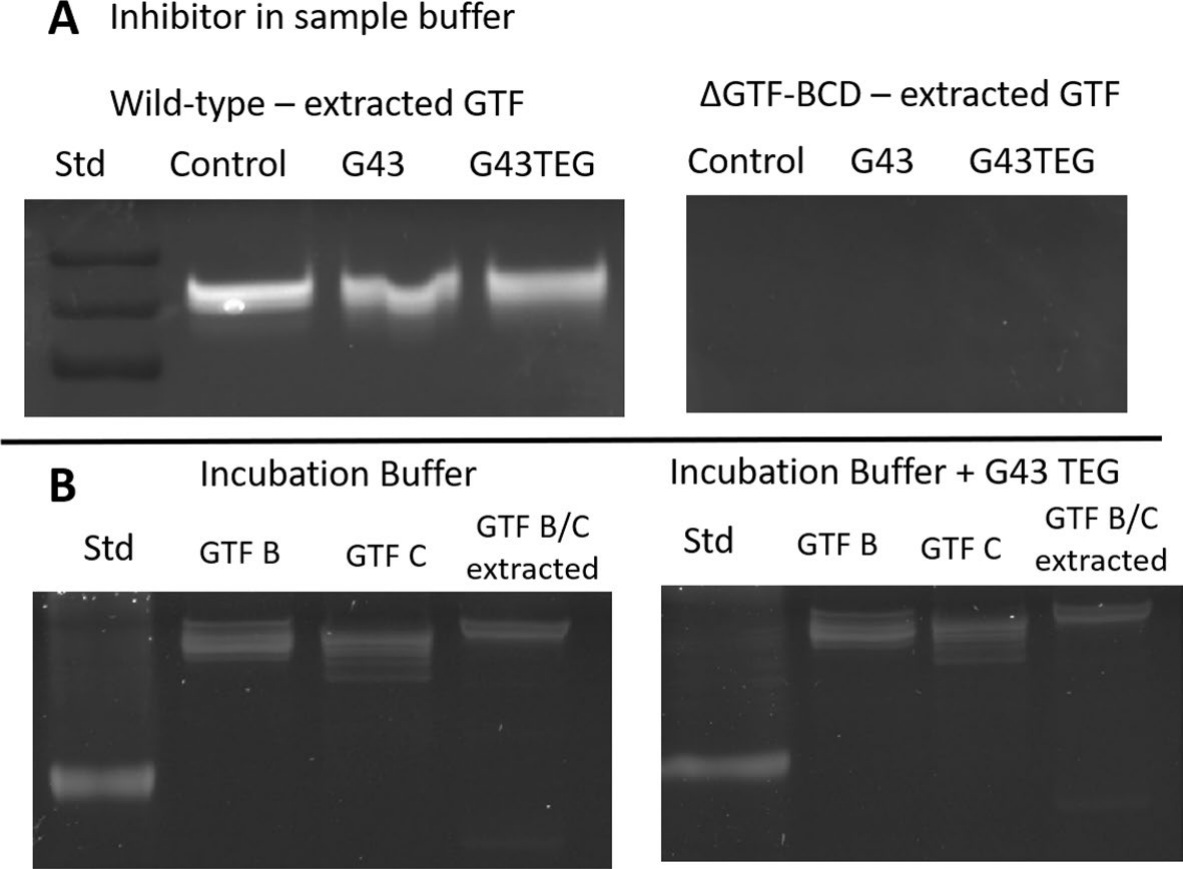
Gel zymography for activity of recombinant enzymes, or GTFs extracted from S. mutans biofilms (wild-type or triple GTF knockout—ΔGTF-BCD). In (**A**), the inhibitors were added to the sample buffer (incubated directly in solution with the enzyme). In (**B**) the inhibitor was added to the buffer into which the gels containing the enzymes. The inhibitors (parent G43 or the G43-C3-TEG derivative) were compared with DMSO (positive control). Sections of gels are separated by white space.

The gel zymography images are shown in Fig. 7. On panel A, the gels show decrease of activity of the extracted enzymes tested with the inhibitors added to the sample buffer. G43 and G43-C3-TEG led to a decrease of 21 and 10%, respectively (according to densitometry) in relation to the DMSO control for the wild-type biofilms, and as expected, there is no enzymatic activity in the triple knockout bacteria. On panel B, the gels show activity of the recombinant and extracted enzymes tested either with the incubation buffer (control) or with G43-C3-TEG added to the incubation buffer. In this case, all lanes came from the same gel, which was then cut in half. Each half was incubated separately in incubation buffer alone or containing the inhibitor, which then led to the control and inhibited lanes to be in different gels. Since all lanes were ran at the same time, the densitometries were calculated using images treated exactly the same way. The densitometry shows 19.5 and 11.5% inhibition for the recombinant GTF-B and C, respectively, and 27.1% of inhibition for the extracted enzymes (Fig. S6).

**Figure 8:**
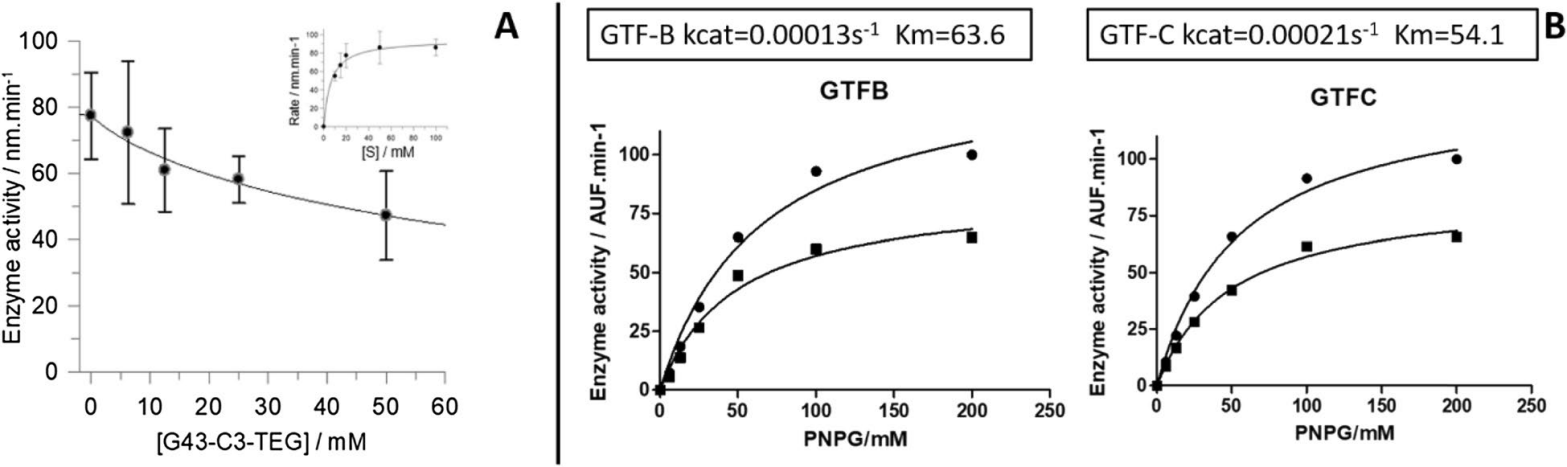
Enzyme activity for the recombinant enzymes assessed with (**A**) dynamic light scattering (GTF-C only) and (**B**) fluorescence assay (GTF-B and C treated with DMSO—positive control, circles, and G43-TEG, squares).

The G43-C3-TEG inhibitory mechanism was further investigated using DLS and fluorometric assays. Figure 8A shows the DLS kinetics (insert) and G43-C3-TEG dose–response inhibition profile for GTF-C activity. The affinity/dissociation constants obtained resulted from both sucrose hydrolysis and glucose polymerization reactions, and as such, this is considered an apparent constant. It is also important to note that the assay is limited at lower substrate concentrations, where the resulting product concentration is below the detection limit. At 20 mM sucrose, the half-maximum inhibitory constant (IC_50_) for G43-C3-TEG was calculated to be 23.5 µM, similar to what was reported for the parent G43 in previous studies (25 µM)^15^. The apparent inhibitory constant (*K*_*iapp*_) in the presence of G43-C3-TEG can be calculated from the IC_50_ values (*K*_*iapp*_ = IC50/2, except for non-competitive inhibitory behavior, when *K*_*iapp*_ = IC_50_^33^). Figure 8B shows the results for the kinetic assays using the chromogenic pNPG as substrate, which measures GTFs hydrolytic activity. The *K*_*m*_ for GTF-B and GTF-C (63.6 ± 13.9 and 54.1 ± 8.0 µM, respectively) were only slightly decreased in the presence of G43-C3-TEG (48.8 ± 11.0 and 49.0 ± 6.3 µM for GTF-B and GTF-C, respectively). The Lineweaver–Burk plot used to calculate *K*_*cat*_ (catalytic constant) and derive *K*_*m*_ is shown in Fig. S8.

## Discussion

In this study, the parent G43 molecule was systematically derivatized with methoxy groups, a method commonly used in the pharmaceutical industry during the development of targeted drugs^34^, to determine possible points for functionalization that would not affect enzymatic activity of the glycosyltransferase enzymes. Additionally, the methoxy groups were used to simulate a more flexible, hydrophilic extender (tetraethylene glycol) would be needed to allow for later functionalization with a methacrylate moiety. This was determined in a pilot study with in silico docking (Figs. S9, S10)—showing that the methacrylate would in theory still be accessible for radical polymerization. From our docking study and others^15^, the nitro group was suggested to “dock” to the active site of the enzyme leaving the “C” aromatic ring (Fig. 1) point out of the enzyme active site and making it the most likely position to successfully add a linker. While other positions around the G43 molecule may merit further investigation with a longer linker, a tetraethylene glycol was added at the C3-position (G43-C3-TEG) and later methacrylated (supplemental information). In summary, the main goal is to identify potential disruptors of biofilm development that can be used to modify restorative surfaces, and attempt to translate the effects obtained in solution for the parent G43 molecule^15^.

The luciferase assay demonstrated that neither G43 nor any of its derivatives affect biofilm viability (Fig. 2), which was expected since its antibiofilm mechanism of action does not include cell wall disruption. This test measures the bacterial metabolic activity by quantifying the amount of a fluorescent product, giving an overall assessment of viability for all bacteria present on the surface of a sample, but it does not differentiate between biofilm morphologies. For that reason, in addition to the luciferase assay, the biofilms were also stained and imaged, and the biomass quantified, using the crystal violet assay. Both the biomass and the biofilm morphology were significantly affected by the presence of the parent G43 (as previously demonstrated^15^), and at least two of its derivatives: G43-C3-TEG and G43-A2-OMe (Fig. 3). The parent G43 had the most significant inhibition, with only the triple knockout bacteria showing a lower level of biofilm formation. This indicates that the sum of the mass of bacteria and extra-polysaccharide substance (EPS) was reduced for those two compounds. Since the luciferase assay showed no change in bacterial viability, as already mentioned, this indicates those two compounds were able to significantly reduce the amount of EPS formed. For all other derivatives tested, the presence of the methoxy substitution, regardless of the location, reduced or impeded the anti-biofilm effect. The fact that the G43 with different substitutions in the C3 position (G43-C3-TEG and G43-C3-OMe) presented different effects on the biomass quantification was not expected. As mentioned earlier, drug derivatization is used to identify impediments to molecular docking on the catalytic domain^35^, so we expected that both the methoxy and the TEG groups would behave similarly. One potential explanation for the difference is the improved solubility of G43 by the addition of the more hydrophilic TEG group. The difference in log P is not that significant (1.563 and 2.723 for TEG and methoxy derivatives, respectively), but the effect in solubility in the DMSO-containing culture medium was noticeable at concentrations higher than 50 µM. In fact, G43 by itself (log P = 2.717) was very difficult to dissolve in the culture medium, with agglomeration at concentrations higher than 10 µM. It is important to note that in this study, the DMSO concentration was limited to 5%, which convolutes direct comparisons with the solubility of the parent G43 in previous studies^15^. In addition, the G43 synthesized here had a purity greater than 95%, which also helps explain differences in solubility compared to other studies. It needs to be pointed out that, in spite of the lower solubility, the parent G43 had greater inhibitory effect than G43-C3-TEG, which may confirm that the MIC for this compound is lower than 50 µM, and/or that agglomeration effects might play a role^36^.

To test the hypothesis that the effect of TEG in the inhibitory potential of G43 was linked to increased solubility compared with the methoxy-modified counterparts, the parent G43 was added to solution along with untethered TEG and tested using the crystal violet assay (Fig. 3). Under those conditions, the inhibitory effect from G43 disappeared, indicating that TEG was not merely acting as a surfactant in G43-C3-TEG, but rather that the effect on tuning the log P of the parent molecule was significant. In addition, when added as individual compound, TEG might have led to a change in the Gibbs free energy of mixing of the system as a whole, leading some of the G43 to precipitate, and therefore, reducing the effective concentration available in solution. Another possibility to explain the greater inhibitory potential of G43-C3-TEG compared with the methoxy analog, albeit less likely, is that docking may have been facilitated by the longer and more flexible TEG chain compared to the short hydrophobic methoxy group^37^. Finally, it is possible that the TEG moiety has presented allosteric interaction with different sites on the enzyme, decreasing its activity via a non-active site-specific mechanism^38^. Preliminary computational simulations suggest greater interaction of G43-C3-TEG with the enzyme (Fig. S10), demonstrating that modifications in the C3 can provide a potentially tunable site for functionalization for this molecule in the future. By itself, TEG does not present any inhibitory effect, as confirmed by the crystal violet assay.

It is also important to note that true inhibitors of GTF-C activity are not well-established, complicating the selection of a robust positive control for these assays. Acarbose is commonly used in computational simulations of enzymatic docking, and has been shown to work as an inhibitor of direct GTF activity^18,39–41^, typically at concentrations in the millimolar range. One of these studies has shown partial enzymatic inhibition with 10 mM^40^. However, in this present study, acarbose did not affect the biofilm formation as measured with the crystal violet assay and had very inconsistent results when added at 50 µM or 20 mM, a 4000-fold greater concentration than the 50 µM used for the G43 compounds (Fig. S3). This is not completely unexpected, and in fact, the need for higher concentrations usually points to aggregate-based inhibition^42^, which is non-specific. This reiterates the need to identify more efficient inhibitors of this EPS-producing enzyme. Additionally, the success of screening by in silico docking may be greatly improved with solved crystal structures of Gtf enzymes using more specific, tighter binding substrates.

The luciferase and crystal violet assays were used as screening tools to identify successful targets, so other than the two that showed statistically significant effects (G43-C3-TEG and G43-A2-OMe), G43-B1-OMe and G43-C3-OMe were subjected to a more in-depth analysis of their effect on the amount of insoluble polysaccharide produced by biofilms. The assay used here does not quantitate the total amount of polysaccharide produced by the bacteria, but instead, determines the fraction that is sequestered in the biofilm, which plays the most crucial role in its coalescence (Fig. 4). The triple knockout bacteria were used as the negative control, producing a negligible amount of insoluble polysaccharides. All G43 derivatives tested, except for one of the methoxy-containing groups, led to statistically lower polysaccharide concentration compared with the DMSO control. It is interesting to note that, in spite of not affecting the overall biomass in the crystal violet assay, G43-B1-OMe and G43-C3-OMe led to the same reduction in insoluble polysaccharide production as G43-C3-TEG. Moreover, the G43 parent molecule had statistically similar reduction to these three compounds. This apparent discrepancy may be at least partially explained by differences in the sensitivity of the two assays. The crystal violet assay measures the total biomass formed, and also provides qualitative data on the morphology of the biofilms. Even though the total absorbance was the same, the biofilms for G43-B1-OMe and G43-C3-OMe appeared more disorganized compared with the DMSO control, albeit much less so compared with G43 and G43-C3-TEG. This points to the role played by the insoluble components of EPS on the hierarchical organization of biofilms, as demonstrated for *S. mutans*, and other bacterial species^43^. The insoluble glucans are the major structural component in the EPS and represent 10–20% of its dry weight. They play a critical role affecting the virulence of the biofilm by enhancing adherence of microorganisms and acting as a diffusion barrier in the biofilm^44,45^. In fact, the ratio of insoluble-soluble glucans produced with each inhibitor varied greatly. While the controls produced equal amounts of α-1,6 (soluble) and α-1,3 (insoluble) glucans, the G43 derivatives decreased the relative concentration of the soluble compound (albeit not statistically), while also reducing the overall concentration of polysaccharides produced (Fig. 5). Interestingly, the one exception was found for the G43 molecule modified at the B1 position, which showed a much lower α-1,3/α-1,6 ratio (increased relative production of the more soluble glucan), similar to the triple knockout. However, the B1 derivative led to the production of a three to four-fold greater quantity of insoluble polysaccharide compared with the triple knockout (Fig. 4), which may indicate that this particular variation may interfere with glucan-binding domains in the enzyme rather than the specific binding site^46,47^. Unfortunately, it was not synthetically possible to further functionalize this derivative with the TEG extender, as already mentioned, which we hypothesize would have improved solubility issues and allowed for methacrylate functionalization, so this compound was not included in the subsequent tests.

Further analysis using confocal microscopy involved only the two compounds with the most prominent biofilm reduction: G43 and G43-C3-TEG (Fig. 6). The staining used in this method allowed us to separately quantify the bacterial load and the insoluble EPS production—the biofilms were grown directly in the Fluorodish used in the microscope and washed to eliminate soluble compounds prior to imaging. These images highlight that both the bacterial load and the amount of EPS produced are lower compared with the control, for either inhibitor. Since the luciferase assay demonstrated that G43 and its derivatives are not bactericidal, the reduction in bacterial load here is explained by reduced superficial attachment due to a deficiency in binding biomolecules. This is demonstrated by the significant reduction of EPS formation, which generally agrees with the polysaccharide assay results. In terms of ranking, the confocal images agree more closely with the crystal violet assay, which is expected since both methods measure the total biomass. The EPS quantification here is lower for G43-C3-TEG compared with G43, in contrast with the polysaccharide assay, which is also expected since the latter only measures one component of the EPS.

The kinetic assay performed using dynamic light scattering was only possible for G43-C3-TEG due to solubility issues with the parent molecule, as already discussed (Fig. 8). G43 showed signs of clustering at concentrations as low as 25 µM, which precluded readings in the DLS. This means that for all other tests, where the solubility issue was not noticeable, the working concentration of G43 may actually have been lower than 50 µM. This has two potential implications: 1. The actual inhibitory concentration is much lower than 50 µM, which agrees with previously published results^15^, or 2. The mechanism of inhibition actually relies on non-specific aggregation, which is leading cause of artifacts in early drug discovery^42,48^. For reasons not yet completely understood, aggregates inhibit enzymes nonspecifically at relatively high concentrations (micromolar range) in aqueous solution^42^. Potential mechanisms involve a net increase in proton accessibility upon aggregate binding, indicating denaturation, combined with an increased tendency for proteolysis of the enzyme-aggregate complex^42^. Even though the aggregate-derived mechanism still results in reduced biofilm formation and stability, as shown here with the crystal violet and confocal images, this defeats the purpose of targeting specifically GTF-C enzymes in *S. mutans*. In other words, G43 might also inhibit polysaccharide production in other Streptococci species, which could then lead to the elimination of symbiotic bacteria from the oral biofilm. One previous study demonstrated that G43 does not inhibit GTF activity of the commensal *S. sobrinus*^15^, but its effects in more complex multi-species biofilms is unknown. This is yet another reason to produce derivatives with tunable solubility, and increase the range of applications. Indeed, G43-C3-TEG was soluble at the concentrations used here. The kinetics of recombinant GTF-C was investigated with DLS, which measures the size and amount of polysaccharide as it is being produced by the enzyme. Slower formation of shorter chains indicates that the inhibitor is effectively precluding enzyme activity. For this enzyme, the substrate (sucrose) concentration at which the activity approached the maximum rate was determined to be 20 mM, and therefore, this substrate concentration was used to build the graph for enzymatic kinetics in the presence of G43-C3-TEG. The resulting half-maximum inhibitory constant (IC_50_) was calculated to be 23.5 µM, which is similar to previously data published for the parent G43^15^, indicating that this derivative was as efficient at inhibiting the enzymatic activity as the parent molecule—with the caveat that the parent molecule was not tested in this study due to solubility limitations, as already mentioned. Furthermore, the kinetic data indicates that G43-C3-TEG negatively affects both *K*_*m*_ (slightly, already discussed) and *V*_*max*_ (39 and 36% for GTF-B and -C, respectively, as calculated with Grafit software from the kinetics curves). The observed catalytic constant for those enzymes was 0.00013 and 0.00021 s^−1^, respectively. This is consistent with an uncompetitive mode of action, suggesting that the inhibitor might not be acting directly at the active site. Combining the DLS and pNPG data, it can be inferred that G43-C3-TEG modulates both the hydrolytic (sucrose hydrolysis) and glucan polymerization activity of GTFs. Finally, it is important to note that the pNPG assay is a simple screening method that measures only the initial hydrolytic activity of glucosyltransferases, representing a single function of a complex enzyme. Truly elucidating the mode of action of this G43 variant would require much more comprehensive biochemical studies, including how the compound affects GTF gene expression. Since the inhibitory concentration was in the micromolar range, and the mechanism was not conclusively specific to the active site of the GTFs tested, other molecules might need to be developed for material surface functionalization, where an effective inhibitor would likely need to have an IC_50_ in the low micromolar or even nanomolar range. Judicious localization of such moieties can be tailored by using strategies such as decorated polymer brushes, also to be further investigated in future studies.

## Conclusions

In summary, the present results demonstrate that G43 is a platform for Gtf enzyme inhibition with a myriad of unexpected results developing from its derivatives including a change in biofilm morphology, composition, and retention of biofilm inhibition with an extended TEG chain. The reasons for these effects are undoubtedly multifactorial in the complex biological system, however it is likely that solubility of the G43 derivative and delicate Gtf enzyme interactions are the drivers of the diverse results observed.

These results also demonstrate that it is possible to extend a linker chain to G43 and retain biological activity. However, given the relatively high concentration needed to exert anti-biofilm effects, future studies will focus on designing inhibitors with higher specificity and to determine structure–property relationships in surfaces functionalized with them, tested against multi-species biofilms. Ultimately, the goal of this work is to create covalently tethered molecules for antifouling surfaces with specificity against caries-forming bacteria, to be used either as composites or adhesives. These findings demonstrate the first proof of principle towards those ends.

## Supplementary Information

Supplementary Information.

## Data Availability

Other than what is presented in this manuscript and on its Supplementary Information file, the datasets used and/or analyzed during the current study available from the corresponding author on reasonable request.

## References

[1] Petersen PE, Bourgeois D, Ogawa H, Estupinan-Day S, Ndiaye C (2005). The global burden of oral diseases and risks to oral health. Bull. World Health Organ..

[2] Pihlstrom BL, Michalowicz BS, Johnson NW (2005). Periodontal diseases. The Lancet.

[3] Opdam NJM, van de Sande FH, Bronkhorst E, Cenci MS, Bottenberg P, Pallesen U, Gaengler P, Lindberg A, Huysmans MCDNJM, van Dijken JW (2014). Longevity of posterior composite restorations: A systematic review and meta-analysis. J. Dent. Res..

[4] Maciel Pires P, Dávila-Sánchez A, Faus-Matoses V, Nuñez Martí JM, Lo Muzio L, Sauro S (2022). Bonding performance and ultramorphology of the resin-dentine interface of contemporary universal adhesives. Clin. Oral Investig..

[5] Speranza Zabeu G, Candia Scaffa PM, Ciccone Giacomini M, de Mattos Pimenta Vidal C, Tjäderhane L, Wang L (2022). Gelatinolytic activity after dentin pretreatment with dimethyl sulfoxide (DMSO) combined to dental bonding systems: Perspectives for biological responses. J. Mech. Behav. Biomed. Mater..

[6] Münchow EA, Da Silva AF, Piva E, Cuevas-Suárez CE, De Albuquerque MTP, Pinal R, Gregory RL, Breschi L, Bottino MC (2020). Development of an antibacterial and anti-metalloproteinase dental adhesive for long-lasting resin composite restorations. J. Mater. Chem. B.

[7] Verkaik MJ, Busscher HJ, Jager D, Slomp AM, Abbas F, Van Der Mei HC (2011). Efficacy of natural antimicrobials in toothpaste formulations against oral biofilms in vitro. J. Dent..

[8] de Farias AL, Arbeláez MIA, Meneguin AB, Barud HDS, Brighenti FL (2022). Mucoadhesive controlled-release formulations containing morin for the control of oral biofilms. Biofouling.

[9] Balhaddad AA, Mokeem LS, Weir MD, Xu H, Melo MAS (2021). Sustained antibacterial effect and wear behavior of quaternary ammonium contact-killing dental polymers after one-year of hydrolytic degradation. Appl. Sci..

[10] Hwang G, Koltisko B, Jin X, Koo H (2017). Nonleachable imidazolium-incorporated composite for disruption of bacterial clustering, exopolysaccharide-matrix assembly, and enhanced biofilm removal. ACS Appl. Mater. Interfaces.

[11] Kreth J, Merritt J, Pfeifer CS, Khajotia S, Ferracane JL (2020). Interaction between the oral microbiome and dental composite biomaterials: Where we are and where we should go. J. Dent. Res..

[12] Wang C, van der Mei HC, Busscher HJ, Ren Y (2020). *Streptococcus mutans* adhesion force sensing in multi-species oral biofilms. NPJ Biofilms Microb..

[13] Nijampatnam B, Ahirwar P, Pukkanasut P, Womack H, Casals L, Zhang H, Cai X, Michalek SM, Wu H, Velu SE (2021). Discovery of potent inhibitors of *Streptococcus mutans* biofilm with antivirulence activity. ACS Med. Chem. Lett..

[14] de Lucena FS, Lewis SH, Fugolin APP, Furuse AY, Ferracane JL, Pfeifer CS (2022). Triacrylamide-based adhesives stabilize bonds in physiologic conditions. J. Dent. Res..

[15] Zhang Q, Nijampatnam B, Hua Z, Nguyen T, Zou J, Cai X, Michalek SM, Velu SE, Wu H (2017). Structure-based discovery of small molecule inhibitors of cariogenic virulence. Sci. Rep..

[16] Hartman AM, Jumde VR, Elgaher WAM, Te Poele EM, Dijkhuizen L, Hirsch AKH (2021). Potential dental biofilm inhibitors: Dynamic combinatorial chemistry affords sugar-based molecules that target bacterial glucosyltransferase. ChemMedChem.

[17] Ren Z, Chen L, Li J, Li Y (2016). Inhibition of *Streptococcus mutans* polysaccharide synthesis by molecules targeting glycosyltransferase activity. J. Oral Microbiol..

[18] Newbrun E, Hoover CI, Walker GJ (1983). Inhibition by acarbose, nojirimycin and 1-deoxynojirimycin of glucosyltransferase produced by oral Streptococci. Arch. Oral Biol..

[19] Merritt J, Senpuku H, Kreth J (2016). Let there be bioluminescence: Development of a biophotonic imaging platform for in situ analyses of oral biofilms in animal models. Environ. Microbiol..

[20] Dubois M, Gilles KA, Hamilton JK, Rebers PA, Smith F (1956). Colorimetric method for determination of sugars and related substances. Anal. Chem..

[21] Zhu F, Zhang H, Yang T, Haslam SM, Dell A, Wu H (2016). Engineering and dissecting the glycosylation pathway of a streptococcal serine-rich repeat adhesin. J. Biol. Chem..

[22] Chen W, Liang JP, He Z, Jiang W (2016). Preliminary study on total protein extraction methods from enterococcus faecalis biofilm. Genet. Mol. Res..

[23] Aires CP, Del Bel Cury AA, Tenuta LMA, Klein MI, Koo H, Duarte S, Cury JA (2008). Effect of starch and sucrose on dental biofilm formation and on root dentine demineralization. Caries Res..

[24] Doares SH, Albersheim P, Darvill AG (1991). An improved method for the preparation of standards for glycosyl-linkage analysis of complex carbohydrates. Carbohyd. Res..

[25] Rainey K, Michalek SM, Wen ZT, Wu H (2019). Glycosyltransferase-mediated biofilm matrix dynamics and virulence of *Streptococcus mutans*. Appl. Environ. Microbiol..

[26] Heydorn A, Nielsen AT, Hentzer M, Sternberg C, Givskov M, Ersboll BK, Molin S (2000). Quantification of biofilm structures by the novel computer program COMSTAT. Microbiology.

[27] Vorregaard, M. *Comstat2—A Modern 3D Image Analysis Environment for Biofilms* (2008).

[28] Mattos-Graner RO, Napimoga MH, Fukushima K, Duncan MJ, Smith DJ (2004). Comparative analysis of Gtf isozyme production and diversity in isolates of *Streptococcus mutans* with different biofilm growth phenotypes. J. Clin. Microbiol..

[29] Mattos-Graner RO, Smith DJ, King WF, Mayer MPA (2000). Wafer-insoluble glucan synthesis by mutans streptococcal strains correlates with caries incidence in 12- to 30-month-old children. J. Dent. Res..

[30] Zhang Q, Ma Q, Wang Y, Wu H, Zou J (2021). Molecular mechanisms of inhibiting glucosyltransferases for biofilm formation in *Streptococcus mutans*. Int. J. Oral Sci..

[31] McGhee JR, Chia J-S, Yang C-S, Chen J-Y (1998). Functional analyses of a conserved region in glucosyltransferases of *Streptococcus mutans*. Infect. Immun..

[32] Yung-Chi C, Prusoff WH (1973). Relationship between the inhibition constant (KI) and the concentration of inhibitor which causes 50 percent inhibition (I50) of an enzymatic reaction. Biochem. Pharmacol..

[33] Haupt LJ, Kazmi F, Ogilvie BW, Buckley DB, Smith BD, Leatherman S, Paris B, Parkinson O, Parkinson A (2015). The reliability of estimating K_i_ values for direct, reversible inhibition of cytochrome P450 enzymes from corresponding IC_50_ values: A retrospective analysis of 343 experiments. Drug Metab. Dispos..

[34] Liu X, Zhang M, Tian Y, Liu R, Wang Y, Guo F, Gong Y, Yan M (2022). Development, characterization, and investigation of in vivo targeted delivery efficacy of luteolin-loaded, eudragit S100-coated mPEG-PLGA nanoparticles. AAPS PharmSciTech.

[35] Zhu J-S, Stiers KM, Winter SM, Garcia AD, Versini AF, Beamer LJ, Jakeman DL (2019). Synthesis, derivatization, and structural analysis of phosphorylated mono-, di-, and trifluorinated d-gluco-heptuloses by glucokinase: Tunable phosphoglucomutase inhibition. ACS Omega.

[36] Zhai R, Chen G, Liu G, Huang X, Xu X, Li L, Zhang Y, Wang J, Jin M, Xu D, AbdEl-Aty AM (2022). Enzyme inhibition methods based on Au nanomaterials for rapid detection of organophosphorus pesticides in agricultural and environmental samples: A review. J. Adv. Res..

[37] Erlanson DA, Lam JW, Wiesmann C, Luong TN, Simmons RL, DeLano WL, Choong IC, Burdett MT, Flanagan WM, Lee D, Gordon EM, O'Brien T (2003). In situ assembly of enzyme inhibitors using extended tethering. Nat. Biotechnol..

[38] Pelaz B, del Pino P, Maffre P, Hartmann R, Gallego M, Rivera-Fernández S, de la Fuente JM, Nienhaus GU, Parak WJ (2015). Surface functionalization of nanoparticles with polyethylene glycol: Effects on protein adsorption and cellular uptake. ACS Nano.

[39] Tsunoda T, Samadi A, Burade S, Mahmud T (2022). Complete biosynthetic pathway to the antidiabetic drug acarbose. Nat. Commun..

[40] Wright WG, Thelwell C, Svensson B, Russell RRB (2002). Inhibition of catalytic and glucan-binding activities of a streptococcal GTF forming insoluble glucans. Caries Res..

[41] Gou SH, Liu J, He M, Qiang Y, Ni JM (2016). Quantification and bio-assay of α-glucosidase inhibitors from the roots of *Glycyrrhiza uralensis* Fisch. Nat. Prod. Res..

[42] Coan KED, Maltby DA, Burlingame AL, Shoichet BK (2009). Promiscuous aggregate-based inhibitors promote enzyme unfolding. J. Med. Chem..

[43] Wangpaiboon K, Padungros P, Nakapong S, Charoenwongpaiboon T, Rejzek M, Field RA, Pichyangkura R (2018). An α-1,6-and α-1,3-linked glucan produced by *Leuconostoc citreum* ABK-1 alternansucrase with nanoparticle and film-forming properties. Sci. Rep..

[44] Bowen WH, Koo H (2011). Biology of streptococcus mutans-derived glucosyltransferases: Role in extracellular matrix formation of cariogenic biofilms. Caries Res..

[45] Hata S, Mayanagi H (2003). Acid diffusion through extracellular polysaccharides produced by various mutants of *Streptococcus mutans*. Arch. Oral Biol..

[46] Takashima Y, Fujita K, Ardin AC, Nagayama K, Nomura R, Nakano K, Matsumoto-Nakano M (2015). Characterization of the dextran-binding domain in the glucan-binding protein C of *Streptococcus mutans*. J. Appl. Microbiol..

[47] Banas JA, Vickerman MM (2003). Glucan-binding proteins of the oral streptococci. Crit. Rev. Oral Biol. Med..

[48] Torosyan H, Shoichet BK (2019). Protein stability effects in aggregate-based enzyme inhibition. J. Med. Chem..

