# Supplementary material for: The potential use of glycosyl-transferase inhibitors for targeted reduction of S. mutans biofilms in dental materials
Source: Sci Rep. 2023 Jul 23;13:11889. doi: 10.1038/s41598-023-39125-2 (PMC10363545; doi:10.1038/s41598-023-39125-2)
Supplement: Supplementary file 1 — Supplementary Information. [file 41598_2023_39125_MOESM1_ESM.docx]

Supplemental Information for:

**The potential use of glycosyl-transferase inhibitors for targeted reduction of *S. mutans* biofilms in dental materials**

Polliana Mendes Candia Scaffa^1^; Alexander Kendall^1^; Marcelo Yudi Icimoto^1,2^; Ana Paula Piovezan Fugolin^1^; Matthew G. Logan^1^; Andre G. DeVito-Moraes^1^, Steven H. Lewis^1^; Hua Zhang^1^; Hui Wu^1^; Carmem Pfeifer^1^


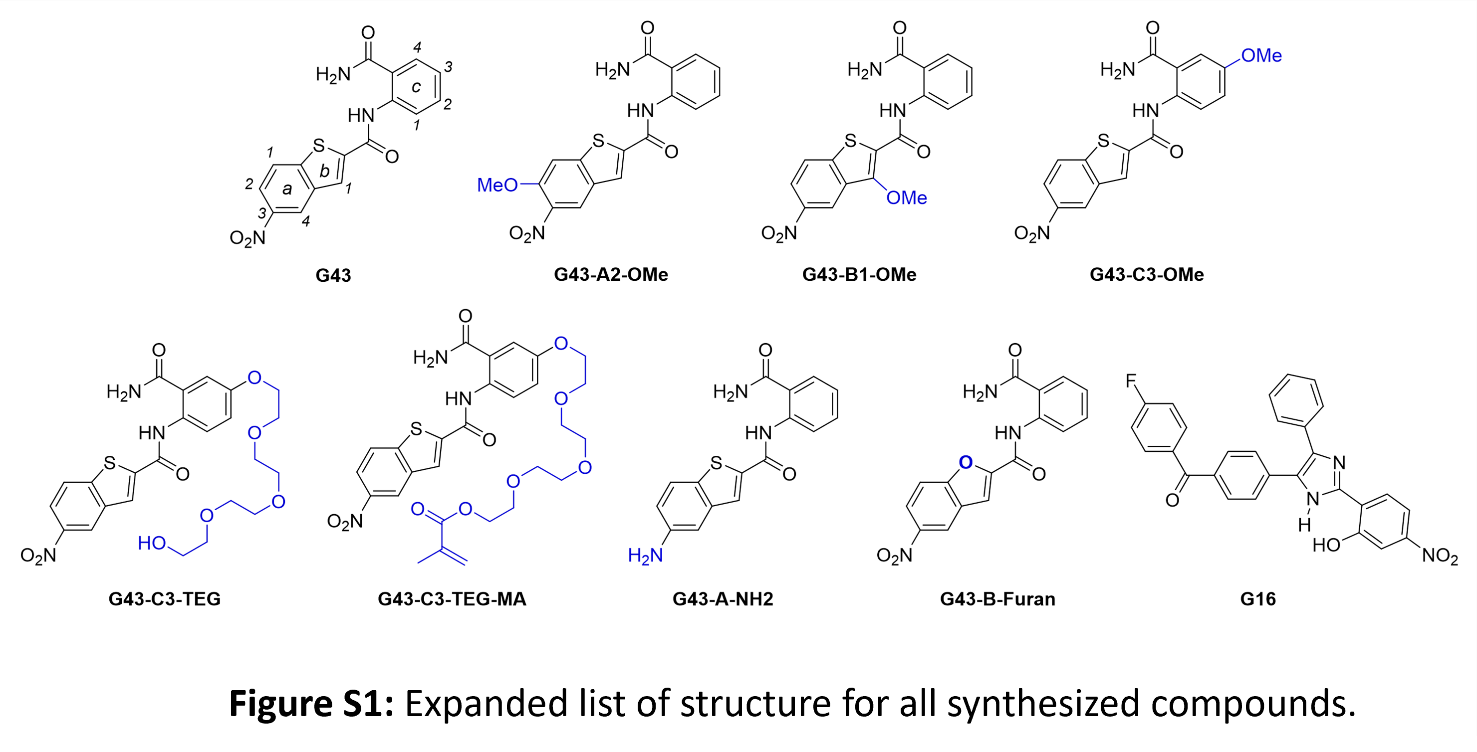


# Expanded Figures

## *Structures*


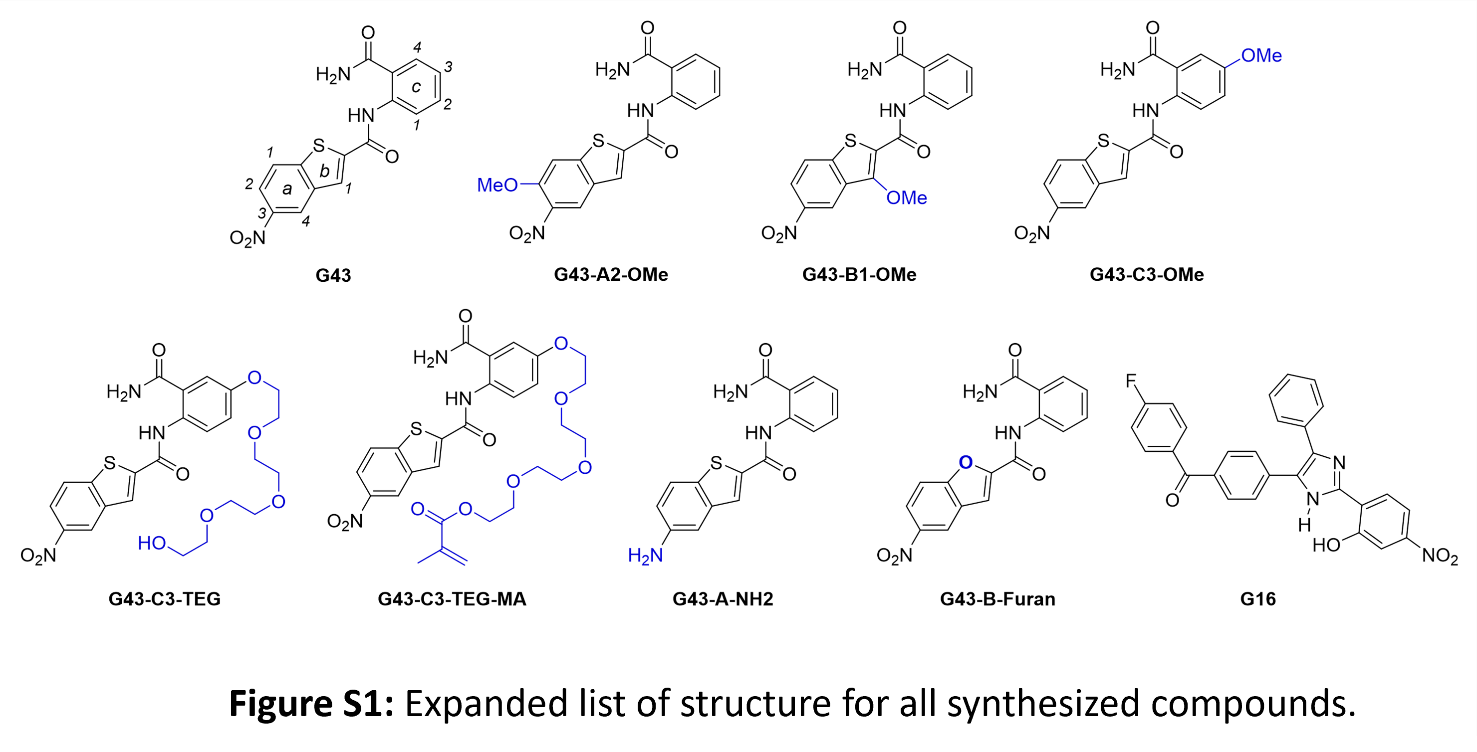


**Figure S1:** Expanded list of structure for all synthesized compounds.

## *Solubility Considerations*

The parent G43 compound was observed to have solubility lower than the recently reported value of 29.3 µM [1]. Dissolving G43 in DMSO followed by the slow addition of water or buffer solutions was used to try and increase the solubility of G43 to levels near the reported biofilm IC_50_ (16.7 µM). At 1%, 5%, and 10% DMSO, 12.5 µM solutions appeared to be fully dissolved but after letting sit for 72 hours, G43 precipitate had formed, suggesting that DMSO only allows for a metastable, supersaturated solution and not true solubility. Although no precipitate had formed after 24 hours, signs of aggregation were present in samples with a as low as 12.5 µM (Figure S2). Due to this, G43 solutions were prepared fresh and used as soon as possible for all analyses.


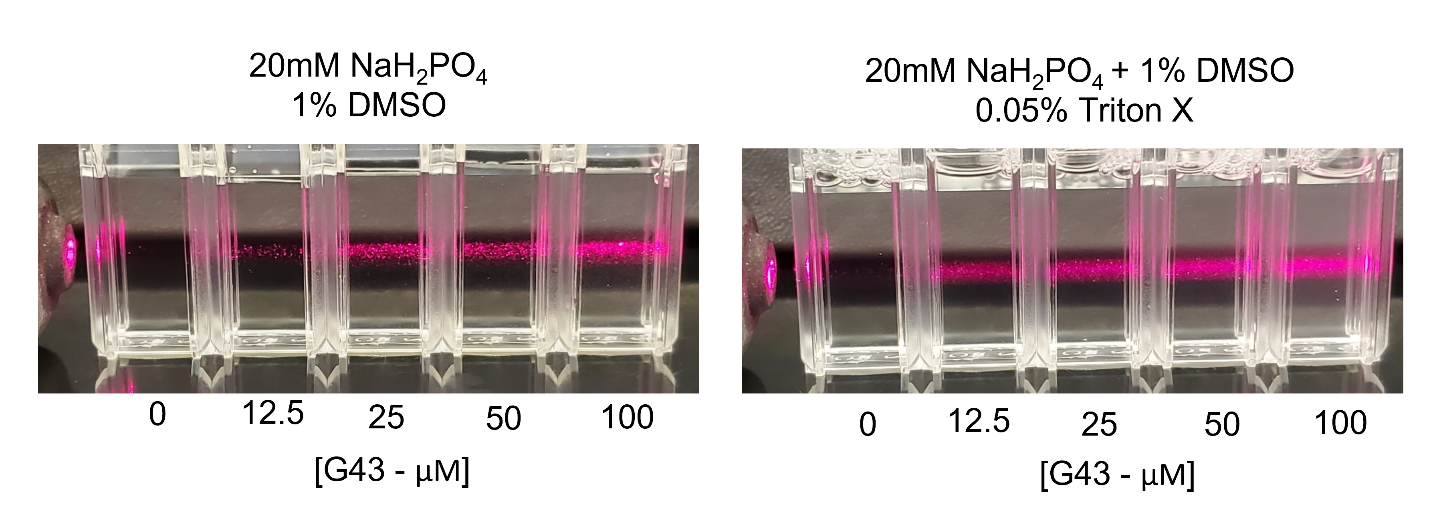


**Figure S2:** Light scattering of handheld laser pointer as evidence of aggregation of G43 at concentrations above 12.5 µM . A buffered solution was used in place of DI water to simulate conditions more similar to growth media. In an attempt to prevent aggregation, DMSO (1%) alone and with Triton (0.05%) were added.

## *Luminescence*


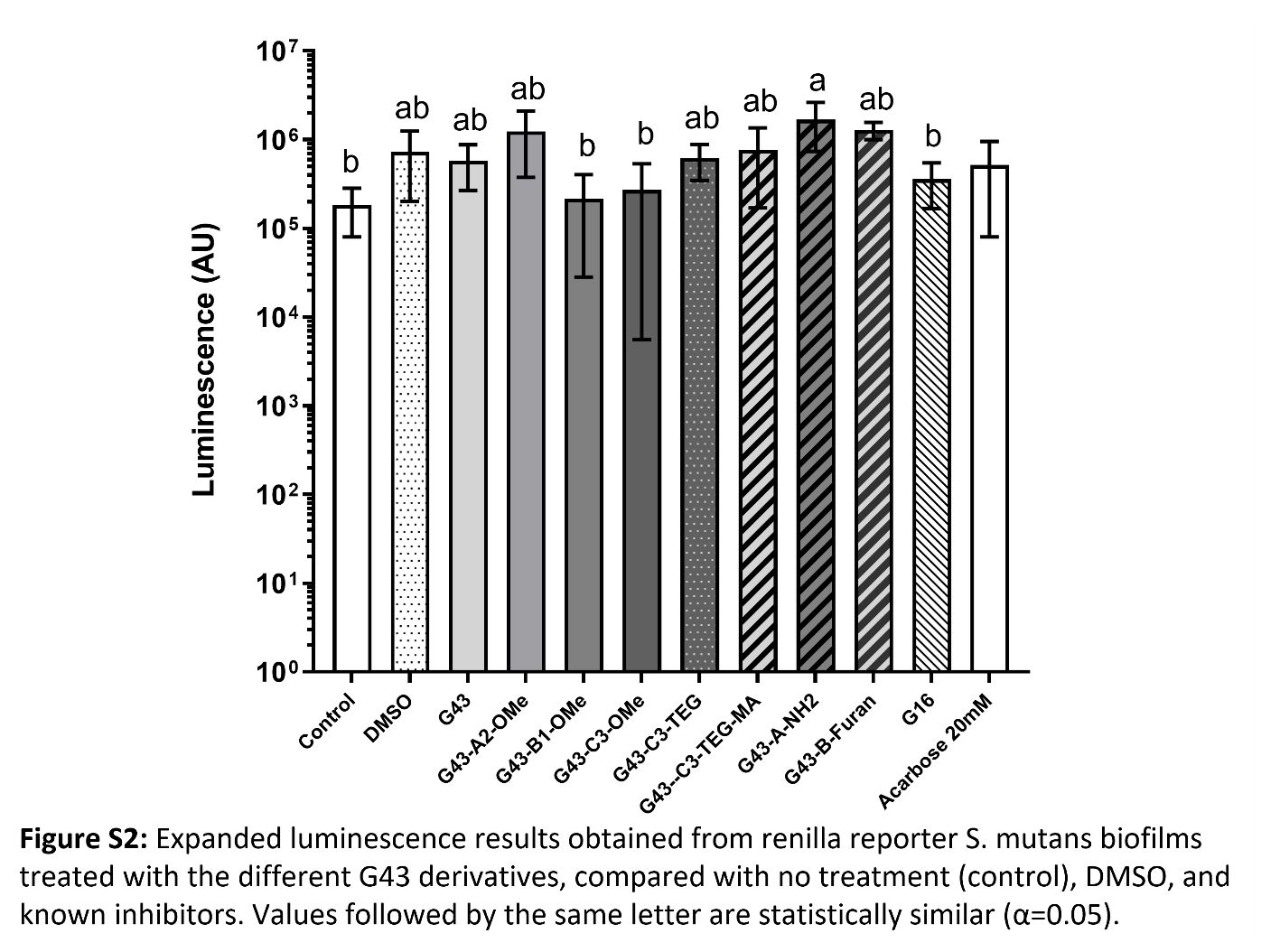


**Figure S3:** Expanded luminescence results obtained from renilla reporter S. mutans biofilms treated with the different G43 derivatives, compared with no treatment (control), DMSO, and known inhibitors. Values followed by the same letter are statistically similar (α=0.05).

## *Biofilm Assay*


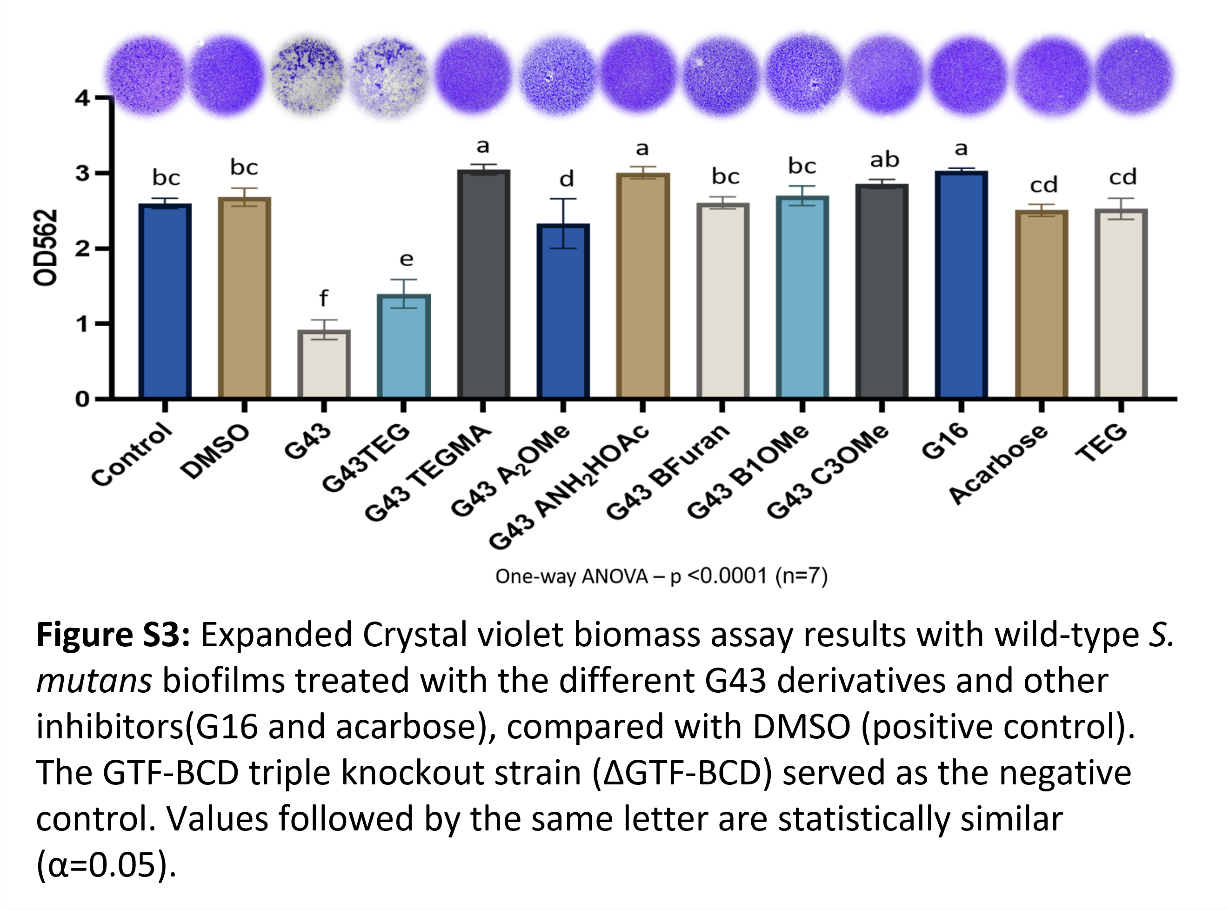


**Figure S4:** Expanded Crystal violet biomass assay results with wild-type *S. mutans* biofilms treated with the G43 derivatives, other inhibitors (G16 and acarbose), and controls (DMSO and TEG). Values followed by the same letter are statistically similar (α=0.05).

## *Connectivity Assay*


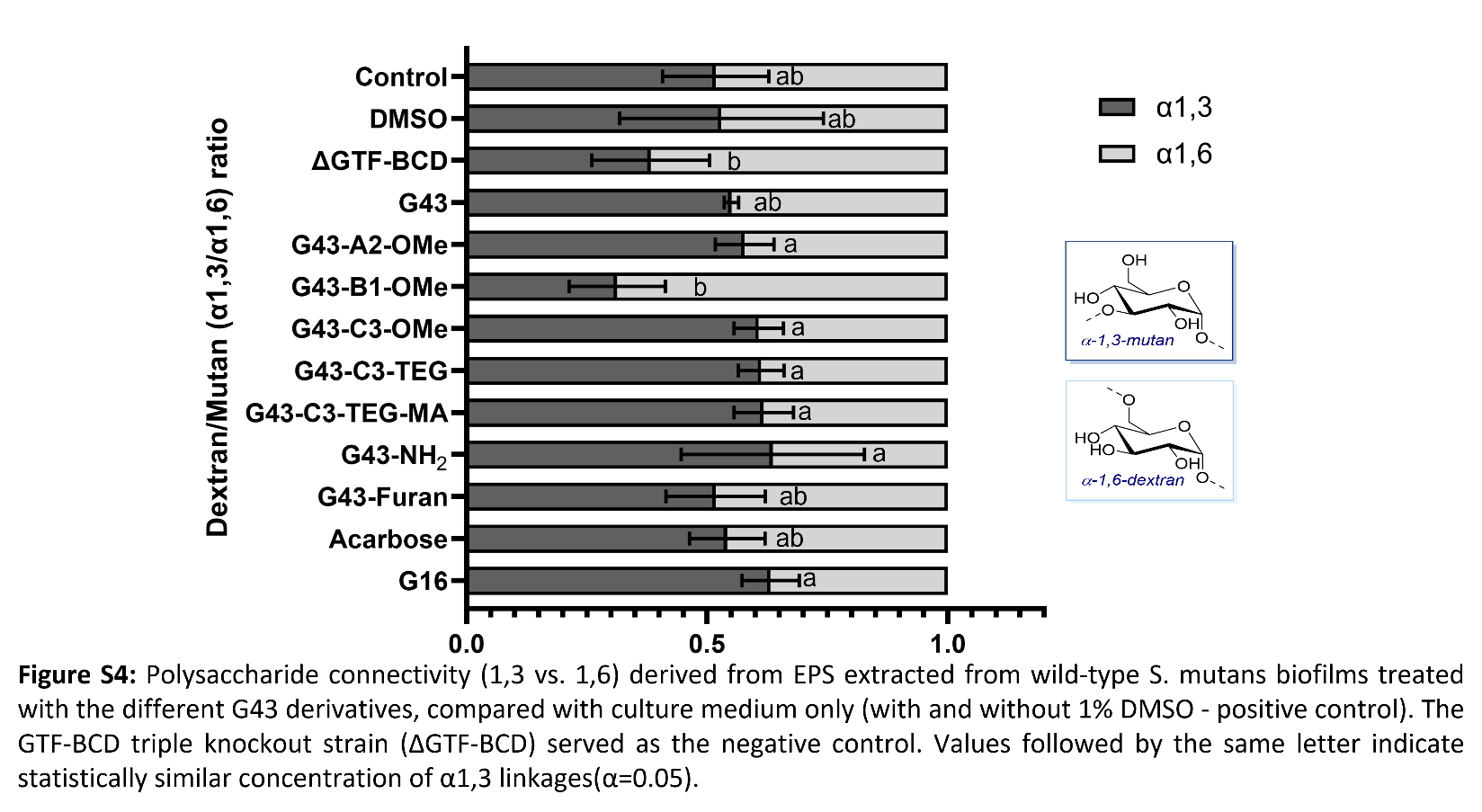


**Figure S5:** Expanded results of polysaccharide connectivity (1,3 vs. 1,6) derived from EPS extracted from wild-type S. mutans biofilms treated with the different G43 derivatives, compared with culture medium only (with and without 1% DMSO - positive control). The GTF-BCD triple knockout strain (ΔGTF-BCD) served as the negative control. Values followed by the same letter indicate statistically similar concentration of α1,3 linkages(α=0.05).

## *Densitometry Analysis*

**Figure S6:** Densitometry analysis on the zymography gels for GTF-B and C treated with DMSO (control) or the inhibitor (G43-C3-TEG).

## *pNPG Assay*


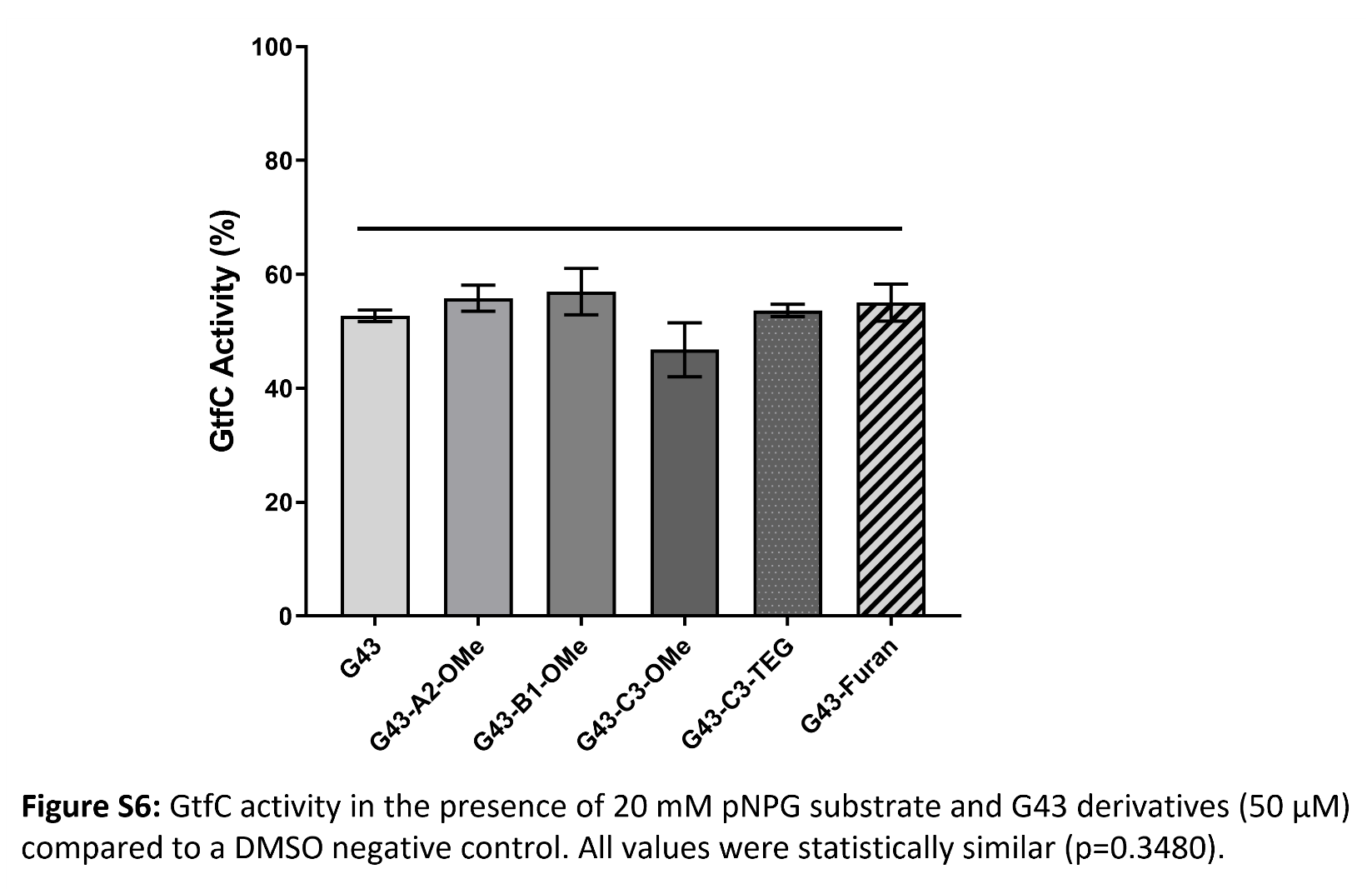


**Figure S7:** Percent GtfC activity in the presence of 20 mM pNPG substrate and G43 derivatives (50 μM) relative to a DMSO negative control. All derivatives led to statistically similar enzyme activity reduction, around 60% (p=0.3480).

## *Lineweaver-Burk Plots*


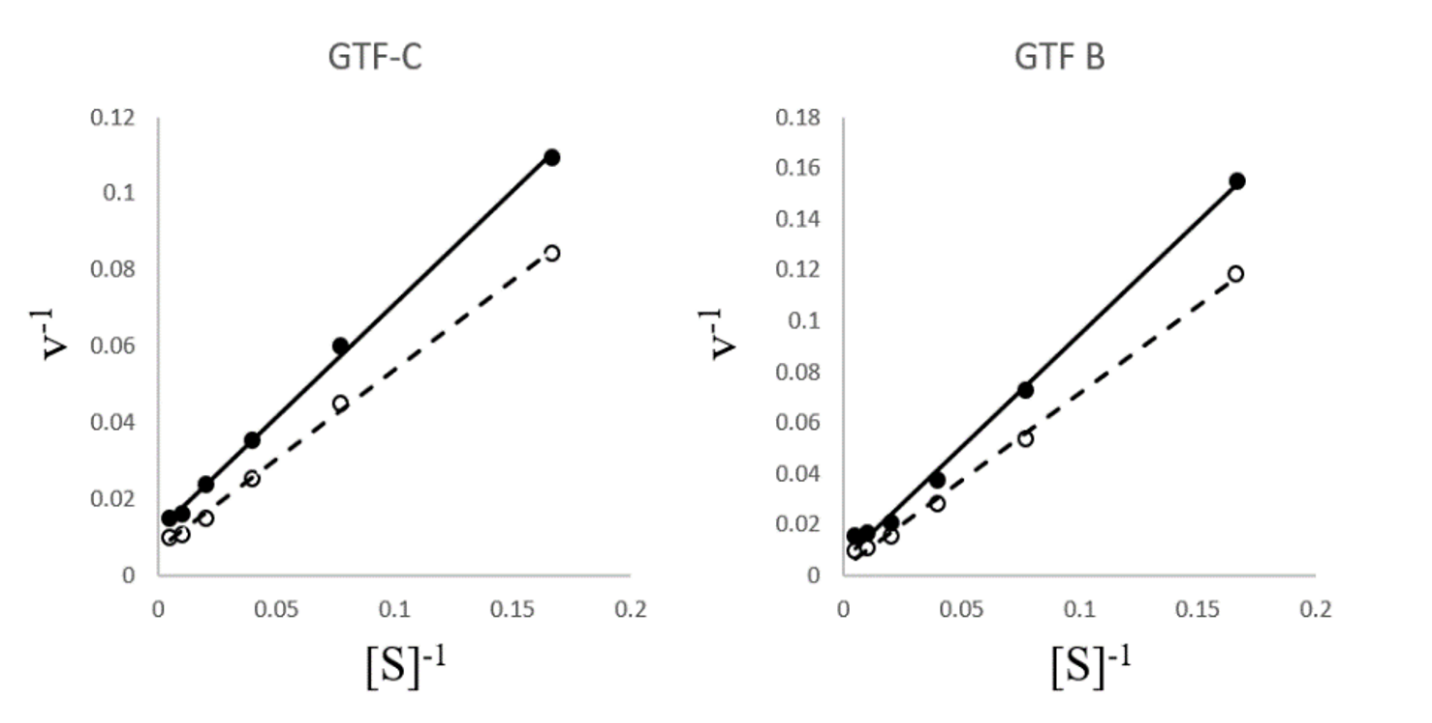


**Figure S8:** Double-reciprocal graphical plot (Lineweaver-Burk) of inhibition of GTF-B and C by G43-C3-TEG, suggesting non-competitive inhibition.

*
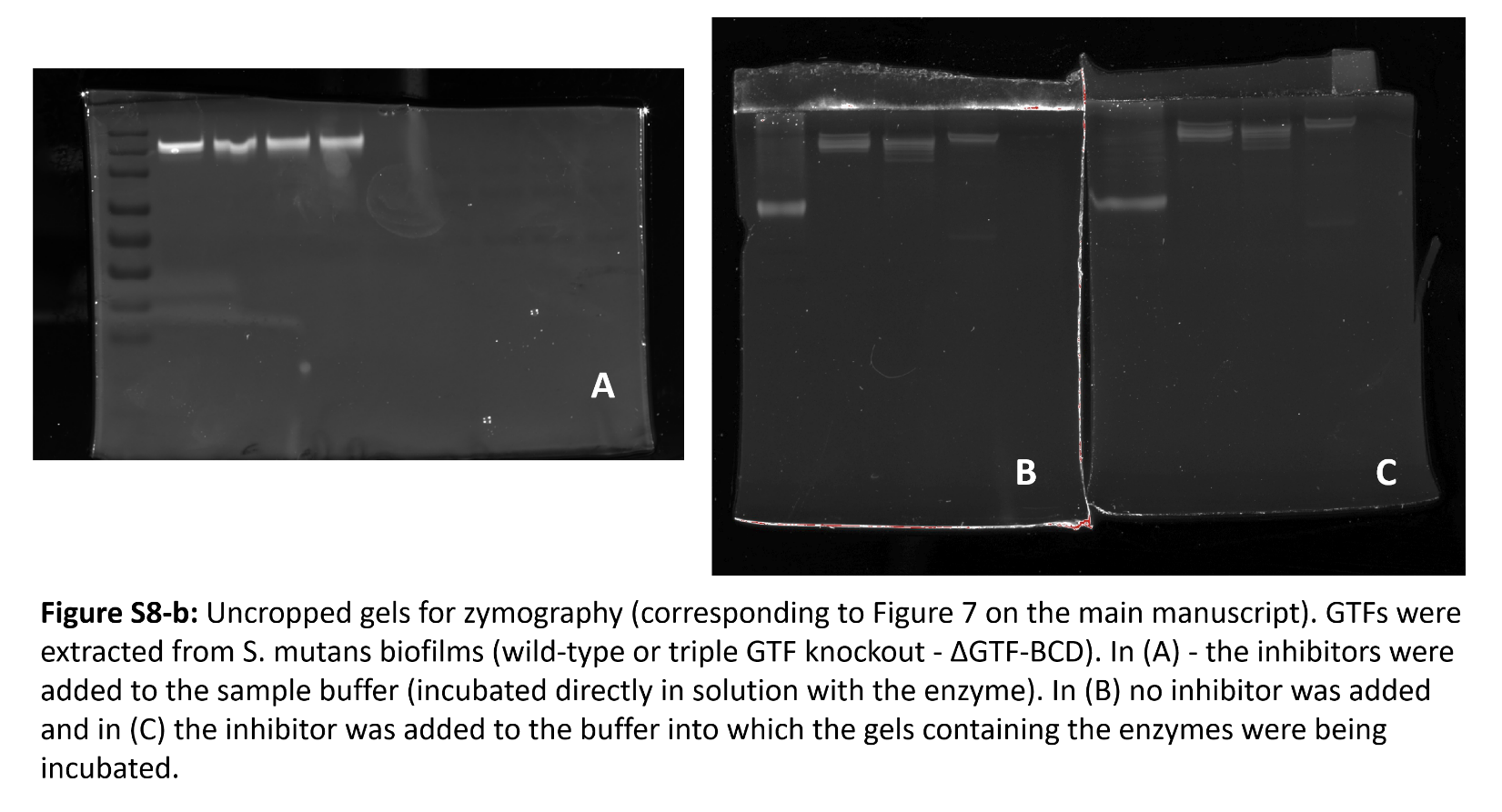
*

# *In silico* Docking

## *In silico* docking screening

The GtfC structure (PDB: 3AIC) preparation included removal of water molecules and polymers that are used during crystalization of the protein, addition of hydrogen and short minimization to eliminate bad contacts. The docking protocol involves two main steps, one is active-site grid definition and another is conformational modeling of small molecules in the active-site grid. In the OHSU medicinal chemistry core, we use Schrodinger GLIDE (grid-based ligand docking with energetics) package for small-molecule docking. GLIDE predicts the binding pose of ligands in several steps. First, the active-site shape and properties are represented on a grid by different fields. The active-site is defined based on the 20Å region surrounding the ligand (here acarbose) occupying the catalytic domain, each grid voxels contain van der Waals Radius and charge information corresponding to the amino acids in the active-site. A set of initial ligand conformations is generated, and the lowest energy conformer is retained for the next step. Next, the best pose conformation energy is minimized at the receptor environment with the OPLS-AA force field (22) in conjunction with the distance-dependent dielectric model. Finally, Monte Carlo simulation is conducted between the three to six lowest energy poses of the previous step to explore nearby torsional minima. To score the ligand pose, Glidescore, an expanded version of ChemScore (23), is used. In general, the lowest (in negative scale) energy conformer is considered to be the optimal binder of the protein target.


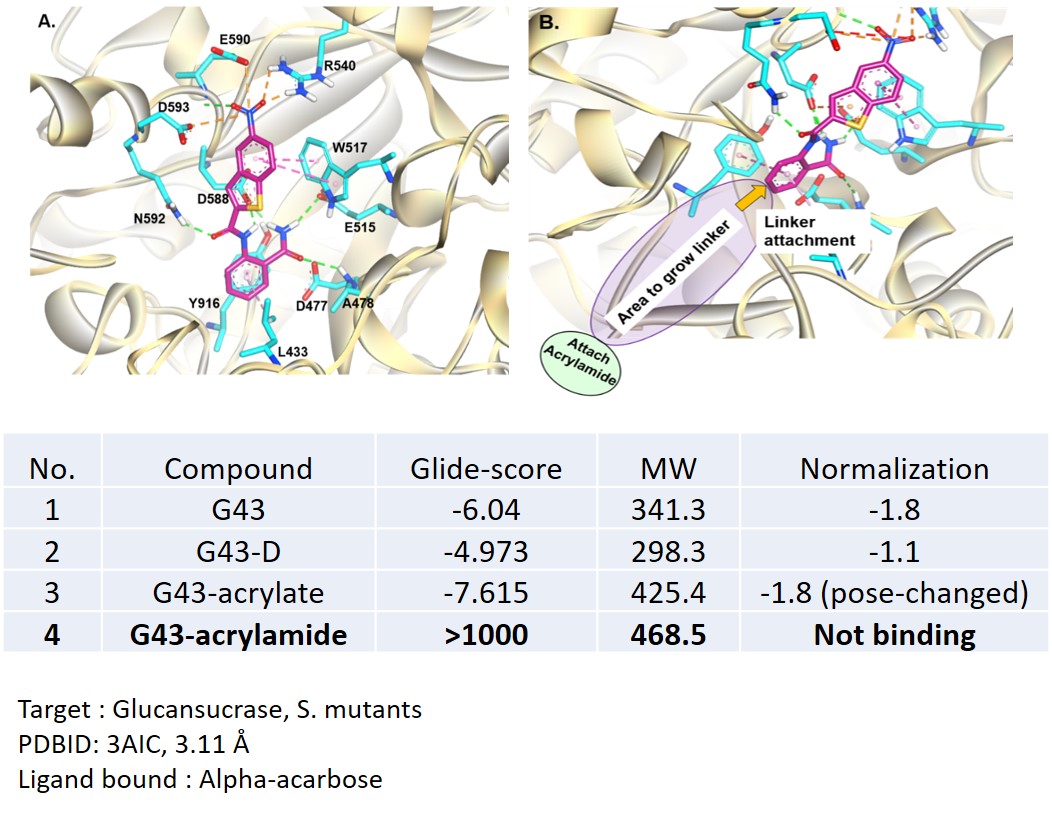

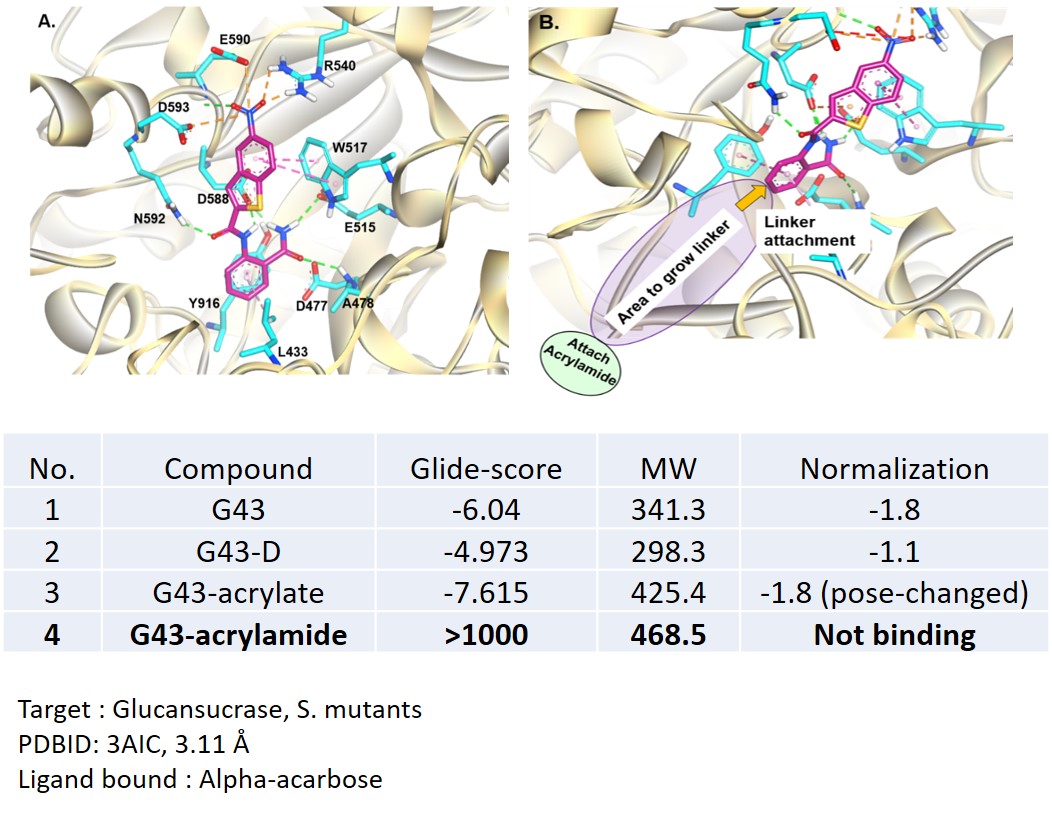


**Figure S9:** *(A)* Induced-Fit docking protocol was to confirm the predicted binding pose for G-43 with GTF-C, which indeed produced strong interaction. *(B)* Preliminary findings with short-chain linkers attached to a polymerizable moiety led to disruption in the binding pose, highlighting the need for longer chain extenders.

Further computational modeling was carried out with PlexView online software (<https://playmolecule.com/PlexView/>) to produce 2D diagrams of protein-ligand interactions. The interactions of the natural form of the enzyme with G43 and the derivatives modified at the C3 position were simulated. In this situation, the ΔG scores in kcal/mol were: G43= -8.59; G43-C3-MeO= -8.95; G43-C3-TEG= -9.66; and G43-C3-TEGMA= -10.10. This is expected, since larger molecules have greater probability of interacting. This modeling confirmed that aspartic acid (ASP 588, Figure S4) belongs to domain A1 of GTF-C, and while it is not the one responsible for the nucleophilic attack, it stabilizes the ligand-enzyme complex, as determined in the models using acarbose.^[6]^ All molecules, except the parent G43, seem to interact with ASP 588. However, these data must be considered carefully, because the real structure of the protein upon interaction with the substrate is not completely elucidated. This will be further investigated in future studies.


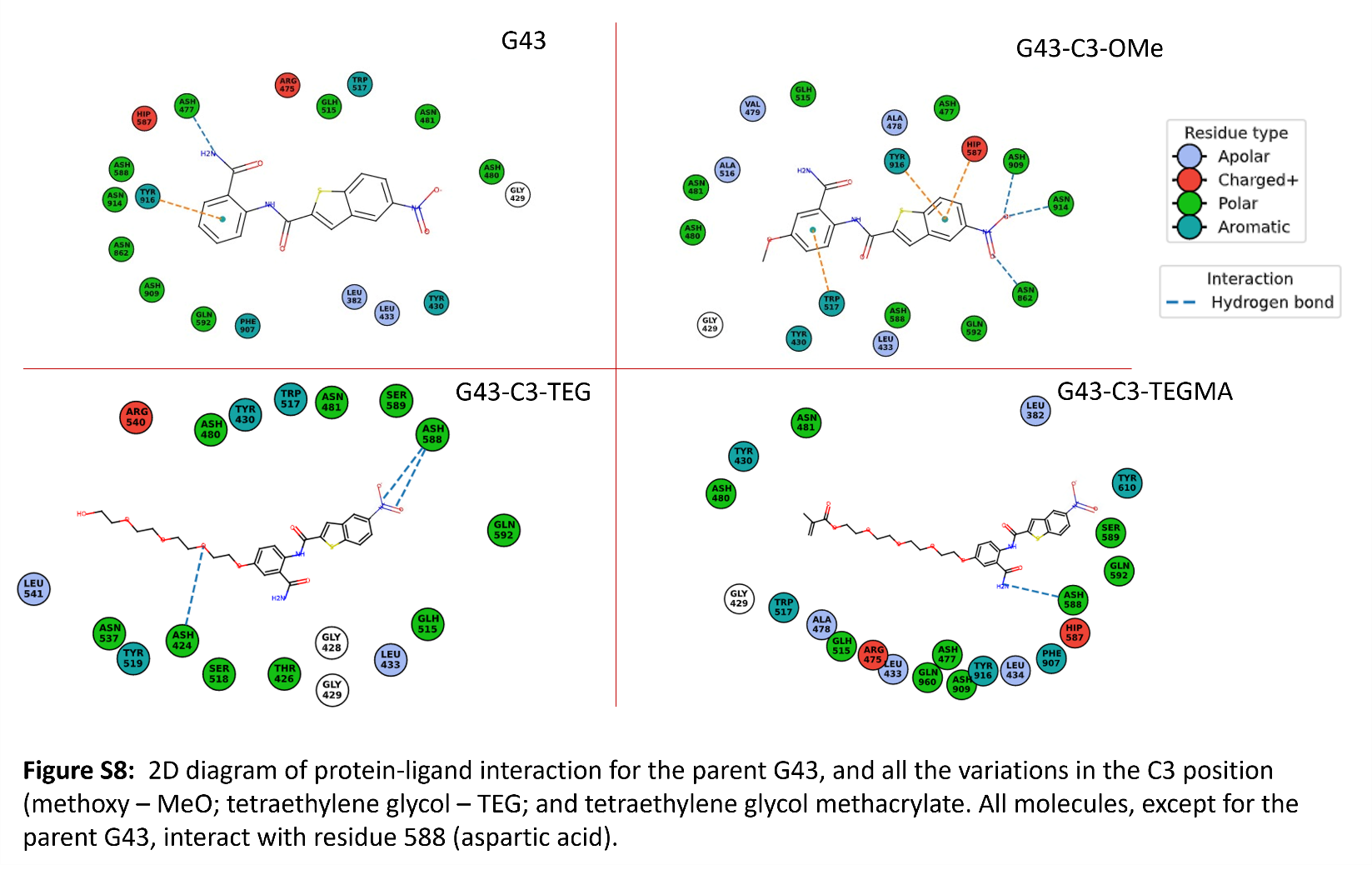


**Figure S10:**  2D diagram of protein-ligand interaction for the parent G43, and all the variations in the C3 position (methoxy – OMe; tetraethylene glycol – TEG; and tetraethylene glycol methacrylate.

# III. Syntheses

## *General Considerations*

### Reagents and Handling

Unless specifically stated otherwise, all reactions and manipulations were carried out with oven*-*dried glassware (180°C) under an inert nitrogen atmosphere using standard Schlenk techniques and glassware. Dry solvents were first purified using fractional distillation followed by storage on 3Å zeolite molecular sieves. All purchased reagents were used “as-is” unless otherwise noted. Acronyms used herein:

Ac = acetyl, THF = Tetrahydrofuran, IR = infrared, NMR = nuclear magnetic resonance, dmso = dimethylsulfoxide, EtOAc = ethyl acetate, brine = saturated NaCl_(aq)_, MP = melting point, DMAP = *N,N-*dimethylaminopyridine, Et = ethyl, Me = methyl, EDAC = 1-ethyl-3-(3ʹ-dimethylaminopropyl)carbodiimide, TLC = thin layer chromatography, DMF = *N,N-*dimethylformamide

### Instrumentation

Nuclear magnetic resonance spectroscopy were collected on either: (a) Bruker 400 MHz Avance II+ spectrometer equipped with a 5 mm BBFO probe at Portland State University (Portland, OR), (b) a Bruker 600 MHz Avance III spectrometer equipped with a 5 mm BBO probe at Portland State University (Portland, OR), or (c) a Bruker (Billerica, MA) Avance NEO 600 MHz spectrometer equipped with a room-temperature TXI probe and with a sample temperature of 25ºC, at Lewis & Clark College (Portland, OR) which was purchased with support from the National Science Foundation (Award 1917696) and the M. J. Murdock Charitable Trust (Grant 201811283).. ^1^H chemical shifts are reported relative to deuterated solvent signals (chloroform-*d*_1_, benzene-*d*_6_, acetone-*d*_6_, or dimethylsulfoxide-*d*_6_). ^1^H NMR data are reported as follows: chemical shift (ppm), multiplicity, coupling constant (Hz), relative integration. ^13^C{^1^H} NMR data are reported as follows: chemical shift (ppm), multiplicity, coupling constant (Hz).

Melting points were measured under air using either a melting point apparatus or a differential scanning calorimeter. High resolution mass spectrometry (HRMS) was performed by the Mass Spectrometry Facility at the University of California Irvine, Irvine CA and validated to within ± 5 ppm. Infrared spectra were taken using a Nicolet 6700 Fourier Transform Infrared Spectrophotometer using KBr discs or a diamond attenuated total reflection (ATR) instrument. Infrared spectroscopy data are reported as follows: peak absorbance (cm^-1^) and intensity (st = strong, m = medium, w = weak, br = broad). Gas chromatography was used to separate mixtures for mass spectrometry and to assess purity: Agilent 6890N gas chromatograph equipped with a 30 meter 0.25 mm diameter column with a (5%-phenyl)-methylpolysiloxane stationary phase (J&W HP-5ms) was used with a split flame ionization detector and Aglient 5973 mass spectrometer equipped with a quadrupole quantitative analysis by electron ionization (EI).

*N-*(2-carbamoylphenyl)-5-nitrobenzo[*b*]thiophene-2-carboxamide (*G43*)

G43 was synthesized following literature procedures with minor modifications.^[2]^ To a dry flask with a magnetic stir-bar were added 2-aminobenzamide (150 mg, 1.1 mmol), 5-nitrobenzo[*b*]thiophene-2-carboxylic acid (268mg, 1.2mmol), 1-Ethyl-3-(3ʹ-dimethylaminopropyl)carbodiimide hydrochloric acid salt (315 mg, 1.6 mmol), and N,*N-*Dimethylpyridi*N-*4-amine (13 mg, 0.1 mmol). The mixture was purged with nitrogen for 15 minutes. The flask was then charged with 7.5 mL of dry dichloromethane and allowed to stir at 23°C for 16 hours resulting in a tan precipitate. The reaction mixture was filtered and the solids washed three times with equivalents of dichloromethane. The solids were collected (crude product) and recrystallized from glacial acetic acid to yield crystalline pure product. The crystals were filtered and vacuumed to dryness, yielding 300mg (80%) of white crystals. ^1^H NMR (400 MHz, dmso-*d*_6_) δ 13.34 (s, 1H), 9.05 (d, *J* = 2.2 Hz, 1H), 8.59 (d, *J* = 8.3 Hz, 1H), 8.50 (s, 1H), 8.44 – 8.33 (m, 1H), 8.33 – 8.23 (m, 2H), 8.02 – 7.85 (m, 2H), 7.61 (t, *J* = 7.9 Hz, 1H), 7.24 (t, *J* = 7.6 Hz, 1H). IR: 3411.8 (m), 3199.0 (m), 3100.0 (w br), 1670.7 (w), 1648.6 (s), 1621.3, 1595.8, 1578.6, 1528.5 (s), 1507.4 (s), 1450.3, 1394.1, 1343.9, 1313.2, 1262.4, 1240.0, 1194.4, 1068.5, 902.8, 824.7, 814.0, 760.0, 740.8, 710.8, 642.4, 583.2. HRMS TOF ES+ [C_16_H_11_N_3_O_4_S + Na]^+^: Calculated: 364.0368 Found: 364.0356.

**Figure S11**. ^1^H NMR spectrum of *N-*(2-carbamoylphenyl)-5-nitrobenzo[*b*]thiophene-2-carboxamide (*G43*).


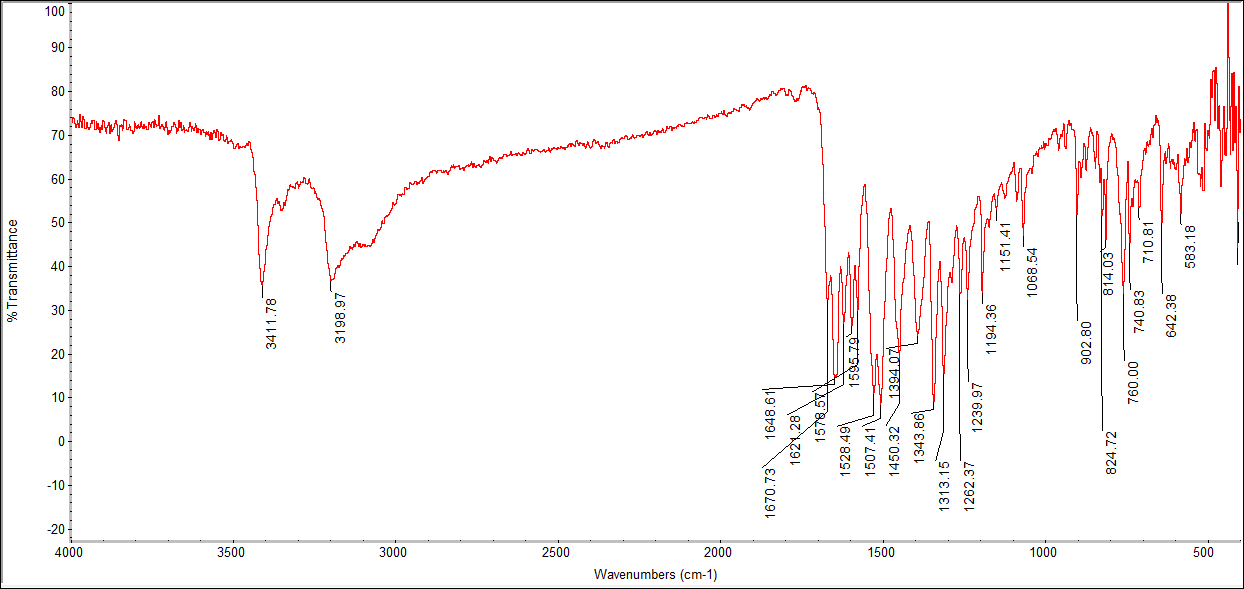


**Figure S12**. Mid-infrared absorbance spectrum of *N-*(2-carbamoylphenyl)-5-nitrobenzo[*b*]thiophene-2-carboxamide (*G43*).

G43-C3-TEG-MA Synthetic Overview

5-fluoro-2-nitrobenzamide

5-fluoro-2-nitrobenzamide was synthesized following literature procedures with minor modifications.[3] To a dry flask equipped with a reflux condenser and a magnetic stir-bar was added 5-fluoro-2-nitrobenzoic acid (100 mg, 0.54 mmol). The reaction vessel was purged with nitrogen for 15 minutes. The vessel was then charged with 5 mL of dry tetrahydrofuran and a solution of thionyl chloride (120 µL, 1.62 mmol) in 5mL of tetrahydrofuran. The reaction was heated to reflux for 16 hours. The reaction was then cooled to 0°C and quenched with 1.0 mL of 30% NH_4_OH(aq). The resulting mixture was diluted with 20 mL of saturated NaHCO_3_(aq) and 20 mL ethyl acetate and the organic layer isolated in a sepeartory funnel. The organic layer was washed once with equivalent NaHCO_3_(aq) followed by an equivalent of brine. The organic layer was isolated and dried on Na_2_SO_4_ for one hour, filtered, and solvent removed under reduced pressure to yield a waxy yellow solid (crude product). The crude product was purified by crystallization from toluene followed by recrystallization from chloroform, yielding off-white needles (96mg, 96% yield). MP: 130-132°C. ^1^H NMR (400 MHz, Acetone-*d*_6_) δ 8.14 (dd, *J* = 9.9, 4.7 Hz, 1H), 7.57 (s, 1H), 7.52 – 7.35 (m, 2H), 7.11 (s, 1H). IR: 3444.5, 3280.3, 1656.5, 1583.7, 1527.4, 1421.3, 1341.5, 1221.6, 873.7, 843.8, 831.0, 616.9 cm^-1^.

**Figure S13**. ^1^H NMR spectrum of 5-fluoro-2-nitrobenzamide.


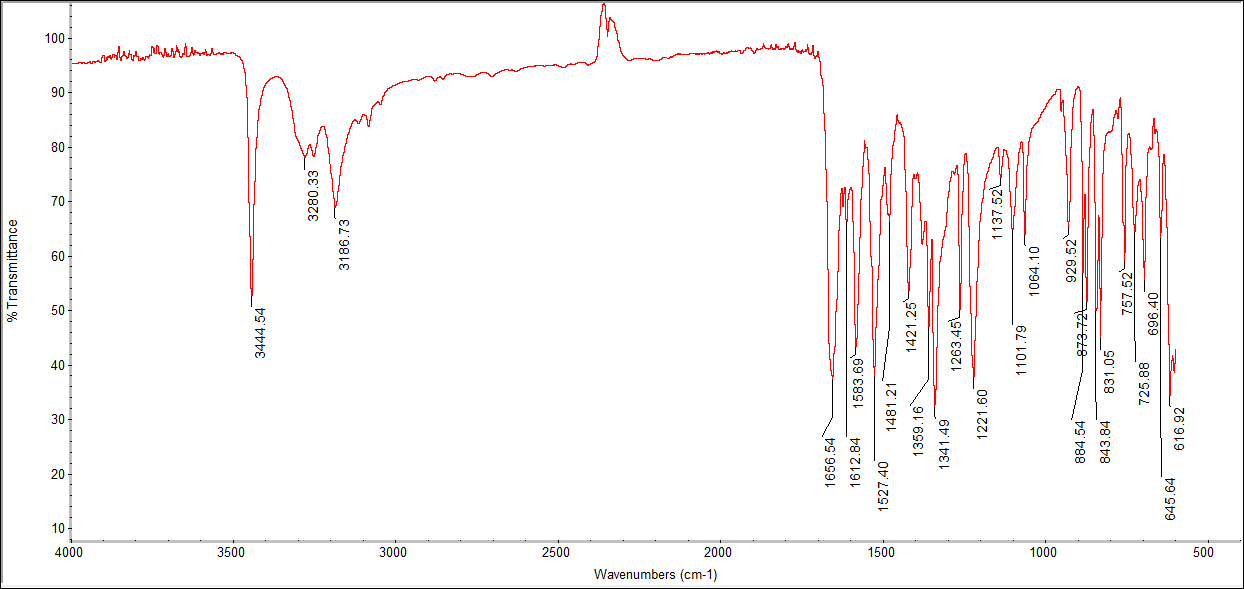


**Figure S14**. Mid-infrared absorbance spectrum of 5-fluoro-2-nitrobenzamide.

5-(2-(2-(2-(2-hydroxyethoxy)ethoxy)ethoxy)ethoxy)-2-nitrobenzamide.

A dry flask equipped with a pressure-equalizing addition funnel and magnetic stir bar was charged with dry bis[2-(2-hydroxyethoxy)ethyl] ether (2.22g, 11.4mmol) and 100mL of dry THF. The flask was purged with N_2_ for 15 minutes. Under a back-flow of N_2_, solid NaH (60 wt% dispersion in mineral oil, 440mg, 10.9mmol) was added slowly to the solution with vigorous stirring. Caution! *Reaction evolves a stoichiometric quantity of H_2_*. The mixture was allowed to stir for 15 minutes at 23°C, whereupon it developed a slightly yellow color. 5-fluoro-2-nitrobenzamide (1.91g, 10.4 mmol) was dissolved in 100mL of dry THF, purged with N_2_ for 15 minutes, and transferred to the pressure equalizing-addition funnel. The reaction mixture was cooled with an ice bath, and the 5-fluoro-2-nitrobenzamide solution was added dropwise to the reaction over 25 minutes. The reaction was allowed to warm to room temperature and stir overnight under N_2_. The reaction was quenched with 1 gram of NH_4_Cl_(s)_ followed by 2mL of H_2_O – immediately bleaching the reaction color. The quench was allowed to stir for 15 minutes before workup. The reaction mixture was filtered through diatomaceous earth, and rinsed twice with aliquots of THF. The filtrate was reduced to a thick oil by vacuum removal of volatiles. Extended exposure to high-vacuum yielded an orange-tinted wax (crude reaction product). The crude reaction product was triturated against boiling toluene three times, yielding a colorless organic layer and an immiscible red oil (crude product). The crude product was then crystalized from CHCl_3_ to yield off-white crystals (2.01g, 54% yield). (Note that the mother liquor contained more product that could be recovered.) MP: 63-65°C. ^1^H NMR (400 MHz, Chloroform-*d*) δ 8.01 (d, *J* = 9.0 Hz, 1H), 6.91 (dd, *J* = 9.1, 2.8 Hz, 1H), 6.88 (s, 1H), 6.86 (d, *J* = 2.7 Hz, 1H), 6.71 (s, 1H), 4.11 – 4.04 (m, 2H), 3.81 – 3.76 (m, 2H), 3.73 – 3.62 (m, 12H), 3.58 (ddd, *J* = 8.4, 5.0, 3.3 Hz, 3H). IR: 3600.5, 3383.6, 3203.1, 2901.5, 1666.1, 1584.7, 1505.3, 1429.9, 1335.6, 1252.9, 1055.6, 963.6, 936.3, 823.9, 621.7 cm^-1^.

**Figure S15**. ^1^H NMR spectrum of 5-(2-(2-(2-(2-hydroxyethoxy)ethoxy)ethoxy)ethoxy)-2-nitrobenzamide.


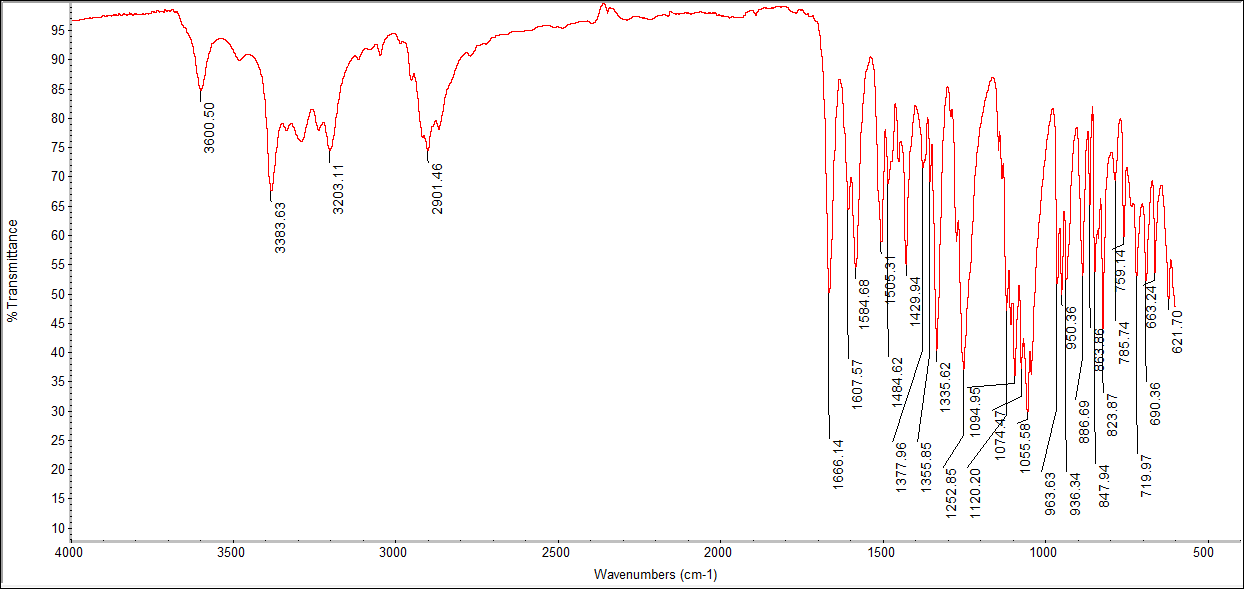


**Figure S16**. Mid-infrared absorbance spectrum of 5-(2-(2-(2-(2-hydroxyethoxy)ethoxy)ethoxy)ethoxy) -2-nitrobenzamide.

2-(2-(2-(2-(3-carbamoyl-4-nitrophenoxy)ethoxy)ethoxy)ethoxy)ethyl acetate.

A dry flask equipped with a pressure-equalizing addition funnel and magnetic stir bar was charged with dry 5-(2-(2-(2-(2-hydroxyethoxy)ethoxy)ethoxy)ethoxy)-2-nitrobenzamide (1.09g, 2.8mmol), dry trimethylamine (770µL, 5.6mmol) and 25mL of dry THF at 23°C. The flask was purged with N_2_ for 15 minutes. Acetic anhydride (400 µL, 4.2mmol) was dissolved in 25mL of dry THF, purged with N_2_ for 15 minutes, and transferred to the pressure equalizing-addition funnel. The acetic anhydride solution was added dropwise to the reaction over 15 minutes. The reaction was stirred overnight under N_2_. The reaction mixture was poured directly into a separatory funnel and washed twice against equal volumes of saturated bicarbonate solution. Caution! *Saturated bicarbonate will release CO_2_, pressurizing a closed separatory funnel.* If emulsions occurred, Et_2_O was added to help break the phases up. The organic layer was isolated, dried on Na_2_SO_4_, filtered, and volatiles were removed via vacuum, yielding a yellow oil (crude product). The crude product was purified by flash column chromatography using 1 hexanes: 1 acetone eluent. A faintly yellow oil was isolated that crystalized over time (650mg, 58% yield). MP: 82-83°C. ^1^H NMR (400 MHz, Chloroform-*d*) δ 8.07 (d, *J* = 9.0 Hz, 1H), 7.12 – 6.90 (m, 2H), 6.23 (d, *J* = 55.1 Hz, 2H), 4.24 (dd, *J* = 5.6, 3.6 Hz, 2H), 4.20 – 4.11 (m, 2H), 3.87 (dd, *J* = 5.6, 3.5 Hz, 2H), 3.73 – 3.57 (m, 11H), 2.06 (s, 3H). IR: 3370.7, 3184.4, 2905.8, 1726.1, 1659.7, 1586.0, 1511.3, 1352.4, 1243.3, 1126.0, 1102.0, 1037.6, 860.1, 662.3 cm^-1^.

**Figure S17**. ^1^H NMR spectrum of 2-(2-(2-(2-(3-carbamoyl-4-nitrophenoxy)ethoxy)ethoxy)ethoxy)ethyl acetate.


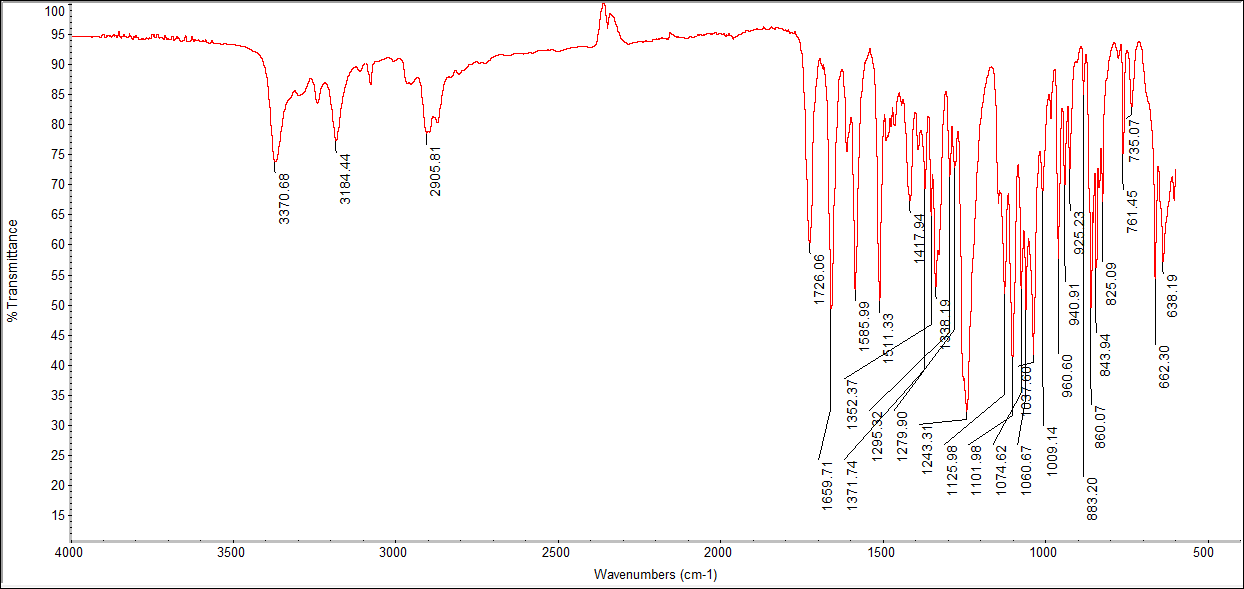


**Figure S18**. Mid-infrared absorbance spectrum of 2-(2-(2-(2-(3-carbamoyl-4-nitrophenoxy)ethoxy)ethoxy)ethoxy)ethyl acetate.

2-(2-(2-(2-(4-amino-3-carbamoylphenoxy)ethoxy)ethoxy)ethoxy)ethyl acetate.

To a dry flask were added 2-(2-(2-(2-(3-carbamoyl-4-nitrophenoxy)ethoxy)ethoxy)ethoxy)ethyl acetate (600mg, 1.5mmol), 25mg Palladium on carbon (Pd/C, 10 wt. % Pd loading on a matrix activated carbon support), and 50mL of 190 proof EtOH. The reaction mixture was purged with H2 three times using a balloon fitted to the flask. The reaction vessel with a balloon was charged a final time (H_2_, ~800mL, 1atm) and allowed to stir overnight at 23°C. The reaction was judged complete by TLC (1 hexanes: 2 acetone, R_f_^product^ = 0.45, fluorescent blue under long-waved UV (λ=365nm)). The reaction mixture was filtered through diatomaceous earth, and rinsed twice with aliquots of THF. The resulting solution was concentrated via vacuum to a slightly yellow oil (crude, 550mg, 1.5mmol). *Note that this compound is not light/air-stable and decomposes completely over several days with the development of a red color.* This compound was telescoped to the next reaction without further purification.

2-(2-(2-(2-(3-carbamoyl-4-(5-nitrobenzo[*b*]thiophene-2-carboxamido)phenoxy)ethoxy)ethoxy)ethoxy)ethyl acetate.

To a dry flask containing crude 2-(2-(2-(2-(4-amino-3-carbamoylphenoxy)ethoxy)ethoxy)ethoxy)ethyl acetate (550mg, 1.5mmol) was added to 5-nitrobenzo[*b*]thiophene-2-carboxylic acid (333mg, 1.43mmol, purchased from AmBeed), 1-Ethyl-3-(3ʹ-dimethylaminopropyl)carbodiimide hydrochloric acid salt (431.3mg, 2.3 mmol), and DMAP (20mg, 0.15mmol). The flask was purged for 15 minutes with N_2_. The reaction flask was then charged with 50mL dry CH_2_Cl_2_ and allowed to stir at 23°C overnight under N_2_. The solution was initially soluble and forms precipitate over time. The reaction was filtered, and washed twice with CH_2_Cl_2_. The solids were taken up into acetone, combined with a small amount of flash grade silica, and the volatiles were removed via vacuum. The dry-loaded silica was used to purify the crude product by flash column chromatography using EtOAc with 1% trimethylamine as eluent (R_f_ = 0.45). A tan solid was isolated (510mg, 62%). MP: 170-175°C. IR: 3364.8, 3183.7, 2871.4, 1736.2, 1660.3, 1532.7, 1509.9, 1341.7, 1247.6, 1229.6, 1057.9, 819.4, 640.3 cm^-1^. ^1^H NMR (400 MHz, Chloroform-*d*) δ 12.56 (s, 1H), 8.78 (d, *J* = 2.1 Hz, 1H), 8.69 (d, *J* = 9.1 Hz, 1H), 8.26 (dd, *J* = 8.9, 2.2 Hz, 1H), 8.06 (s, 1H), 7.99 (d, *J* = 8.9 Hz, 1H), 7.39 (d, *J* = 2.8 Hz, 1H), 7.13 (dd, *J* = 9.2, 2.9 Hz, 1H), 6.97 (s, 1H), 5.93 (s, 1H), 4.28 – 4.23 (m, 2H), 4.21 (dd, *J* = 5.6, 3.6 Hz, 2H), 3.84 (dd, *J* = 5.6, 3.5 Hz, 2H), 3.76 – 3.63 (m, 10H), 2.10 (s, 3H).

**Figure S19**. ^1^H NMR spectrum of 2-(2-(2-(2-(3-carbamoyl-4-(5-nitrobenzo[*b*]thiophene-2-carboxamido)phenoxy)ethoxy)ethoxy)ethoxy)ethyl acetate.


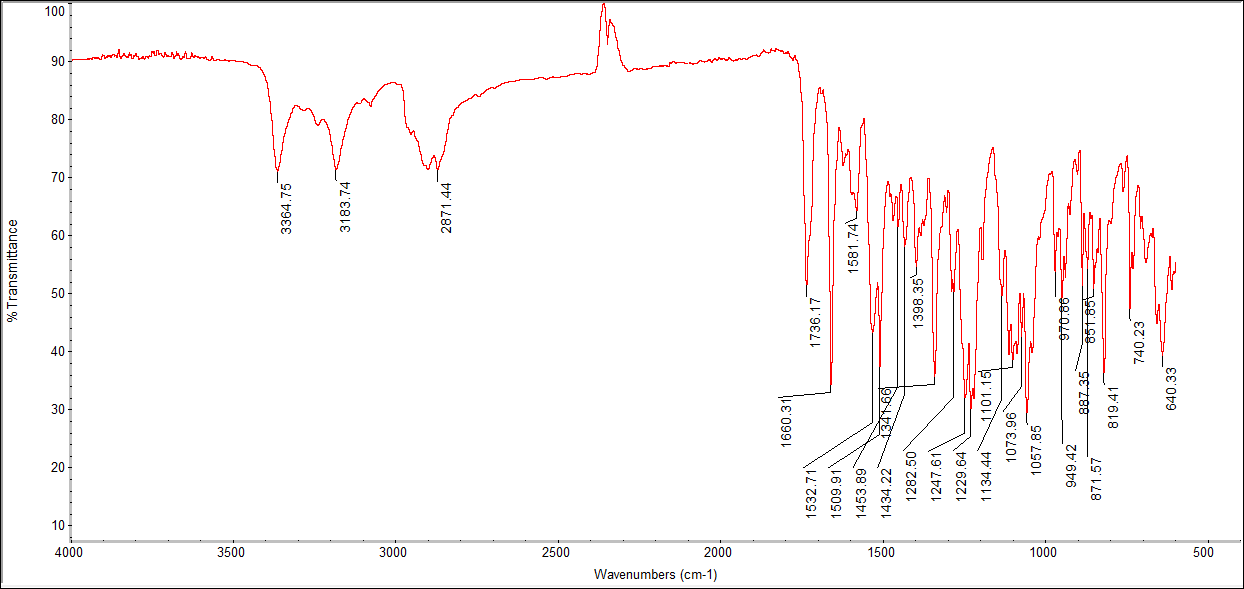


**Figure S20**. Mid-infrared absorbance spectrum of 2-(2-(2-(2-(3-carbamoyl-4-(5-nitrobenzo[*b*]thiophene-2-carboxamido)phenoxy)ethoxy)ethoxy)ethoxy)ethyl acetate.

*N-*(2-carbamoyl-4-(2-(2-(2-(2-hydroxyethoxy)ethoxy)ethoxy)ethoxy)phenyl)-5-nitrobenzo[*b*]thiophene-2-carboxamide (G43-C3-TEG).

To a flask equipped with a magnetic stir bar was added 2-(2-(2-(2-(3-carbamoyl-4-(5-nitrobenzo[*b*]thiophene-2-carboxamido)phenoxy)ethoxy)ethoxy)ethoxy)ethyl acetate (300mg, 0.5mmol) followed with 3mL of THF and 1mL of MeOH. The mixture was allowed to stir at 23°C. In a second flask, LiOH (50mg, 2.1mmol, anhydrous) was dissolved into 1.0mL of H_2_O. The LiOH solution was added dropwise to the stirring reaction solution, developing a yellow-orange color. The reaction was deemed to be complete within 5 minutes by TLC (Acetone, R_f_^product^ = 0.75). The reaction was quenched with 10mL 1M NaHCO_3(aq)_, leading to a faintly yellow precipitate. The reaction mixture was filtered, and the solids were washed three times with H_2_O, after which the solids were allowed to dry on the filter paper. The crude product solids were collected and triturated against toluene (to drive out extra water) and volatiles were removed via vacuum. The solids were then taken up into acetone, filtered through celite, and vacuum dried to a slightly yellow powdery solid (255mg, 95%). ^1^H NMR (400 MHz, dmso-*d*_6_) δ 13.00 (s, 1H), 9.03 (d, *J* = 2.2 Hz, 1H), 8.48 (d, *J* = 9.0 Hz, 2H), 8.35 (d, *J* = 8.9 Hz, 1H), 8.31 – 8.20 (m, 2H), 7.90 (s, 1H), 7.52 (d, *J* = 2.9 Hz, 1H), 7.21 (dd, *J* = 9.2, 2.8 Hz, 1H), 4.58 (t, *J* = 5.4 Hz, 1H), 4.17 (t, *J* = 4.5 Hz, 2H), 3.78 (dd, *J* = 5.6, 3.6 Hz, 2H), 3.64 – 3.55 (m, 4H), 3.53 (s, 4H), 3.49 (t, *J* = 5.3 Hz, 2H), 3.42 (t, *J* = 5.2 Hz, 2H). ^13^C{^1^H} NMR (151 MHz, dmso-*d*_6_) δ 170.61, 158.49, 153.96, 146.06, 145.43, 143.73, 138.95, 132.65, 125.51, 124.28, 121.82, 121.28, 120.57, 120.23, 118.56, 114.35, 72.34, 69.94, 69.85, 69.83, 69.77, 68.90, 67.52, 60.21. IR: 3542.2 (m), 3426.4 (m), 3199.3 (br), 2903.0 (m, br), 1659.4, 1508.7, 1342.1, 1246.5, 1131.9, 1091.5, 959.5, 841.0, 825.44, 740.0. HRMS TOF ES+ [C_24_H_27_N_3_O_9_S + Na]^+^ : Calculated: 556.1366 Found: 556.1379.

**Figure S21**. ^1^H NMR spectrum of *N-*(2-carbamoyl-4-(2-(2-(2-(2-hydroxyethoxy)ethoxy)ethoxy)ethoxy)phenyl)-5-nitrobenzo[*b*]thiophene-2-carboxamide (G43-C3-TEG).

**Figure S22**. ^13^C{^1^H} NMR spectrum of *N-*(2-carbamoyl-4-(2-(2-(2-(2-hydroxyethoxy)ethoxy)ethoxy)ethoxy)phenyl)-5-nitrobenzo[*b*]thiophene-2-carboxamide (G43-C3-TEG).


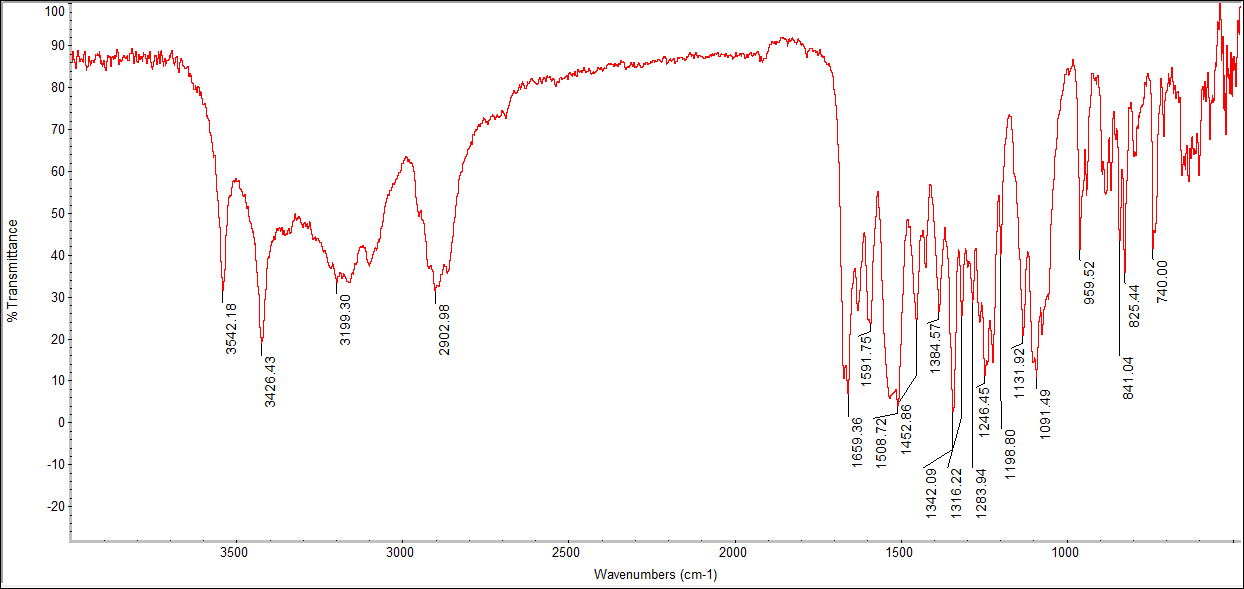


**Figure S23**. Mid-infrared absorbance spectrum of *N-*(2-carbamoyl-4-(2-(2-(2-(2-hydroxyethoxy)ethoxy)ethoxy)ethoxy)phenyl)-5-nitrobenzo[*b*]thiophene-2-carboxamide (G43-C3-TEG).

2-(2-(2-(2-(3-carbamoyl-4-(5-nitrobenzo[*b*]thiophene-2-carboxamido)phenoxy)ethoxy)ethoxy)ethoxy)ethyl methacrylate (G43-C3-TEG-MA).

To a dry flask was added *N-*(2-carbamoyl-4-(2-(2-(2-(2-hydroxyethoxy)ethoxy)ethoxy)ethoxy)phenyl)-5-nitrobenzo[*b*]thiophene-2-carboxamide (200mg, 0.37mmol) and then purged with N_2_ for 15 min. The flask was then charged with dry trimethylamine (200µL, 1.5mmol) and 10mL of dry THF. While stirring at 23°C, methacrylic anhydride (100µL, 0.68mmol) was added to the reaction slowly over 5 minutes. The reaction was allowed to stir under N_2_ at 23°C overnight. The reaction was quenched with 10mL of saturated NaHCO_3(aq)_ and the organic layer was collected. The organic layer was reduced to solids via vacuum. The solids were taken up into acetone, filtered through diatomaceous earth, combined with a small amount of flash grade silica, and the volatiles were removed via vacuum. The dry-loaded silica was used to purify the crude product by flash column chromatography using EtOAc with 1% trimethylamine as eluent (R_f_ = 0.46). A faintly yellow solid was isolated (98.6mg, 46%). ^1^H NMR (400 MHz, dmso-*d*_6_) δ 13.00 (s, 1H), 9.05 (d, *J* = 2.3 Hz, 1H), 8.49 (d, *J* = 9.1 Hz, 2H), 8.37 (d, *J* = 8.9 Hz, 1H), 8.32 – 8.22 (m, 2H), 7.90 (s, 1H), 7.52 (d, *J* = 2.9 Hz, 1H), 7.22 (dd, *J* = 9.2, 2.9 Hz, 1H), 6.03 (dd, *J* = 1.8, 1.0 Hz, 1H), 5.68 (q, *J* = 1.6 Hz, 1H), 4.29 – 4.08 (m, 4H), 3.83 – 3.73 (m, 2H), 3.71 – 3.63 (m, 2H), 3.63 – 3.48 (m, 9H), 3.31 (s, 1H), 1.88 (t, *J* = 1.3 Hz, 3H). ^13^C{^1^H} NMR (151 MHz, dmso-*d*_6_) δ 170.59, 158.56, 154.00, 146.07, 145.49, 143.72, 138.99, 135.78, 132.61, 125.80, 125.59, 124.34, 121.88, 121.34, 120.69, 120.28, 118.60, 114.38, 69.92, 69.84, 69.76, 68.89, 68.24, 67.53, 64.44, 63.71, 60.20, 17.97. IR: 3372.5 (m, br), 3198.3 (w), 2875.3 (w), 1716.60 (m), 1661.09, 1537.7, 1398.0, 1342.5, 1094.5 (br), 818.0, 740.0 (w). HRMS TOF ES+ [C_28_H_31_N_3_O_10_S + Na]^+^ : Calculated: 624.1628 Found: 624.1637.

**Figure S24**. ^1^H NMR spectrum of 2-(2-(2-(2-(3-carbamoyl-4-(5-nitrobenzo[*b*]thiophene-2-carboxamido)phenoxy)ethoxy)ethoxy)ethoxy)ethyl methacrylate (G43-C3-TEG-MA).

**Figure S25**. ^13^C{^1^H} NMR spectrum of 2-(2-(2-(2-(3-carbamoyl-4-(5-nitrobenzo[*b*]thiophene-2-carboxamido)phenoxy)ethoxy)ethoxy)ethoxy)ethyl methacrylate (G43-C3-TEG-MA).


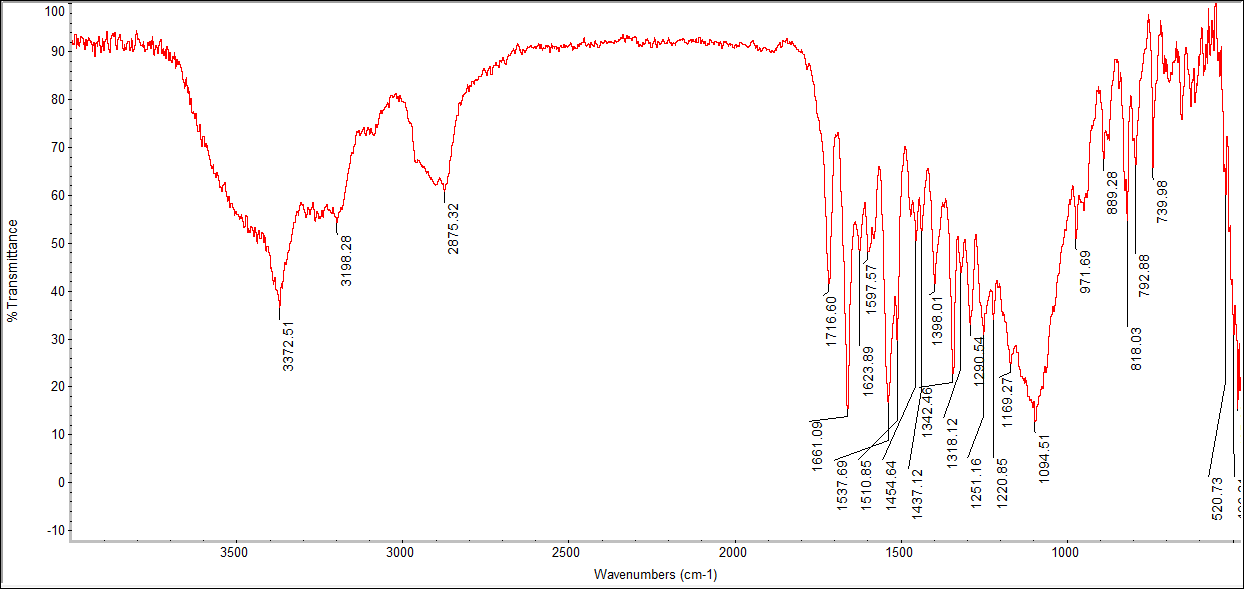


**Figure S26**. Mid-infrared absorbance spectrum of 2-(2-(2-(2-(3-carbamoyl-4-(5-nitrobenzo[*b*]thiophene-2-carboxamido)phenoxy)ethoxy)ethoxy)ethoxy)ethyl methacrylate (G43-C3-TEG-MA).

**G16 Synthetic Overview**

This molecule was initially included in the experimental design because it showed promise in a previous publication. The initial screening failed to reproduce those previous results, so this molecule was not tested further. The synthesis procedure is included here for completeness’ sake. G16 was synthesized following an analogous compound in the literature^[4]^ using the general scheme:

1-(4-(4-fluorobenzoyl)phenyl)-2-phenylethane-1,2-dione.

To a flask fitted with a reflux condenser was added (4-fluorophenyl)(4-(phenylethynyl)phenyl)methanone (500mg, 1.7mmol, Sigma-Aldrich), 0.8mL H_2_O, 0.3mL AcOH, and 20mL acetone. While at 23°C, KMnO_4_ (580mg, 3.7mmol) was added slowly as a solid. The reaction was brought to reflux for 2.5 hours. The reaction was deemed to be complete by TLC (20% EtOAc in Hex, R_f_^product^ = 0.4, yellow under UV light). The crude reaction mixture was filtered hot through diatomaceous earth and rinsed three times with boiling acetone. The filtrate was reduced in volume (via vacuum) until a cloudiness appeared. This mixture was added to 100mL of boiling water, which was allowed to cool slowly, yielding fine crystalline precipitate over time. After cooling the mixture overnight, the mixture was filtered and dried via vacuum which yielded a faintly yellow crystalline solid (490mg, 87%). The spectra collected matched literature values.^[4]^ MP: 112-115°C. ^1^H NMR (400 MHz, Chloroform-*d*) δ 8.10 (d, *J* = 8.1 Hz, 2H), 8.22 – 7.95 (m, 2H), 7.85 (dd, *J* = 8.4, 5.7 Hz, 4H), 7.73 – 7.65 (m, 1H), 7.54 (t, *J* = 7.7 Hz, 2H), 7.18 (t, *J* = 8.6 Hz, 2H). IR: 3064.4, 2922.5, 1659.2, 1594.0, 1496.9, 1400.8, 1269.8, 1209.1, 929.2, 884.3, 717.2, 663.8, cm^-1^.

**Figure S27**. ^1^H NMR spectrum of 1-(4-(4-fluorobenzoyl)phenyl)-2-phenylethane-1,2-dione.


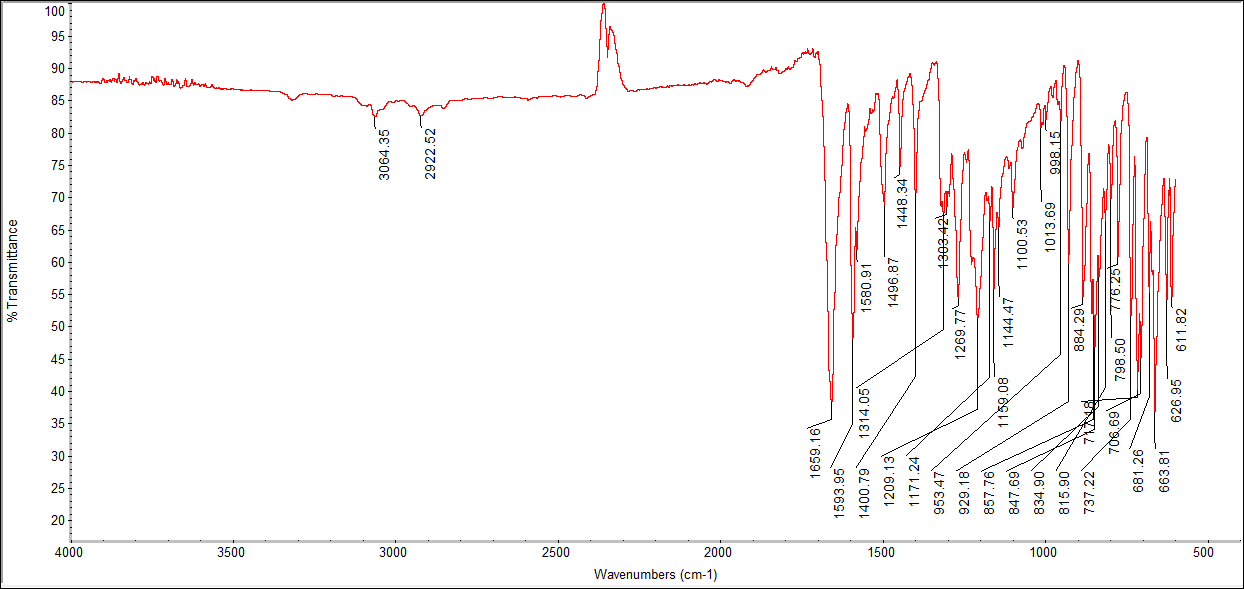


**Figure S28**. Mid-infrared absorbance spectrum of 1-(4-(4-fluorobenzoyl)phenyl)-2-phenylethane-1,2-dione.

(4-fluorophenyl)(4-(2-(2-hydroxy-4-nitrophenyl)-4-phenyl-1*H*-imidazol-5-yl)phenyl)methanone (G16).

To a dry flask equipped with a reflux condenser was added 1-(4-(4-fluorobenzoyl)phenyl)-2-phenylethane-1,2-dione (150mg, 0.45mmol), 2-hydroxy-4-nitrobenzaldehyde (77mg, 0.46mmol, AmBeed), and NH_4_OAc (243mg, 3.2mmol). The reaction vessel was then charged with 2mL of glacial AcOH and brought to a steady reflux for 10 hours. The reaction formed a yellow precipitate over time. The reaction was allowed to cool, then poured slowly into 50mL of saturated NaHCO_3(aq)_. This mixure was filtered and the solids washed three times with H_2_O. The crude yellow precipitate was triturated with boiling EtOH, boiling toluene, and re-filtered and dried. The resulting solid yellow powder was pure product (205mg, 95%). ^1^H NMR (400 MHz, dmso-*d*_6_) δ 8.30 (d, *J* = 8.7 Hz, 1H), 7.86 (td, *J* = 8.4, 7.1, 3.8 Hz, 3H), 7.80 – 7.74 (m, 3H), 7.74 – 7.67 (m, 2H), 7.63 – 7.56 (m, 2H), 7.56 – 7.44 (m, 2H), 7.40 (t, *J* = 8.7 Hz, 2H). ^13^C{^1^H} NMR (151 MHz, dmso-*d_6_*) δ 193.71, 147.70, 143.73, 136.06, 132.53, 132.46, 130.13, 129.16, 129.01, 128.88, 126.53, 126.00, 118.69, 118.13, 115.84, 115.75, 115.59, 115.43, 114.12, 111.56, 111.12, 30.68. IR: 3055.1, 1654.6, 1597.7, 1502.7, 1336.7, 1300.9, 1224.3, 1021.8, 812.1, 768.5, 736.2, 678.7 cm^-1^. HRMS TOF ES+ [C_28_H_18_FN_3_O_4_ + Na]^+^ : Calculated: 480.1360 Found: 480.1349.

**Figure S29**. ^1^H NMR spectrum of (4-fluorophenyl)(4-(2-(2-hydroxy-4-nitrophenyl)-4-phenyl-1H-imidazol-5-yl)phenyl)methanone (G16).

**Figure S30**. ^13^C{^1^H} NMR spectrum of (4-fluorophenyl)(4-(2-(2-hydroxy-4-nitrophenyl)-4-phenyl-1*H*-imidazol-5-yl)phenyl)methanone (G16).


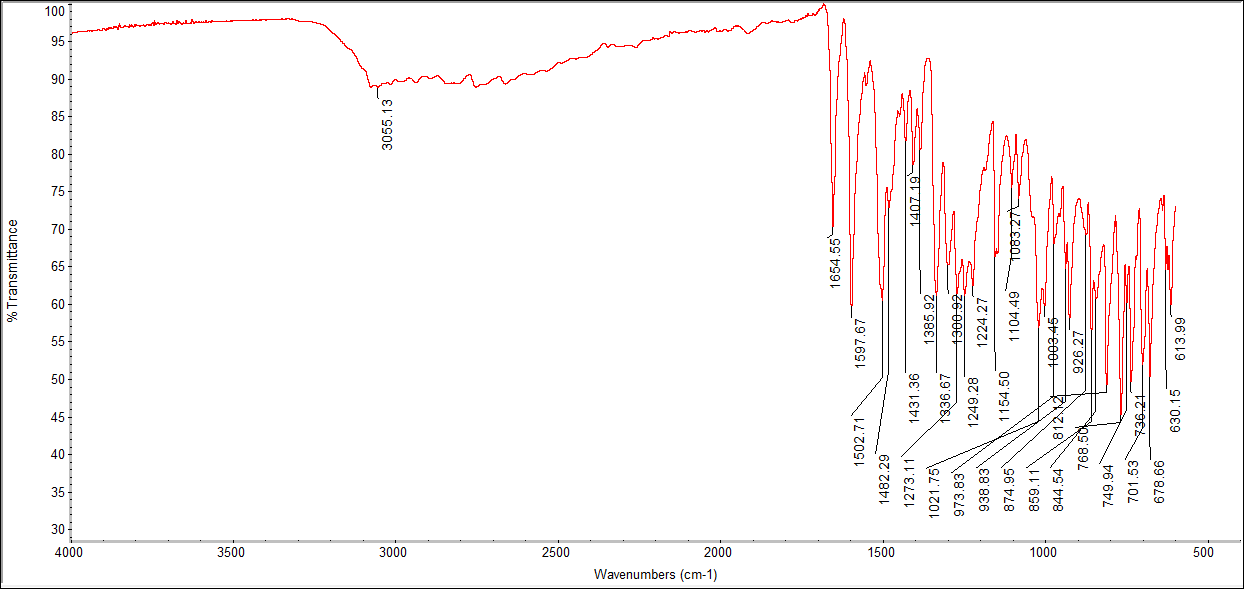


**Figure S31**. Mid-infrared absorbance spectrum of

G43-A2-OMe Synthetic Overview

2-fluoro-4-methoxy-5-nitrobenzaldehyde.

The following reaction is a modification of a literature preparation.^[5]^ To a flask containing 2.0g 2-fluoro-4-methoxybenzaldehyde wad added H_2_SO_4_ (25mL, 98% grade) and the reaction was cooled using an ice bath. HNO_3(aq)_ (862µL, 68% grade) was added slowly over 10 minutes. After the addition, the reaction was allowed to warm to room temperature and stir for 2 hours. The reaction was deemed complete by quenching small aliquots and performing TLC to observe complete conversion (20% EtOAc in hexanes, R_f_^product^ = 0.1, R_f_^sm^ = 0.5). The reaction was quenched by slowly pouring the reaction mixture into 300mL of chilled H_2_O in a separatory funnel and washed against 100mL Et_2_O. The organic layer was separated and the aqueous layer was re-washed with another 50mL of Et_2_O. The organic layers were combined, washed against 100mL of brine, separated, dried on Na_2_SO_4_, filtered, and volatiles were removed via vacuum yielding crude product. The product was purified by two recrystalizations out of EtOH, yielding pure colorless crystals (1.41g, 54%). MP: 100-101°C. ^1^H NMR (400 MHz, dmso-*d*_6_) δ 10.06 (s, 1H), 8.41 (d, *J* = 7.3 Hz, 1H), 7.51 (d, *J* = 12.6 Hz, 1H), 4.05 (s, 3H). IR: 3080.5, 2879.4, 1686.5, 1618.8, 1529.2, 1437.9, 1307.7, 1289.6, 1148.3, 1061.1, 989.2, 856.2, 759.4 cm^-1^.

**Figure S32**. ^1^H NMR spectrum of 2-fluoro-4-methoxy-5-nitrobenzaldehyde.


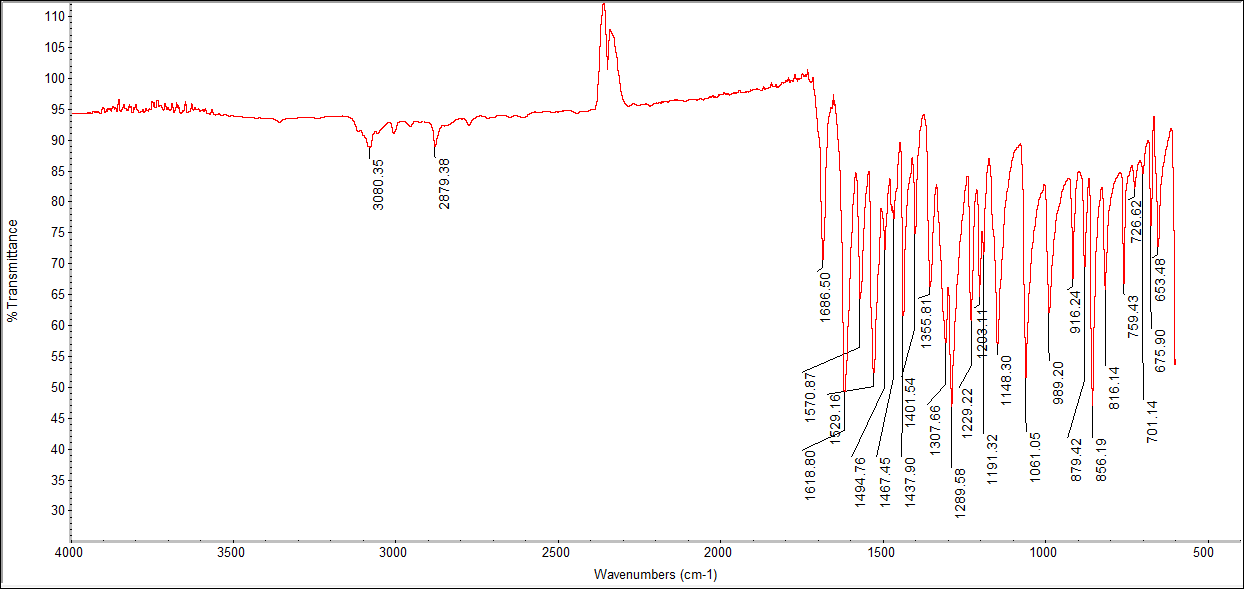


**Figure S33**. Mid-infrared absorbance spectrum of 2-fluoro-4-methoxy-5-nitrobenzaldehyde.

Methyl 6-methoxy-5-nitrobenzo[*b*]thiophene-2-carboxylate.

To a dry flask was added dry DMF (10mL), K_2_CO_3_ (624mg, 4.5mmol, anhydrous), and methyl 2-mercaptoacetate (480mg, 4.5mmol). Caution! *methyl 2-mercaptoacetate is extremely malodorous – handle only in the fume hood and clean surfaces afterwards with bleach*. A solution of 2-fluoro-4-methoxy-5-nitrobenzaldehyde (300mg, 1.5mmol) in DMF (10mL) was added dropwise to the reaction mixture over 5 minutes. The reaction was heated to 80°C for 2 hours. The reaction was deemed complete by TLC (50% Et_2_O in hexanes, R_f_^product^ = 0.57). Upon completion, the reaction mixture (a dark red thick gel when cooled), was poured into 100mL of boiling H_2_O and allowed to cool overnight. A tan precipitate was collected by filtration (note that the filtrate will be used to isolate more product), yielding product (162mg, 43%). MP: 190-192°C. IR: 3094.1, 2965.6, 2921.3, 2850.7, 1701.3, 1607.1, 1524.6, 1292.8, 1231.8, 1168.0, 1064.7, 1029.2, 981.5, 751.2, 723.6 cm^-1^. ^1^H NMR (400 MHz, Chloroform-*d*) δ 8.33 (s, 1H), 8.02 (s, 1H), 7.46 (s, 1H), 4.04 (s, 3H), 3.96 (s, 3H).

The aqueous filtrate was then acidified and extracted with EtOAc to yield 6-methoxy-5-nitrobenzo[*b*]thiophene-2-carboxylic acid (140mg, 37%). *See next entry below for spectra.*

**Figure S34**. ^1^H NMR spectrum of Methyl 6-methoxy-5-nitrobenzo[*b*]thiophene-2-carboxylate.


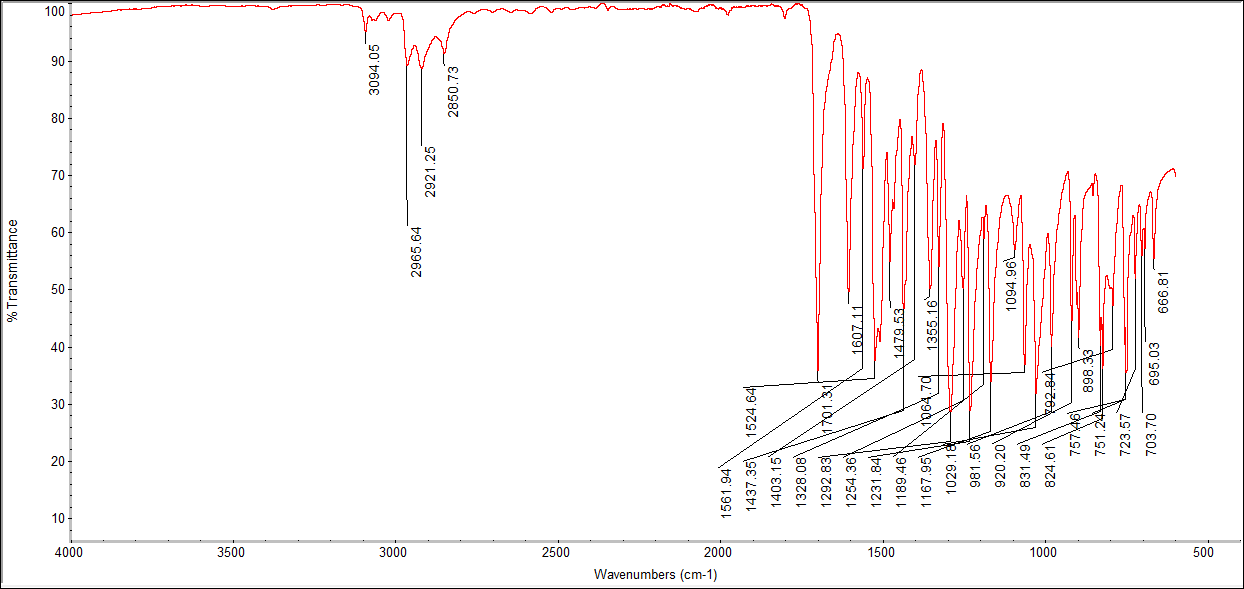


**Figure S35**. Mid-infrared absorbance spectrum of Methyl 6-methoxy-5-nitrobenzo[*b*]thiophene-2-carboxylate.

6-methoxy-5-nitrobenzo[*b*]thiophene-2-carboxylic acid.

To a flask equipped with a magnetic stir bar was added methyl 6-methoxy-5-nitrobenzo[*b*]thiophene-2-carboxylate (100mg, 0.37mmol) followed with 3mL of THF and 1mL of MeOH. The mixture was allowed to stir at 23°C. In a second flask, LiOH (50mg, 2.1mmol, anhydrous) was dissolved into 1.0mL of H_2_O. The LiOH solution was added dropwise to the stirring reaction solution, developing a deep red color. The reaction was deemed to be complete within 4 hours by TLC (50% Et_2_O in hexanes, R_f_^product^ = 0.15). The reaction was quenched with HCl, leading to a yellow precipitate. The reaction mixture was filtered, and the solids were washed three times with H_2_O, after which the solids were allowed to dry on the filter paper. The crude product solids were collected and triturated against toluene (to drive out extra water) and volatiles were removed via vacuum. The solids were then taken up into acetone, filtered through celite, and vacuum dried to a slightly yellow powdery solid (39.2mg, 42%). MP: 260°C (Decomp.). ^1^H NMR (400 MHz, dmso-*d*_6_) δ 13.62 (s, 1H), 8.58 (s, 1H), 8.13 (s, 1H), 8.05 (s, 1H), 3.98 (s, 3H). IR: 2842.8, 2581.8, 1670.2, 1608.7, 1521.6, 1429.6, 1352.6, 1295.9, 1235.5, 1175.2, 1033.7, 983.1, 915.7, 822.0, 760.1, 695.5, 662.8.

**Figure S36**. ^1^H NMR spectrum of 6-methoxy-5-nitrobenzo[*b*]thiophene-2-carboxylic acid.


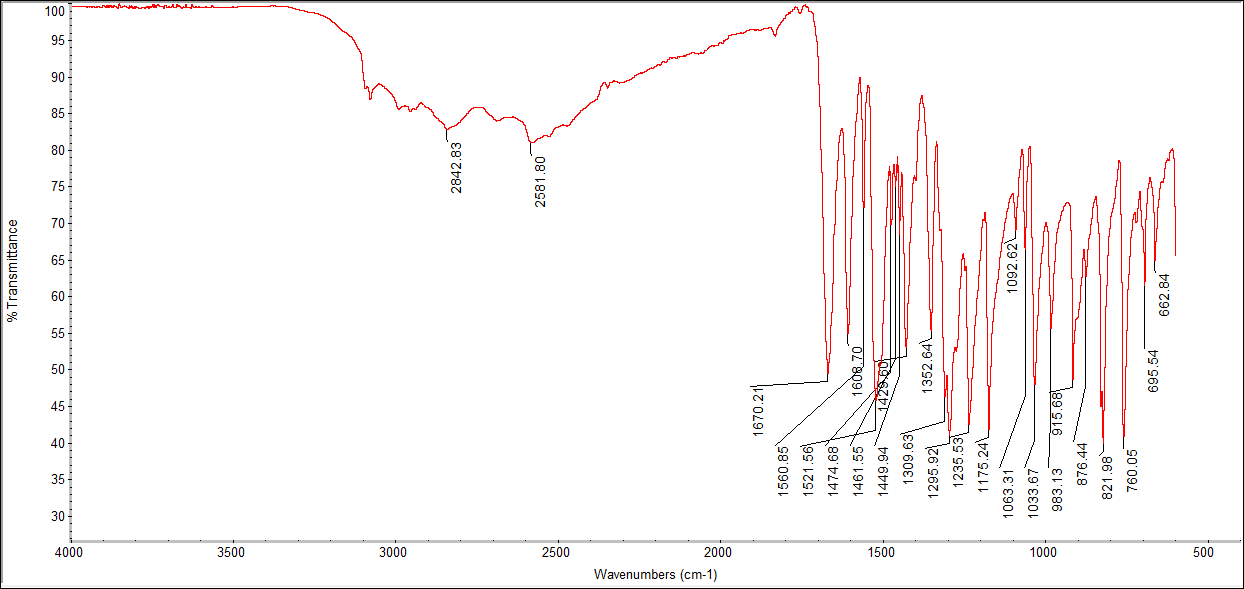


**Figure S37**. Mid-infrared absorbance spectrum of 6-methoxy-5-nitrobenzo[*b*]thiophene-2-carboxylic acid.

*N-*(2-carbamoylphenyl)-6-methoxy-5-nitrobenzo[*b*]thiophene-2-carboxamide (G43-A2-OMe).

To a dry flask was added 6-methoxy-5-nitrobenzo[*b*]thiophene-2-carboxylic acid (79mg, 0.31mmol), 2-aminobenzamide (46.7 mg, 0.34 mmol), 1-Ethyl-3-(3ʹ-dimethylaminopropyl)carbodiimide hydrochloric acid salt (89 mg, 0.47 mmol), and DMAP (4 mg, 0.03 mmol). The mixture was purged with nitrogen for 15 minutes. The flask was then charged with 7.5 mL of dry dichloromethane and allowed to stir at 23°C for 16 hours resulting in a tan precipitate. The reaction mixture was filtered and the solids washed three times with equivalents of dichloromethane. The solids were collected and vacuumed to dryness, yielding (55.3mg, 48%). IR: 3402.5 (m, br), 3183.9 (br), 1681.7, 1637.5, 1581.7, 1504.7, 1461.6, 1444.8, 1380.0, 1340.8, 1325.7, 1291.4, 1234.1, 1059.9, 878.6, 767.2, 740.3, 670.8, 631.3. ^1^H NMR (600 MHz, dmso-*d*_6_) δ 8.65 (s, 1H), 8.57 (d, *J* = 8.7 Hz, 2H), 8.48 (s, 2H), 8.07 (d, *J* = 14.5 Hz, 3H), 7.98 – 7.88 (m, 3H), 7.59 (t, *J* = 7.8 Hz, 2H), 7.21 (t, *J* = 7.7 Hz, 2H), 3.99 (s, 5H). ^13^C{^1^H} NMR (151 MHz, dmso-*d_6_*) δ 171.04, 159.17, 150.26, 145.76, 140.49, 139.54, 139.26, 132.70, 131.37, 128.80, 125.13, 123.06, 122.01, 120.11, 119.05, 107.13, 57.07.. HRMS TOF ES+ [C_17_H_13_N_3_O_5_S + Na]^+^ : Calculated: 394.0474 Found: 394.0471.

**Figure S38**. ^1^H NMR spectrum of *N-*(2-carbamoylphenyl)-6-methoxy-5-nitrobenzo[*b*]thiophene-2-carboxamide (G43-A2-OMe).

**Figure S39**. ^13^C{^1^H} NMR spectrum of *N-*(2-carbamoylphenyl)-6-methoxy-5-nitrobenzo[*b*]thiophene-2-carboxamide (G43-A2-OMe).


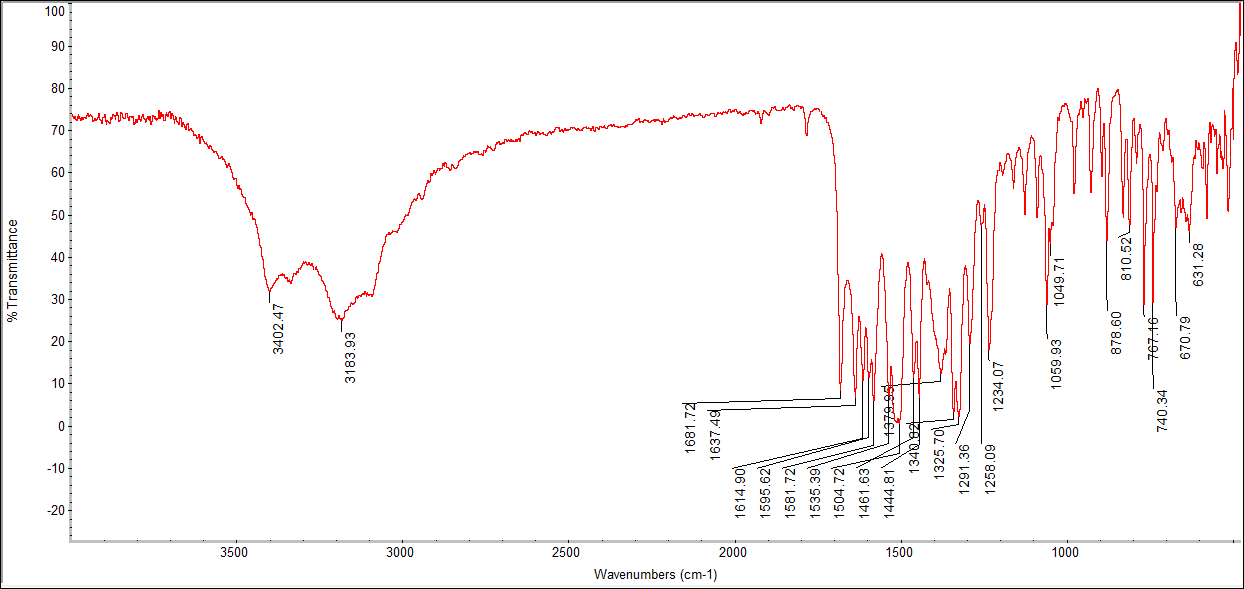


**Figure S40**. Mid-infrared absorbance spectrum of *N-*(2-carbamoylphenyl)-6-methoxy-5-nitrobenzo[*b*]thiophene-2-carboxamide (G43-A2-OMe).

G43-B1-OMe Synthetic Overview

G43-B1-OMe was synthesized following an analogous compound in the literature^[4]^ using the general scheme:

Methyl 3-hydroxy-5-nitrobenzo[*b*]thiophene-2-carboxylate.

To a dry flask was added dry DMF (10mL), K_2_CO_3_ (624mg, 4.5mmol, anhydrous), and methyl 2-mercaptoacetate (480mg, 4.5mmol). Caution! *methyl 2-mercaptoacetate is extremely malodorous – handle only in the fume hood and clean surfaces afterwards with bleach.* A solution of methyl 2-fluoro-5-nitrobenzoate (300mg, 1.5mmol) in DMF (10mL) was added dropwise to the reaction mixture over 5 minutes. The reaction was heated to 80°C for 2 hours. The reaction was deemed complete by TLC (50% Et_2_O in hexanes, R_f_^product^ = 0.6). Upon completion, the reaction mixture (a dark red thick gel when cooled), was poured into 100mL of boiling H_2_O and allowed to cool overnight. A tan precipitate was collected by filtration and recrystallized from 2-butanone, yielding a yellow cotton candy-like crystalline product (219mg, 61%). MP: 360°C (decomp.). ^1^H NMR (400 MHz, dmso-*d*_6_) δ 8.36 (d, *J* = 2.4 Hz, 1H), 8.07 (dd, *J* = 8.7, 2.4 Hz, 1H), 7.75 (d, *J* = 8.7 Hz, 1H), 3.59 (s, 3H). IR: 3270.5 (br), 3066.1, 1677.2, 1586.2, 1502.6, 1340.7, 1314.1, 1217.3, 1185.6, 1132.8, 1050.5, 984.7, 912.7, 818.9, 767.7, 735.8, 638.1 cm^-1^.

**Figure S41**. ^1^H NMR spectrum of Methyl 3-hydroxy-5-nitrobenzo[*b*]thiophene-2-carboxylate.


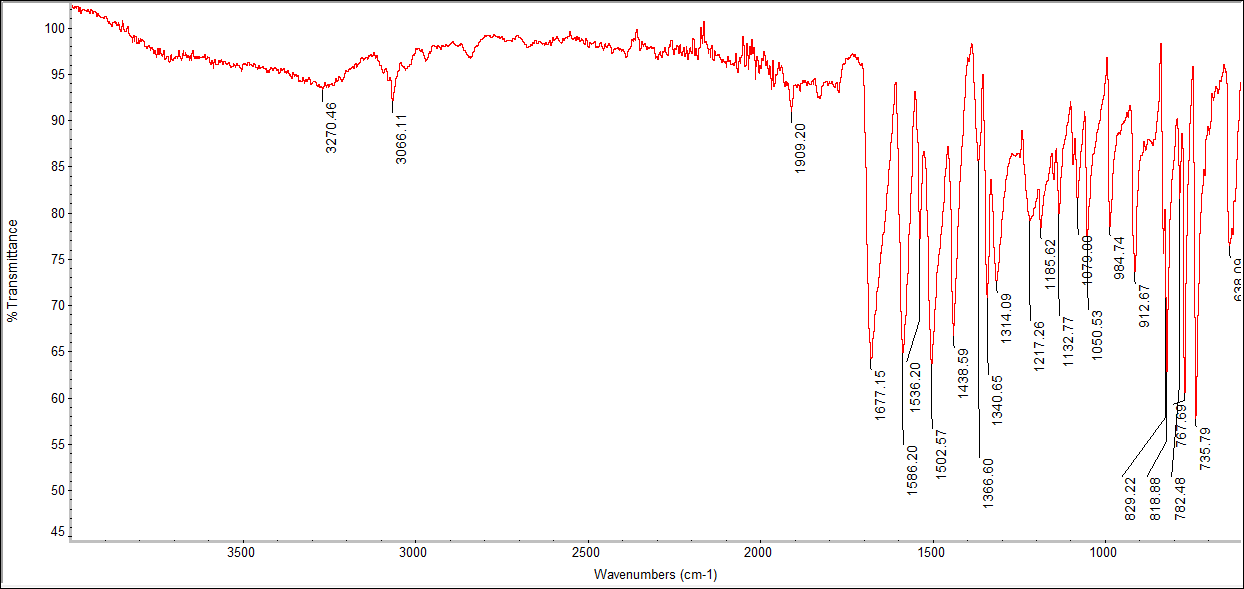


**Figure S42**. Mid-infrared absorbance spectrum of Methyl 3-hydroxy-5-nitrobenzo[*b*]thiophene-2-carboxylate.

Methyl 3-methoxy-5-nitrobenzo[*b*]thiophene-2-carboxylate.

To a dry flask was added K_2_CO_3_ (553mg, 6.0mmol, anhydrous), DMF (50mL), and 3-hydroxy-5-nitrobenzo[*b*]thiophene-2-carboxylic acid (359mg, 1.5mmol). The reaction was allowed to react at room temperature overnight. The reaction mixture was filtered through diatomaceous earth to remove trace solids. The resulting solution was slowly added to 400mL H_2_O forming a flocculent precipitate. The precipitates were isolated by filtration and recrystallized using EtOH/H_2_O as a faintly green solid (254mg, 63%). MP: 153-155°C. ^1^H NMR (400 MHz, Chloroform-*d*) δ 8.76 (d, *J* = 2.2 Hz, 1H), 8.30 (dd, *J* = 9.0, 2.2 Hz, 1H), 7.86 (d, *J* = 8.9 Hz, 1H), 4.24 (s, 3H), 3.95 (s, 3H). IR: 3084.0, 2923.4, 2853.9, 1719.5, 1595.3, 1577.2, 1538.9, 1508.1, 1452.4, 1316.6, 1262.6, 1231.9, 1192.7, 1138.1, 1047.5, 947.9, 834.1, 737.7.

**Figure S43**. ^1^H NMR spectrum of Methyl 3-methoxy-5-nitrobenzo[*b*]thiophene-2-carboxylate.


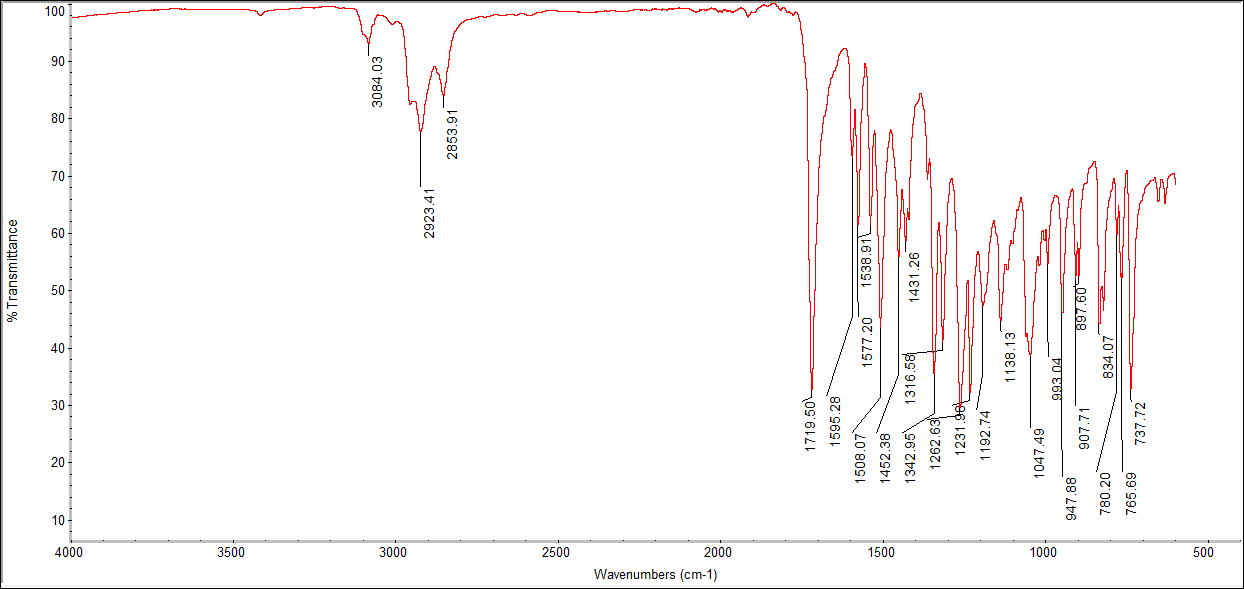


**Figure S44**. Mid-infrared absorbance spectrum of Methyl 3-methoxy-5-nitrobenzo[*b*]thiophene-2-carboxylate.

3-methoxy-5-nitrobenzo[*b*]thiophene-2-carboxylic acid.

To a flask equipped with a magnetic stir bar was added methyl 3-methoxy-5-nitrobenzo[*b*]thiophene-2-carboxylate (54mg, 0.20mmol) followed with 3mL of THF and 1mL of MeOH. The mixture was allowed to stir at 23°C. In a second flask, LiOH (18mg, 0.75 mmol, anhydrous) was dissolved into 1.0mL of H_2_O. The LiOH solution was added dropwise to the stirring reaction solution. The reaction was deemed to be complete within 4 hours by TLC. The reaction was quenched with HCl, leading to a yellow precipitate. The reaction mixture was filtered, and the solids were washed three times with H_2_O, after which the solids were allowed to dry on the filter paper. The crude product solids were collected and triturated against toluene (to drive out extra water) and volatiles were removed via vacuum. The solids were then taken up into hot acetone, filtered through diatomaceous earth, and vacuum dried to a slightly crystalline white solid (45.6mg, 90%). MP: 260°C (Decomp). ^1^H NMR (400 MHz, dmso-*d*_6_) δ 13.72 (s, 1H), 8.58 (d, *J* = 2.1 Hz, 1H), 8.32 (dd, *J* = 8.9, 2.2 Hz, 1H), 8.25 (d, *J* = 8.9 Hz, 1H), 4.15 (s, 3H). IR: 2843.4 (br), 2624.7 (w), 1687.0, 1654.7, 1601.7, 1573.7, 1535.3, 1517.6, 1467.9, 1342.0, 1321.4, 1283.8, 1240.2, 1064.5, 966.3.

**Figure S45**. ^1^H NMR spectrum of 3-methoxy-5-nitrobenzo[*b*]thiophene-2-carboxylic acid**.**


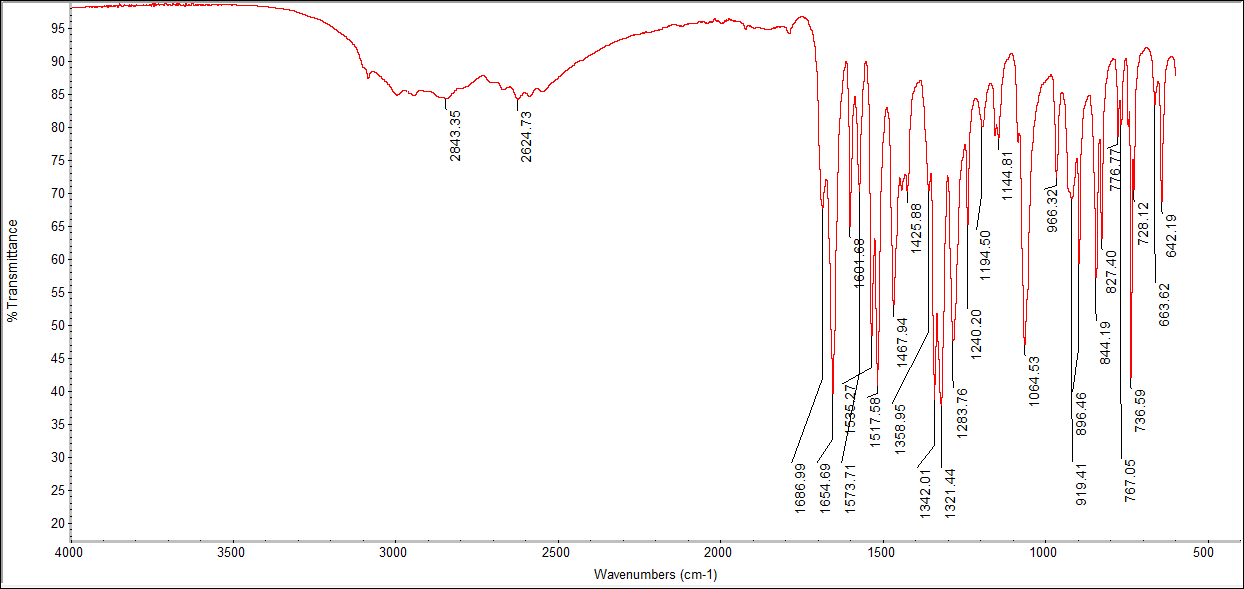


**Figure S46**. Mid-infrared absorbance spectrum of 3-methoxy-5-nitrobenzo[*b*]thiophene-2-carboxylic acid**.**

*N-*(2-carbamoylphenyl)-3-methoxy-5-nitrobenzo[*b*]thiophene-2-carboxamide (G43-B1-OMe).

To a dry flask was added 3-methoxy-5-nitrobenzo[*b*]thiophene-2-carboxylic acid (153mg, 0.6mmol), 2-aminobenzamide (90.5 mg, 0.66 mmol), 1-Ethyl-3-(3ʹ-dimethylaminopropyl)carbodiimide hydrochloric acid salt (184 mg, 0.96 mmol), and DMAP (8.1 mg, 0.06 mmol). The mixture was purged with nitrogen for 15 minutes. The flask was then charged with 7.5 mL of dry dichloromethane and allowed to stir at 23°C for 16 hours resulting in a faintly yellow precipitate. The reaction mixture was filtered and the solids washed three times with equivalents of dichloromethane followed by three washes of THF. The solids were collected and vacuumed to dryness, yielding (172mg, 77%). ^1^H NMR (400 MHz, dmso-*d*_6_) δ 12.35 (s, 1H), 8.81 (d, *J* = 1.3 Hz, 1H), 8.59 (d, *J* = 8.4 Hz, 1H), 8.31 (d, *J* = 1.3 Hz, 2H), 7.80 (d, *J* = 7.0 Hz, 2H), 7.59 – 7.49 (m, 1H), 7.30 – 7.17 (m, 3H). ^13^C{^1^H} NMR 151 MHz, dmso-*d_6_*) δ 170.29, 158.89, 151.28, 145.16, 142.93, 138.04, 132.25, 131.75, 128.54, 125.87, 125.33, 123.30, 122.05, 121.40, 121.00, 118.71, 62.82. IR: 3429.8, 3331.4, 3081.4, 1674.3, 1654.1, 1620.6, 1584.2, 1519.3, 1449.9, 1344.7, 1317.3, 1287.1, 1263.6, 1174.8, 1073.5, 876.6, 761.5, 684.5, 630.7. HRMS TOF ES+ [C_17_H_13_N_3_O_5_S + Na]^+^ : Calculated: 394.9474 Found: 394.0471.

**Figure S47**. ^1^H NMR spectrum of *N-*(2-carbamoylphenyl)-3-methoxy-5-nitrobenzo[*b*]thiophene-2-carboxamide (G43-B1-OMe)**.**

**Figure S48**. ^13^C{^1^H} NMR spectrum of *N-*(2-carbamoylphenyl)-3-methoxy-5-nitrobenzo[*b*]thiophene-2-carboxamide (G43-B1-OMe)**.**


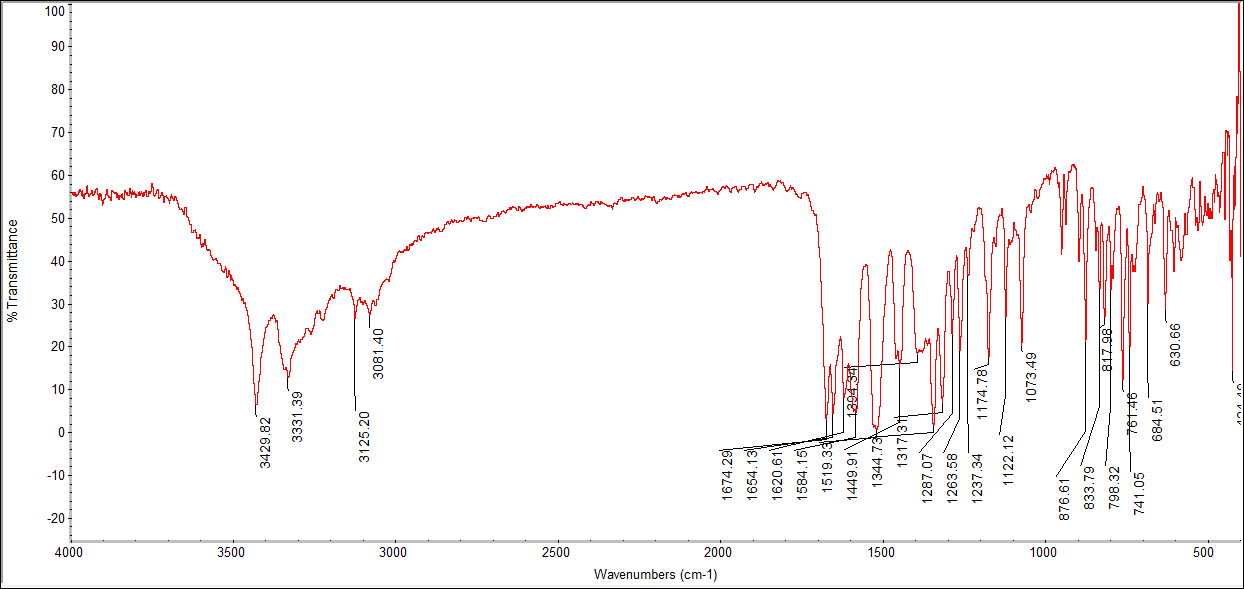


**Figure S49**. Mid-infrared absorbance spectrum of *N-*(2-carbamoylphenyl)-3-methoxy-5-nitrobenzo[*b*]thiophene-2-carboxamide (G43-B1-OMe)**.**

*N-*(2-carbamoyl-4-methoxyphenyl)-5-nitrobenzo[*b*]thiophene-2-carboxamide (G43-C3-OMe).

To a dry flask was added 5-nitrobenzo[*b*]thiophene-2-carboxylic acid (74mg, 0.33mmol), 2-amino-5-methoxybenzamide (50 mg, 0.3 mmol), 1-Ethyl-3-(3ʹ-dimethylaminopropyl)carbodiimide hydrochloric acid salt (86.5 mg, 0.45 mmol), and DMAP (3.7 mg, 0.03 mmol). The mixture was purged with nitrogen for 15 minutes. The flask was then charged with 3 mL of dry dichloromethane and allowed to stir at 23°C for 16 hours resulting in a faintly yellow precipitate. The reaction mixture was filtered and the solids washed three times with equivalents of dichloromethane followed by three washes of THF. The solids were collected and vacuumed to dryness, yielding (110mg, 98%). ^1^H NMR (600 MHz, DMSO-*d*_6_) δ 8.99 (s, 1H), 8.57 – 8.40 (m, 2H), 8.32 (d, *J* = 9.5, 3.5 Hz, 1H), 8.25 (d, *J* = 8.5, 2.6 Hz, 1H), 8.21 (s, 1H), 7.90 (s, 1H), 7.52 – 7.42 (m, 1H), 7.17 (d, *J* = 8.6, 2.7 Hz, 1H), 3.95 – 3.73 (m, 3H). ^13^C{^1^H} NMR (151 MHz, DMSO) δ 170.60, 158.49, 154.77, 146.05, 145.41, 143.72, 138.94, 132.50, 125.49, 124.26, 121.89, 121.25, 120.75, 120.20, 118.13, 113.65, 55.56. IR: 3428.3, 3355.7, 3236.7, 3074.1, 2942.6, 2842.8, 1657.4, 1630.4, 1592.6, 1532.2, 1445.3, 1425.9, 1393.4, 1342.8, 1316.6, 1295.5, 1251.0, 1233.3, 1186.3, 1087.1,1066.9, 1047.1, 898.3, 826.7, 736.9, 551.6. HRMS TOF ESI+ [C_17_H_13_N_3_O_5_S+Na]^+^: Calculated: 394.0474 Found: 394.0468.

**Figure S50**. ^1^H NMR spectrum of *N-*(2-carbamoyl-4-methoxyphenyl)-5-nitrobenzo[*b*]thiophene-2-carboxamide (G43-C3-OMe)**.**

**Figure S51**. ^13^C{^1^H} NMR spectrum of *N-*(2-carbamoyl-4-methoxyphenyl)-5-nitrobenzo[*b*]thiophene-2-carboxamide (G43-C3-OMe)**.**


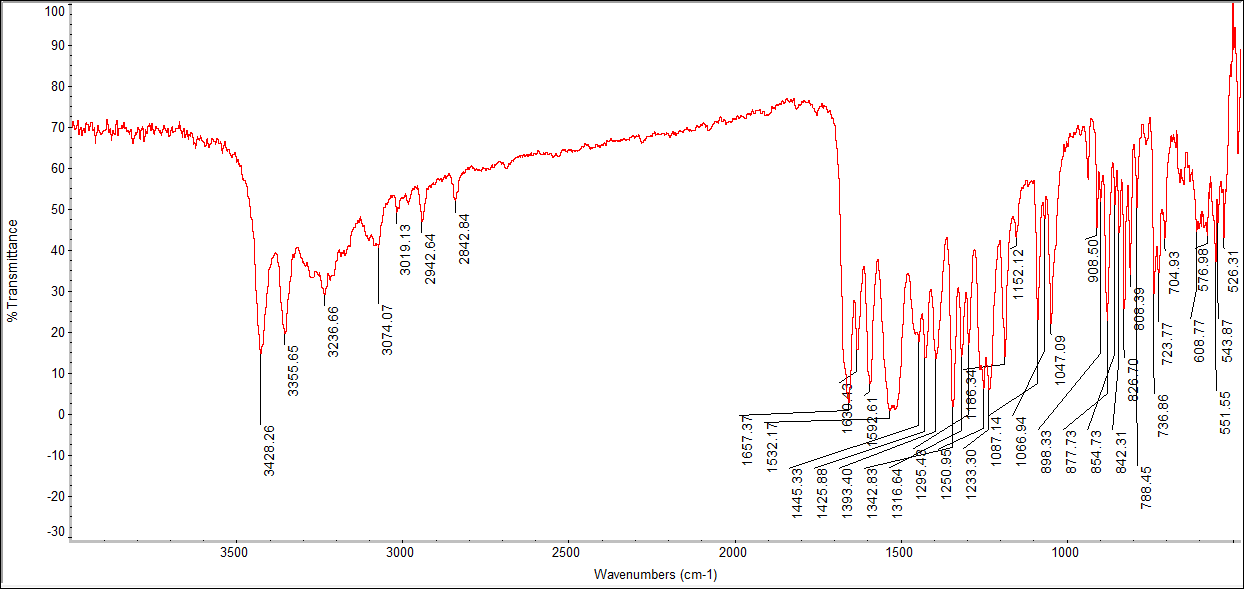


**Figure S52**. Mid-infrared absorbance spectrum of *N-*(2-carbamoyl-4-methoxyphenyl)-5-nitrobenzo[*b*]thiophene-2-carboxamide (G43-C3-OMe)**.**

*N-*(2-carbamoylphenyl)-5-nitrobenzofura*N-*2-carboxamide (G43-Furan).

To a dry flask was added 5-nitrobenzofura*N-*2-carboxylic acid (30mg, 0.14mmol), 2-aminobenzamide (18 mg, 0.13 mmol), 1-Ethyl-3-(3ʹ-dimethylaminopropyl)carbodiimide hydrochloric acid salt (37.9 mg, 0.19 mmol), and DMAP (1.6 mg, 0.01 mmol). The mixture was purged with nitrogen for 15 minutes. The flask was then charged with 2 mL of dry dichloromethane and allowed to stir at 23°C for 16 hours resulting in a white precipitate. The reaction mixture was filtered and the solids washed three times with equivalents of dichloromethane. The solids were collected and vacuumed to dryness, yielding (40mg, 95%). ^1^H NMR (400 MHz, dmso-*d*_6_) δ 13.30 (s, 1H), 8.80 (d, *J* = 2.5 Hz, 1H), 8.66 (dd, *J* = 8.4, 1.2 Hz, 1H), 8.46 (s, 1H), 8.36 (dd, *J* = 9.2, 2.4 Hz, 1H), 8.01 – 7.80 (m, 4H), 7.60 (ddd, *J* = 8.5, 7.4, 1.5 Hz, 1H), 7.24 (td, *J* = 7.6, 1.2 Hz, 1H). ^13^C{^1^H} NMR (151 MHz, dmso-*d_6_*) δ 170.84, 157.03, 155.34, 151.53, 144.31, 139.01, 132.66, 128.79, 127.86, 123.41, 122.66, 120.31, 119.74, 119.51, 113.03, 111.67. IR: 3429.8, 3331.4, 3081.4, 1674.3, 1654.1, 1620.6, 1584.2, 1519.3, 1449.9, 1344.7, 1317.3, 1287.1, 1263.6, 1174.8, 1122.1, 1073.5, 876.6, 818.0, 761.5, 741.1, 684.5, 630.7, 434.5. HRMS TOF ES+ [C_16_H_11_N_3_O_5_ + Na]^+^ : Calculated: 348.0596 Found: 348.0603.

**Figure S53**. ^1^H NMR spectrum of *N-*(2-carbamoylphenyl)-5-nitrobenzofura*N-*2-carboxamide (G43-Furan).

**Figure S54**. ^13^C{^1^H} NMR spectrum of ^1^H NMR spectrum of *N-*(2-carbamoylphenyl)-5-nitrobenzofura*N-*2-carboxamide (G43-Furan).


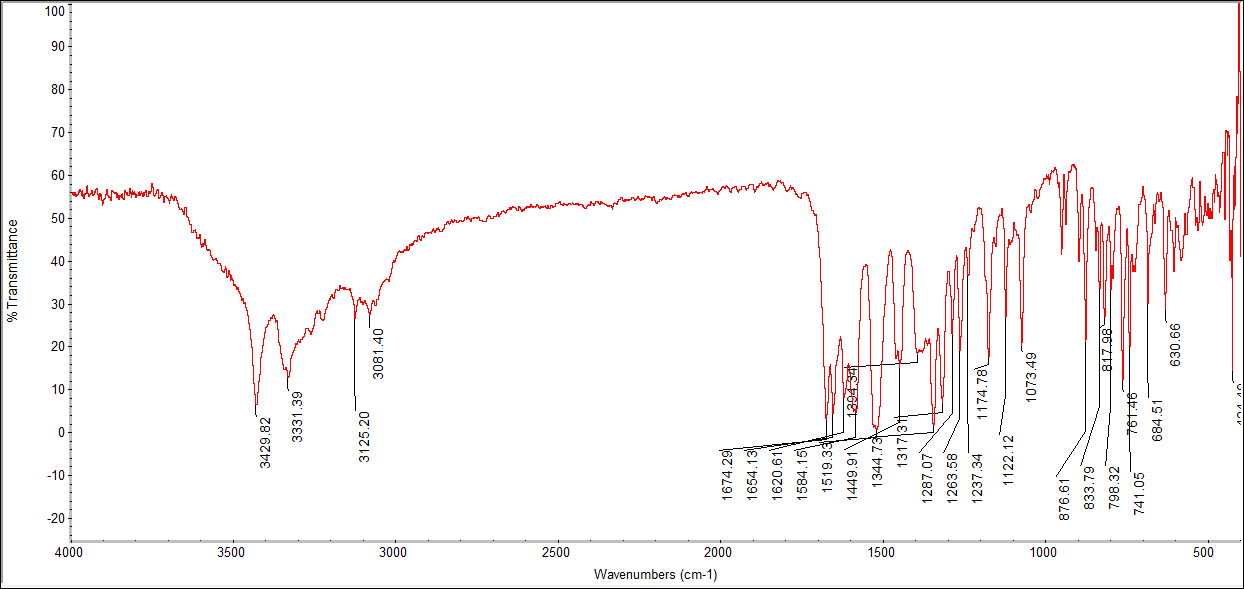


**Figure S55**. Mid-infrared absorbance spectrum of ^1^H NMR spectrum of *N-*(2-carbamoylphenyl)-5-nitrobenzofura*N-*2-carboxamide (G43-Furan).

5-ammonium-*N-*(2-carbamoylphenyl)benzo[*b*]thiophene-2-carboxamide acetate (G43-NH_3_OAc).

To a dry flask were added *N-*(2-carbamoylphenyl)-5-nitrobenzo[*b*]thiophene-2-carboxamide (276mg, 0.81 mmol), 8 mg Palladium on carbon (Pd/C, 10 wt. % Pd loading on a matrix activated carbon support), and 20mL of 190 proof EtOH. The reaction mixture was purged with H_2_ three times using a balloon fitted to the flask. The reaction vessel with a balloon was charged a final time (H_2_, ~300mL, 1atm) and allowed to stir overnight at 23°C. The reaction was judged complete by TLC (EtOAc, R_f_^product^ = 0.67, fluorescent yellow under long-waved UV (λ=365nm)). The reaction mixture was filtered through diatomaceous earth, and rinsed twice with aliquots of THF. The resulting solution was concentrated via vacuum to a slightly yellow oil which was protonated with 100x excess of glacial acetic acid, then vacuum dried to a tan solid. The solid was recrystallized from hot H_2_O to yield pure product (243mg, 81%). ^1^H NMR (600 MHz, dmso-*d*_6_) δ 9.05 (d, *J* = 2.2 Hz, 1H), 8.59 (d, *J* = 8.3 Hz, 1H), 8.49 (s, 1H), 8.37 (d, *J* = 8.9 Hz, 1H), 8.29 (d, *J* = 8.4 Hz, 2H), 7.95 (d, *J* = 7.8 Hz, 1H), 7.91 (s, 1H), 7.60 (t, *J* = 7.8 Hz, 1H), 7.23 (dd, *J* = 9.4, 6.2 Hz, 3H). ^13^C{^1^H} NMR (151 MHz, DMSO) δ 171.48, 159.48, 146.63, 145.98, 143.99, 139.89, 139.42, 133.21, 129.30, 126.39, 124.84, 123.74, 121.91, 120.87, 120.71, 119.64.. IR: 3465.6, 3340.5, 1650.2, 1617.0, 1594.8, 1527.2, 1450.8, 1394.1, 1311.5, 1246.1, 865.5, 745.2. HRMS TOF ESI+ [C_16_H_14_N_3_O_2_S + Na]^+^ : Calculated: 334.0626 Found: 334.0623.

**Figure S56**. ^1^H NMR spectrum of 5-ammonium-*N-*(2-carbamoylphenyl)benzo[*b*]thiophene-2-carboxamide acetate (G43-NH_3_OAc).

**Figure S57**. ^13^C{^1^H} NMR spectrum of 5-ammonium-*N-*(2-carbamoylphenyl)benzo[*b*]thiophene-2-carboxamide acetate (G43-NH_3_OAc).


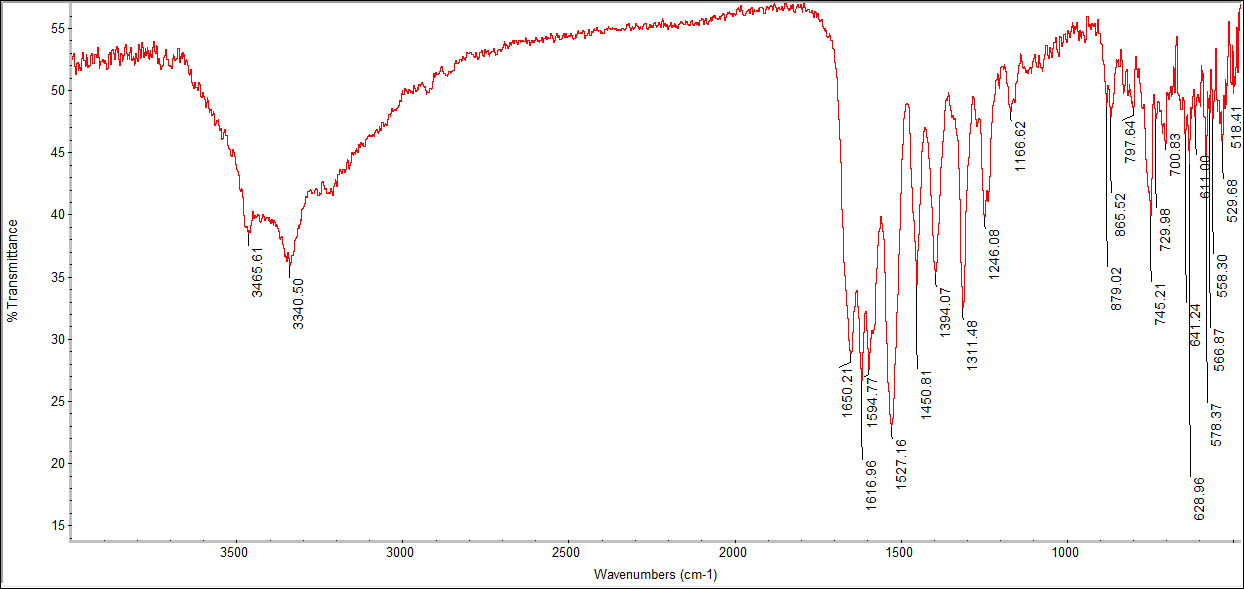


**Figure S58**. Mid-infrared absorbance spectrum of 5-ammonium-*N-*(2-carbamoylphenyl)benzo[*b*]thiophene-2-carboxamide acetate (G43-NH_3_OAc).

# Bibliography

[1] B. Nijampatnam, P. Ahirwar, P. Pukkanasut, H. Womack, L. Casals, H. Zhang, X. Cai, S. M. Michalek, H. Wu, S. E. Velu, *ACS Med. Chem. Lett.* **2021,** *12* (1), 48-55.

[2] Q. Zhang, B. Nijampatnam, Z. Hua, T. Nguyen, J. Zou, X. Cai, S. M. Michalek, S. E. Velu, H. Wu, *Scientific Reports* **2017**, *7*, 5974.

[3] M. H. Al-Huniti, J. Rivera-Chávez, K. L. Colón, J. L. Stanley, J. E. Burdette, C. J. Pearce, N. H. Oberlies, M. P. Croatt, *Organic Letters* **2018**, *20*, 6046-6050.

[4] M. L. Keshtov, A. L. Rusanov, S. V. Keshtova, P. V. Petrovskii, A. A. Shchegolikhin, *Russian Chemical Bulletin* **2002**, *51*, 117-123.

[5] D. D. Mukerjee, S. K. Shukla, B. L. Chowdhary, *Archiv der Pharmazie* **1981**, *314*, 991-994.

[6] K. Ito, S. Ito, T. Shimamura, S. Weyand, Y. Kawarasaki, T. Misaka, K. Abe, T. Kobayashi, A.D. Cameron, S. Iwata, *Journal of Molecular Biology* **2011**, 408, 177-186.
